# Analysis of proteins and peptides to investigate the molecular signatures of a conventional antidiabetic herb, *Boerhavia procumbens* Banks ex Roxb. (*Nyctaginaceae*)

**DOI:** 10.1038/s41598-025-16474-8

**Published:** 2025-12-31

**Authors:** Ghazala Hassan, Shazia Anjum, Samina Ejaz, Muhammad Ashraf, Ayesha Momen

**Affiliations:** 1 Institute of Chemistry, Faculty of Chemical & Biological Sciences, The Islamia University of Bahawalpur, Bahawalpur, 63100 Pakistan; 2 Department of Biochemistry & Molecular Biology, Institute of Biochemistry, Biotechnology & Bioinformatics (IBBB), Faculty of Chemical & Biological Sciences, The Islamia University of Bahawalpur, Bahawalpur, 63100 Pakistan; 3 International Center for Chemical and Biological Sciences, H.E.J. Research Institute of Chemistry, Karachi University, Karachi, 75270 Pakistan; 4 Govt. Sadiq College for Women University (GSCWU), Bahawalpur, Pakistan

**Keywords:** *Boerhavia procumbens*, LC-MS analysis, Antidiabetic proteins, γ-conglutin, Thioredoxin peroxidase, Zerumbone, Biochemistry, Chemistry

## Abstract

*Boerhavia procumbens* Banks ex Roxb. (Nyctaginaceae) is a diuretic herb that is traditionally used to treat diabetes mellitus, a disease having an alarmingly high prevalence rate worldwide. Although various micro molecules present in *B. procumbens* are known for their antidiabetic roles, the macromolecular constituents have not been studied yet. The current study was therefore planned to identify and characterize the antidiabetic proteins/peptides, if any, present in *B. procumbens.* Initially the proteins/peptides were extracted, from roots and whole plant of *B. procumbens*, in sodium phosphate buffers each of concentration 200 mM and of variable pH (range 6–8). The crude extracts were primarily tested for their inhibitory potential against α-glucosidase and α-amylase. The crude extracts with significant inhibitory potential were processed for partial purification of proteins/peptides through the ammonium sulphate (NH_4_)_2_SO_4_ precipitation method. The precipitated proteins/peptides were purified through dialysis, and purified fractions, followed by protein quantification, were tested for anti-α-glucosidase and anti-α-amylase activity. The proteins/peptides mixtures were resolved on SDS PAGE, and prominent gel bands were cut and subjected to LC-MS/MS. All crude extracts were found to strongly inhibit both enzymes (i.e., α-glucosidase and anti-α-amylase). Only 03 proteins/peptides fractions were inhibitory to α-glucosidase (with IC_50_ range of 137.8 ± 1.2 µg/mL- 352.3 ± 1.2 µg/mL), while 09 fractions were active against α-amylase (with IC_50_ range of 24.24 ± 1.8 µg/mL to 85.55 ± 4 µg/mL). A total of 20 proteins were identified via LC-MS/MS analysis, including 04 well-known antidiabetic proteins, i.e., γ-conglutin, thioredoxin peroxidase, 7S globulin, and Zerumbone synthase enzyme. Our study has provided reliable molecular evidence that assures the antidiabetic potential of *B. procumbens* and suggests the future extension of work for the purification, functional characterization and therapeutic application of antidiabetic proteins.

**Supplementary Information:**

The online version contains supplementary material available at 10.1038/s41598-025-16474-8.

## Introduction

Diabetes mellitus (DM) is a group of metabolic disorders characterized by high blood sugar (glucose) levels, a situation that arises due to defective insulin secretion, action, or both. The signs vary among different stages and patients, but some symptoms may indicate diabetes, such as unexplained weight loss, regular fatigue, frequent infections in the genital areas or urinary tracts^1^kidney disease, cardiovascular disease, diabetic ketoacidosis (DKA), and hyperosmolar hyperglycemic state, causing great suffering for patients^2^. According to the International Diabetes Federation, it is predicted that the prevalence of diabetes will rise to 10.2% (578 million) by 2030 and 10.9% (700 million) by 2045^3^.

A variety of plants have been reported to have antidiabetic potential either in crude form or purified phytochemical form. Some plant-based proteins are known to possess antidiabetic potential. Polypeptides isolated from *Panax ginseng* Meyer’s root, *Cystoseira barbata’s* leaves, *Momordica charantia*’s fruits and seeds, proteins isolated from *Acacia melanoxyloni’s* seeds, *Glycine max*’s grains, *Oryza sativa*’s rice bran, *Momordica cymbalaria*’s fruits and seeds, and *Bauhinia retusa* L.’s seeds are proven antidiabetic plant materials^4^. A peptide purified from the cowpea plant (*Vigna unguiculata*) has an amino acid sequence similar to that of the bovine insulin and thus possesses insulin-like properties^5^A protein obtained from turmeric (*Curcuma longa* L.) is reported to be an α-glucosidase and α-amylase inhibitor^6^. An insulin-type protein isolated from roots, seeds, and fruit of *Moringa oleifera*^7^while polypeptide fractions isolated from leaves of *Cystoseira barbata* by gel filtration, etc., have a great hypoglycemic effect^8^. From conventional to synthetic agents, plants deliver a latent source of hypoglycemic drugs and are extensively used to prevent diabetes. Generally, herbal drugs are well tolerated by the patient, having rarer unpremeditated results, fewer side effects than traditional medicine, and may be nontoxic to use. Herbal drugs are more effective for long-lasting health problems and less costly than synthetic medications^9^.

Cholistan desert covers an area of 26,000 km^2^present within South of Bahawalpur, Punjab, Pakistan. It extends through the Nara and Thar deserts of Sindh between 69˚52ʹE and 75˚24ʹE longitude and 27˚42ʹN and 29˚45ʹN latitude^10^ at an altitude of almost 112 m above sea level^11^.The flora of the Cholistan desert has 154 plant species of 106 genera and 38 families^12^.

**Fig. 1:**
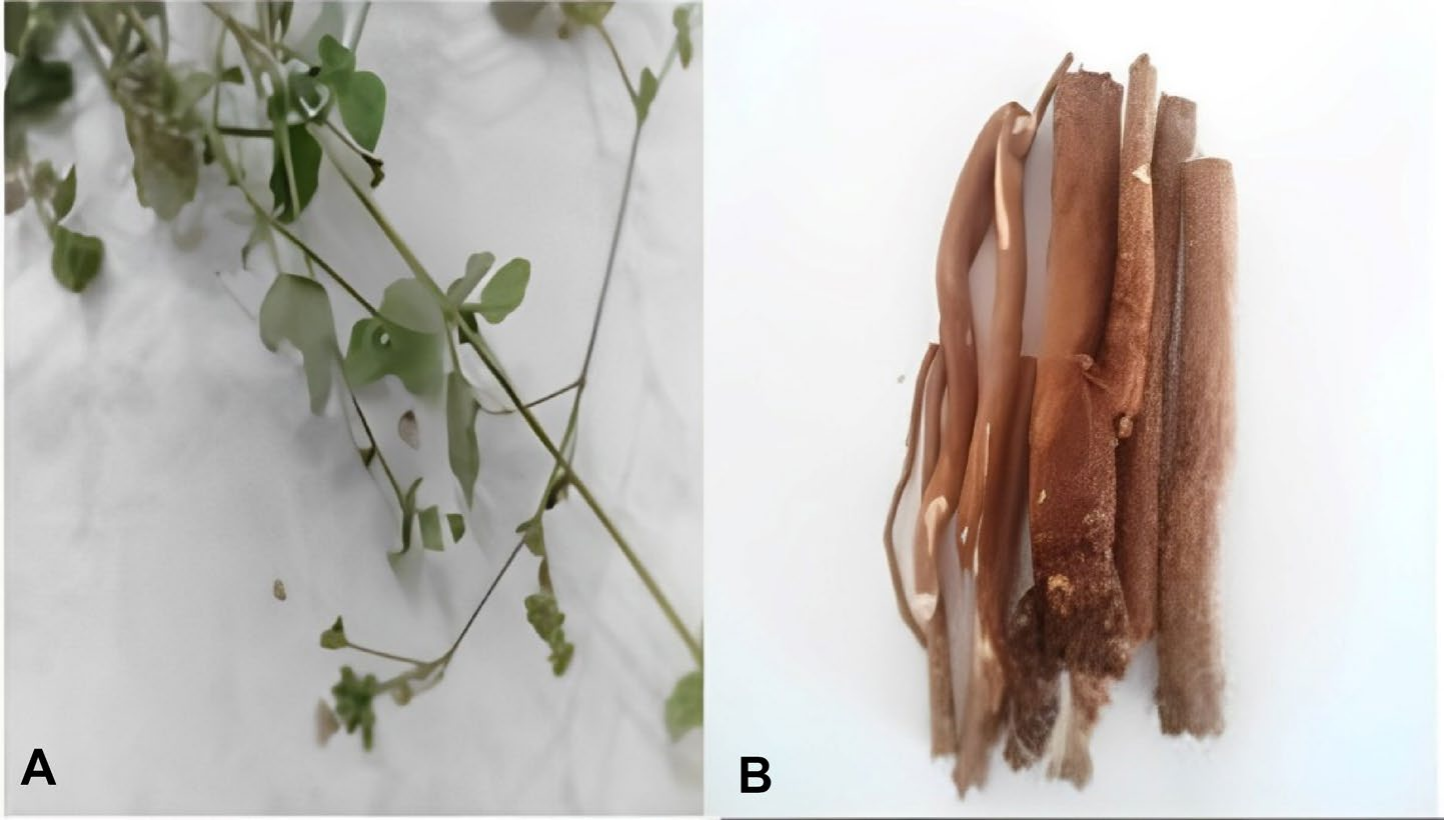
*B. procumbens*: Whole plant (**A**), Roots (**B**).

*Boerhavia procumbens* belongs to the kingdom Plantae, Division Tracheophyta, Class Magnoliopsida, Order Caryophyllales, and family Nyctaginaceae. The Nyctaginaceae family, also known as the 4 o’clock family, has 30 genera and 290 species. In Pakistan, there are about 5 genera and 11 species. Generally, the genus *Boerhavia* is well known for the treatment of jaundice, hepatic disorder, scanty urine, diabetes, cancer, and malaria^13^. The roots and leaves of *B. procumbens* are used for the treatment of various diseases like jaundice, stomach disorders, asthma, and sore throat, antispasmodic and analgesic^14^antidiabetic^15^anti-convulsant activity^16^purification of blood^17,18^anti-inflammatory, anti-oxidants, and anti-asthmatics effects^19^. The whole plant of *B. procumbens* is traditionally used for the treatment of inflammation, bronchitis, piles, anemia, night blindness, asthma, and cough^20,21^. Different parts of the *B. procumbens* plant used in this study are shown in Fig. 1.

A variety of chemical constituents, such as flavonoids, alkaloids, phenolics, and cardiac glycosides, are present in this plant^22^. Similarly, plenty of bioactivities such as anti-inflammatory, anti-oxidants, and anti-asthmatics^19^ antidiabetic^15^ have been reported from *B. procumbens*. *B. procumbens* is traditionally used for the treatment of diabetes due to antidiabetic roles of many micromolecules isolated from *B. procumbens.* However, to the best of our knowledge the macromolecular components of this plant have not been investigated till date. Knowing that many proteins / peptides, isolated from other plants worldwide, and the traditional use of *B. procumbens* for the treatment of diabetes motivated us to hypothesize that *B. procumbens* contains antidiabetic proteins/peptides that need to be explored and evaluated for therapeutic purposes. It was further speculated that proteins/peptides of this plant may be more effective and safer alternative than the micromolecular synthetic drugs. Hence, the present study was initiated to extract, identify, partially purify and characterized the proteins / peptides present in *B. procumbens*, a medicinal plant from Cholistan desert. To the best of our knowledge, the proteins / peptides of Cholistan desert’s plants have not been studied yet. This study is the first to report the bioactive proteins/peptides extracted from *B. procumbens* and thus laid foundation for the similar future molecular evaluation of folk medicinal plants of Cholistan desert.

## Materials and methods

**Fig. 2:**
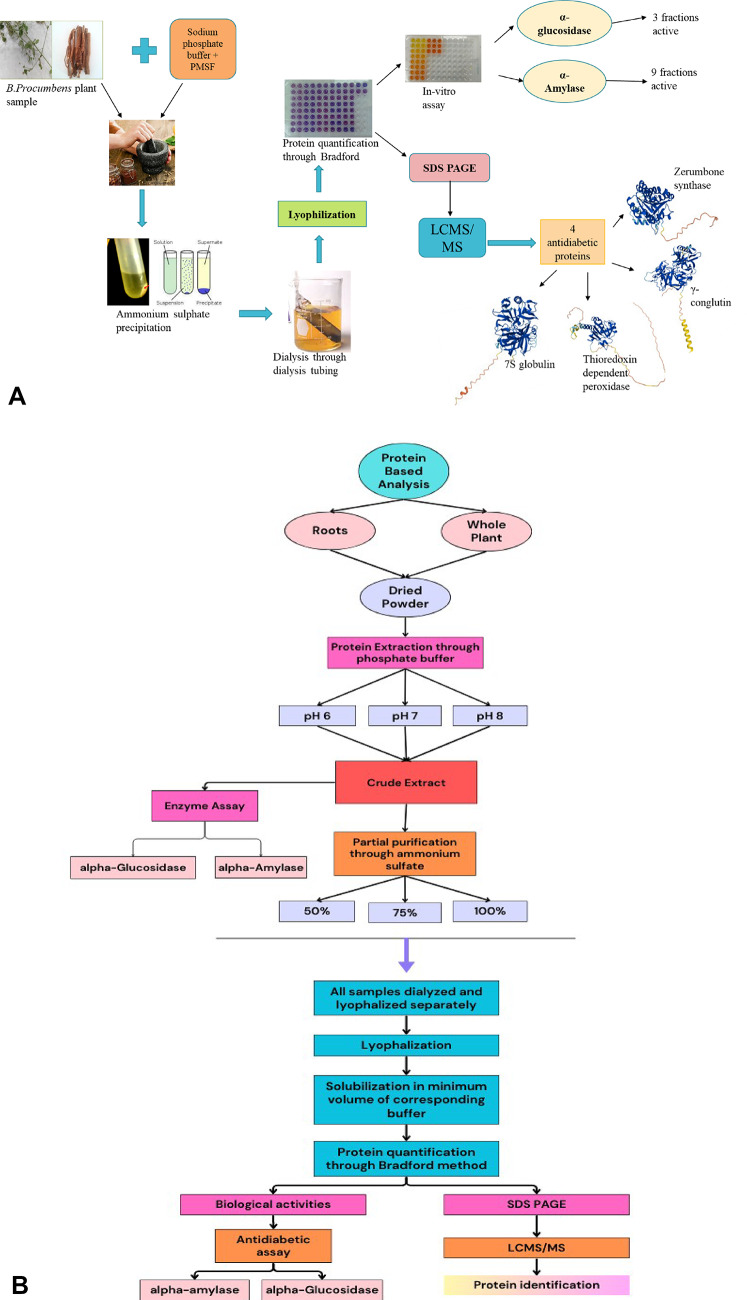
Flow sheet diagram for experimental procedures employed during study. (**A**) Graphical illustration of experimental methodology. (**B**) Schematic representation of experimental methology.

The overall procedure used during the present study is described in the flow sheet diagram (Fig. 2). The dried powdered form of roots and whole plant of *B. procumbens* was used for protein-based analysis. For this purpose, extraction was done in 3 different pH levels, ranging from pH 6–8, separately by macerating 40 g of powdered form plant material in extraction buffer (198 mL sodium phosphate buffer + 2 mL PMSF) for 10–15 min. The crude extract obtained was proceeded for antidiabetic assays (α-glucosidase and α-amylase) as well as for partial purification through ammonium sulphate, from which fractions were obtained at 50%, 75%, and 100%. All fractions were dialyzed through dialysis tubing, then lyophilized and dissolved in a minimum amount of the corresponding buffer. The quantification of proteins in these fractions was done through the Bradford method. Then, these fractions were used for antidiabetic assays (α-glucosidase and α-amylase). On the other hand, these fractions were also subjected to SDS PAGE. Prominent bands were cut and proceeded for LC-MS/MS, where proteins were identified. In this study, 4 proteins were identified as antidiabetics.

### Collection of plant

Roots and the whole plant of *B. procumbens* were collected from the Baghdad-ul-Jadeed campus of the Islamia University of Bahawalpur, Pakistan, in November 2022. This plant was identified by Dr. Muhammad Abdullah, Associate Professor at Cholistan Institute of Desert Studies (CIDS), The Islamia University of Bahawalpur, Punjab, Pakistan, and the assigned voucher number is CIDS/ IUB-0203/5.

### Processing of plant material

The plant material was washed thoroughly with water and shade-dried for two weeks. The dried plant material was ground to a fine powder and used as the source material for further analysis.

### Extraction of protein

Extraction of protein was done in sodium phosphate buffer of different pH levels, ranging from 6 to 8, maintaining the temperature at 4 °C. For the preparation of sodium phosphate buffer, 0.2 M monobasic sodium phosphate was made by dissolving 27.8 g in 1 L distilled water (X), and 0.2 M dibasic sodium phosphate was made by dissolving 53.65 g in 1 L distilled water(Y). Both solutions (X and Y) were mixed in different proportions, as mentioned in Table 1, and pH was adjusted using conc. HCL solution.

**Table 1:** Composition of sodium phosphate buffers.

Sr. no.	pH required	0.2 M monobasic sodium phosphate (mL)	0.2 M dibasic sodium phosphate (mL)	Distill water (mL)	Total volume (mL)
1	6	85	15	100	200
2	7	39	61	100	200
3	8	5.3	94.7	100	200

Phenylmethylsulphonyl fluoride (PMSF) is used as a protease inhibitor, which reduces the chance of protein degradation due to reactions of peptidase and provides a higher concentration of functional and intact proteins^23^. To ensure the integrity and quantity of extracted protein, 0.1 M PMSF was added to the buffer for this purpose. A 0.1 M solution of PMSF was prepared by dissolving 1.74 g of PMSF in 100 mL of ethanol. Extraction was done in 3 different pH levels, ranging from pH 6–8, separately by macerating 40 g of powdered form plant material in extraction buffer (total volume = 200mL (198mL sodium phosphate buffer + 2 mL PMSF)) for 10–15 min. The homogenate was filtered through a muslin cloth, and the filtrate collected in a beaker was subjected to centrifugation at 4 °C, 6000 rpm for 10 min to remove cellular debris. The supernatant was collected and stored in aliquots at 30 °C.

### Partial purification of protein using ammonium sulfate precipitation method

**Fig. 3:**
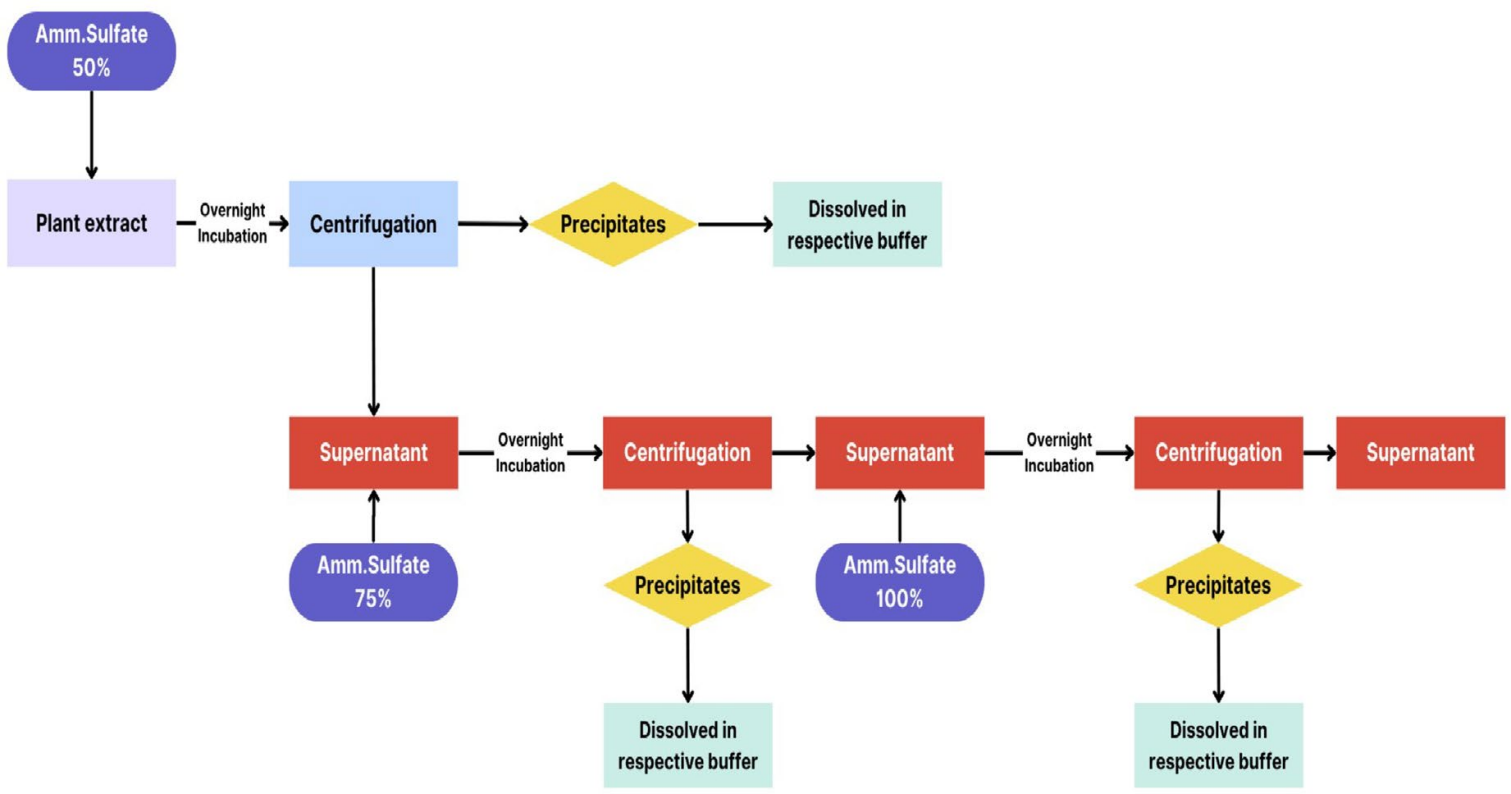
Ammonium sulphate precipitation method used for precipitation of proteins from crude plant extracts.

Ammonium sulfate is the most commonly used precipitant for salting out the proteins. Protein purification from a solution is frequently achieved by the use of ammonium sulfate precipitation. Proteins exposed polar and ionic groups allow them to establish hydrogen bonds with water molecules in solution. Ammonium sulfate and other tiny, highly charged ions compete with the proteins to attach to the water molecules when introduced in high doses. Precipitation occurs as a result of the protein losing its water molecules and becoming less soluble. A specific amount of ammonium sulfate should be added to the protein solution to get the required saturation^24^. For 50% saturation, 60 g ammonium sulfate was added to the 200 mL of the cell debris-free extract obtained in the previous step, and the mixture was incubated overnight in 4 °C chamber. The next day, the solution containing precipitated proteins was centrifuged at 10,000 rpm and 4 °C for 20 min. Protein pelleted down at the bottom of the centrifuge tube was separated from supernatant and stored at 4 °C. The supernatant was further for 75% and 100% saturation, and for this purpose, the same procedure was repeated by adding 36 g and 42.2 g (NH_2_)_2_SO_4,_ respectively. Finally, the protein pellets were dissolved in phosphate buffers of respective pH. The procedure scheme is shown in Fig. 3.

### Desalting of proteins by dialysis

All the protein pellets were solubilized in appropriate pH buffers, and the protein solutions thus obtained were dialyzed to remove contaminants, excess of salt (NH_4_)_2_SO_4,_ and unwanted compounds from the protein solution by selective and passive diffusion through a semipermeable membrane. Dialysis tubing of flat width 23 mm, wall thickness 28 μm, and MWCO 6000- 8000d (Fisherband, Catalog # 9612) was used for dialysis. A small piece (2 inches) of dialysis tubing was cut and dipped in water for a few seconds. By rubbing the dipped dialysis tubing, it opened. One end of the tube was tied with a knot, protein solution was added to the tube, and the second end of the tube was tied with thread. Dip it in distilled water and leave it on, stirring at 4 °C for 5 hours^7^. To maintain the integrity of proteins, dialysis was carried out at a low temperature using an ice-cold buffer, and the temperature was continuously monitored.

Moreover, after dialysis, proteins were analyzed through gel electrophoresis, and the presence of intact bands indicated the least damage, if any, caused to the protein.

### Quantification of proteins

Extracted proteins were quantified using the Bradford assay. The binding of Coommassie brilliant blue G-250 with protein causes an absorbance shift^25^. The precipitated proteins were dissolved in phosphate buffer, and the protein contents in solution were determined through the Bradford assay. The Bradford reagent contains Coommassie brilliant blue dye G-250, which specifically interacts with protein and causes an absorbance shift. The binding of a dye molecule, Coommassie Brilliant Blue, to proteins results in a shift in the dye’s absorption spectrum. When the dye binds to protein, its absorption maximum shifts from 465 nm to 595 nm, resulting in a change in color from brown to blue. In the Bradford assay, the 1% bovine serum albumin was used as a positive control. Various compositions with different concentrations of proteins were prepared and proceeded with the extracted protein samples.

**Fig. 4:**
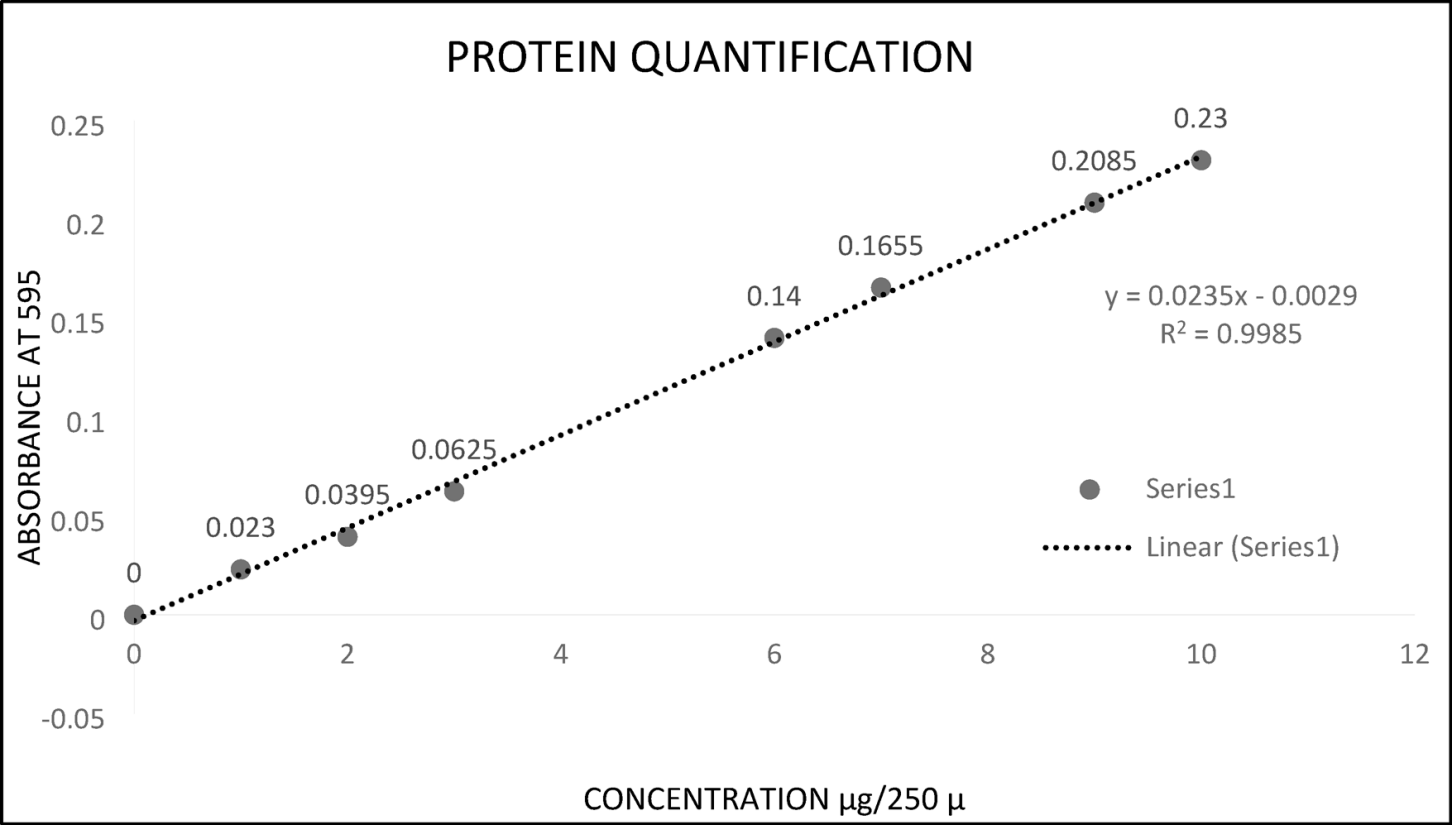
Standard curve for the estimation of proteins in crude extracts and ammonium sulphate precipitated proteins/peptides fractions of *B. procumbens*.

Standards of varying protein (BSA) concentration ranging from 0 µg to 15 µg were prepared in a microtiter plate, and the volume was made 150 µL by adding saline solution.100 µL of Bradford reagent was added to each standard. Sample proteins were loaded on the same microtiter plate (5 µL of each protein extract, 145 µL of saline solutions, and 100 µL of Bradford reagent). The plate was incubated for 10 min at room temperature. Absorbance was measured at 595 nm using an Epoch 2.0 reader integrated with Gen 5 V 2.03 software. The graph was plotted between absorbance and BSA standard concentration to get a standard curve (Fig. 4). Using a regression equation, the protein content present in samples was calculated from the absorbance.

$${\text{y}} = {\text{cx}} - {\text{slope}}$$

Here, y = Optical density; x = Concentration; y = 0.0148x + 0.5426; x = (y − 0.5426)/0.0148.

The concentration of all protein samples was calculated by putting the absorbance value of each protein sample in this equation.

### Analysis of protein fractions for antidiabetic potential

#### Quantitative estimation of α-glucosidase inhibition

The α-glucosidase assay was carried out as reported in a previously published article^26^. The assay contained 50 mM phosphate buffer (pH 6.8, 70 µL), 0.5 mM test compound (10 µL), and enzyme solution (0.02 units, 10 µL) to make a total volume of 100 µL. 10 µL of substrate PNPG was used to start the reaction. All the contents were mixed up properly, pre-incubated for 10 min at 37 °C, and pre-reading was taken at 400 nm. Yellow colour absorbance indicates the formation of *p*-nitrophenol. An Epoch (Bio Tek, USA) 96-well microplate reader was used to calculate the absorbance at 400 nm. Quercetin was used as a positive control. The experiments were carried out in triplicate. EZ-Fit enzyme kinetics software version 5.0 purchased from Perrella Scientific Inc., Amherst, USA, was used to calculate the IC_50_ values of suitable fractions. Other versions of the EZ-Fit are accessible on URL: https://www.mathworks.com/matlabcentral/fileexchange/10176-ezyfit-2-44. The percentage inhibition was calculated as follows:

$${\text{Inhibition}}\left( \% \right) = {\text{Abs}}.\;{\text{of}}\;{\text{Control}} - \left( {{\text{Abs}}.\;{\text{of}}\;{\text{sample}}/{\text{Abs}}.\;{\text{of}}\;{\text{Control}}} \right) \times {\text{1}}00$$

#### Quantitative estimation of α-amylase inhibition

The protocol for α-Amylase assay was taken from a previous article^27^. This assay contained 0.1% starch (1.5 mL), phosphate buffer pH 7 (1.0 mL), and enzyme source (0.5 mL) to make a total volume of 3 mL. As the enzyme was added, the reaction started. So all contents were mixed and kept at 37 °C for 10 min. After the given time, 400 µL of DNS was added to the tube and boiled in a water bath. The reaction was carried out in triplicate. Readings were taken at 540 nm Epoch (Bio Tek, USA). The same procedure was first repeated with 3 standard antidiabetic drugs, Acarbose, Metformin, and Empagliflozin, and then with test samples (protein extract of *B. procumbens*) at different concentrations to check their α-amylase inhibition capacity. Comparison was observed between standard drugs and protein samples. GraphPad Prism 8 software was used to calculate the IC_50_ values of suitable fractions. The percentage inhibition was calculated as follows:

$${\text{Inhibition}}\,(\% ) = {\text{Abs}}.\;{\text{of}}\;{\text{Control}} - ({\text{Abs}}.\;{\text{of}}\;{\text{sample}}/{\text{Abs}}.\;{\text{of}}\;{\text{Control}}){\text{ }}$$

### SDS PAGE analysis of purified proteins

Protein samples were analyzed through sodium dodecyl sulphate polyacrylamide gel electrophoresis (SDS PAGE) and visualized by staining with Coommassie brilliant blue^28^. For this purpose, gel plates were washed and assembled with spacers. Gel solutions of sealing, resolving, and stacking gel were prepared (details mentioned in supplementary data Table 1) and poured in the inter-plate space. Each gel solution was allowed to polymerize for 20–30 min at room temperature after being poured. Plates with polymerized gels were placed in a gel running tank. Tris-Glycin running buffer (pH 8.3) was poured into both portions of the tank. For sample preparation, 80 µg of each sample was mixed with 5 µL loading dye and 2 µL DTT solution, and then distilled water was added to make the volume of all samples 80 µL. The sample mixtures were then heated at 90 °C for 5 min and were loaded into wells. 80 µL of Molecular Weight Marker (Thermo Scientific, Catalogue # 26610) was loaded into the first well. Gel was electrophoresed at a voltage 120 volts provided by the battery and stopped when tracking dye reached at sealing gel.

### Staining of gel

#### Silver staining

Silver staining was performed by following the standard protocol of silver staining given by Mireille Chevalet^4^ with slight modifications. Firstly, the gel was kept overnight and poured in fixation solution (50 mL of Methanol, 10 mL of acetic acid, and 40 mL H_2_O). The next day gel was incubated in a solution of 5% methanol and 7% acetic acid for 30 min and then in 10% glutaraldehyde solution for 30 min. Gel was washed thoroughly with ddH_2_O several times until the odour of glutaraldehyde subsided, and gel was incubated in DTT solution (0.002 g DTT in 100 mL H_2_O) for 30 min and then in 0.1% silver nitrate solution for 45 min. Followed by rinsing thoroughly with ddH_2_O, Gel was immersed in developer solution (0.15 mL of 37% formaldehyde in 100 mL of 3% sodium carbonate) for 30 min and rotated gently, when band starts getting brown of desired intensity stop solution (2.3 M citric acid and 0.03% sodium carbonate) was added. The resultant gel was placed under a UV illuminator, and an image was acquired with the camera.

**Fig. 5:**
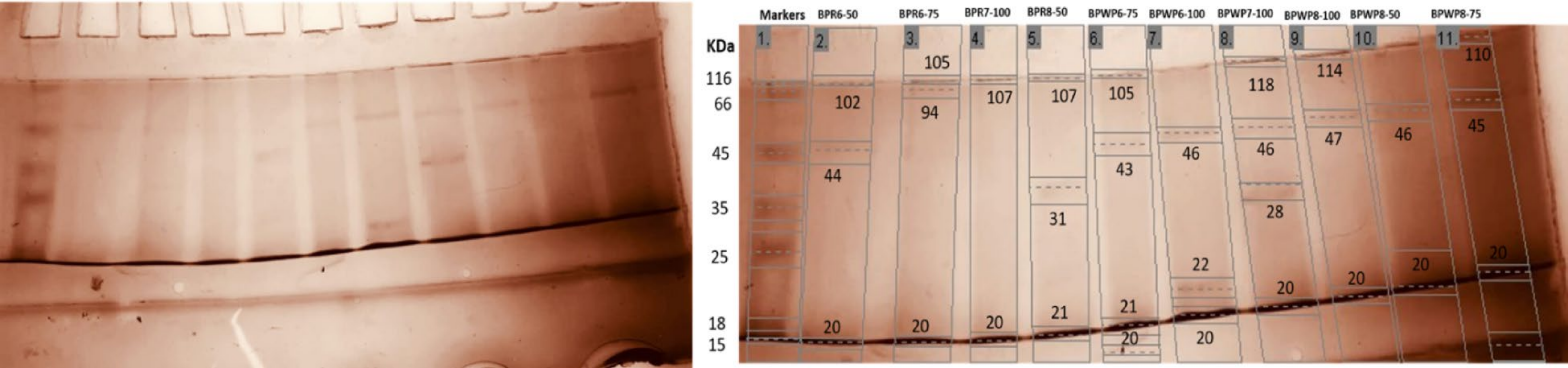
Silver stained SDS PAGE gel image before (left) and after (right) analysis through gel analyzer.

Gel picture after Coommassie staining and silver staining shown in Fig. 5.

### LC-MS/MS analysis

The gel bands visible, followed by a staining sequence, were cut and submitted for in-gel digestion of protein and sequencing of the generated tryptic digest by LC-MS/MS spectrometry (the faculty of Harvard University, USA). A database search was conducted to determine sequence homologies by using NCBI BLAST.

The removed gel bands were divided into fragments that were around 1 mm3. After that, gel particles underwent an altered in-gel trypsin digesting process^29^. Acetonitrile was used to wash and dehydrate the gel fragments for ten minutes before the acetonitrile was removed. After that, the pieces were thoroughly dried in a speed vac. After rehydrating the gel fragments, 12.5 ng/µL of modified sequencing-grade trypsin (Promega, Madison, WI) was added to a 50 mM ammonium bicarbonate solution and heated to 4 °C. After 45 min, the surplus trypsin solution was discarded, and the gel fragments were barely covered with 50 mM ammonium bicarbonate solution. After that, samples were kept overnight at 37 °C. The ammonium bicarbonate solution was subsequently removed, and the peptides were then extracted with one wash using a solution containing 50% acetonitrile and 1% formic acid. Next, the extracts were dried in a speed vacuum for about an hour. Following that, the samples were kept at 4 °C until analysis.

The samples were reconstituted in 5–10 µL of HPLC solvent A (2.5% acetonitrile, 0.1% formic acid) on the day of analysis. Packing 2.6 μm C18 spherical silica beads into a fused silica capillary (100 μm inner diameter x ~ 30 cm length) with a flame-drawn tip produced a nano-scale reverse-phase HPLC capillary column^30^. Each sample was loaded using a Thermo EASY-LC (Thermo Fisher Scientific, Waltham, MA) once the column had stabilized. Peptides were eluted using solvent B at progressively higher concentrations (99% acetonitrile, 0.1% formic acid) once a gradient had been created. After being eluted, the peptides were placed into an Orbitrap Exploris480 mass spectrometer (Thermo Fisher Scientific, Waltham, MA) and electrosprayed to ionize them. Each peptide was found, separated, and broken up into distinct fragment ions, resulting in a tandem mass spectrum. Protein databases and the obtained fragmentation pattern were compared to identify peptide sequences and, consequently, protein identification using the Sequest software (Thermo Fisher Scientific, Waltham, MA)^31^. Every database has a reverse copy of every sequence, and the information was screened so that the percentage of peptide false discoveries was limited to one to two%.

### Bio informatics

The 3D structures of proteins, shown in Tables 2, 3 and 4, were obtained from a freely accessible online AlphaFold protein 3D structure database available at URL: https://alphafold.ebi.ac.uk/.

**Table 2:** Proteins/Enzyme detected having antidiabetic and antioxidant activity.

Sr no.	Detected proteins	Alphafold database structure	Spectra	Role of detected proteins
1	Zerumbone synthase			Involved in synthesis of zerumbone^32^
				Zerumbone is Anti-cancer, anti-inflammatory, antioxidants, antibacterial, improves insulin sensitivity, reduces the TG level in plasma and hepatic tissues and reduce hepatic steatosis^26,33–39^
2	Gamma conglutin			Antidiabetic^40,41^
3	Thioredoxin peroxiredase			Anti-oxidants, protect pancreatic β-celL^42–44^
4	7S globulin			Antidiabetic, antioxidant, controls glucose level, blood pressure and plasma cholesterol level, and cancer cell and anti-bacterial^45–47^

**Table 3:** Proteins involved in glucose metabolism.

Sr. no.	Detected proteins	Alpha fold database structure	Spectra	Role of detected proteins
1	Phospho pyruvate hydratase			Glycolysis^48^
2	Fructose bisphosphate			Catalyzes the conversion of fructose 1-6-diphosphate to glyceraldehyde 3-phosphate and dihydroxy-acetone phosphate^49^
3	Alpha 1,4 glucan phospho pyruvate			Catalyze an enzyme which the reversible phosphorolysis of α-1,4-linked polysaccharides^50^
4	Aldo keto reductase			Metabolic reactions including reactive aldehyde detoxification, biosynthesis of osmolytes, secondary metabolism, membrane transport, biotic and abiotic stress defense, production of commercially important secondary metabolites, iron acquisition from soil, plant-microbe interactions^51–55^
5	UTP glucose 1 phosphate uridyl transferase			Glycogen biosynthesis^56^
6	Vicilin			Storage protein^57^

**Table 4:** Miscellaneous proteins detected in *B. procumbens*.

Sr. no.	Detected protein	Alphafold database structure	Spectra	Role of detected protein
1	Serine protease			Cleaves peptide bond important in food and biotechnology industry^58^
2	Amine oxidase			Cell differentiation and growth, wound healing, detoxification and cell signaling^59^
3	Legumin			Storage protein^38^
4	β-amylase			Digests carbohydrates, starch etc. to produce sugar i.e. maltose^60^
5	Β-glucosidase			Catalyze hydrolysis of polysaccrides^61^
6	Glyceraldehyde 3-phosphate dehydrogenase			Regulatory function in glucose oxidation^62^
7	Lacto glutathione lyase			Methyloxal detoxification^63^
8	L ascorbate oxidase			Biosynthesis of vitamin C^64^
9	Cysteine proteinases			Plant-pathogen/pest interactions, plant growth and development^65^

### Statistical analysis

The experimental data were subjected to statistical analysis using statistical package for the social sciences (SPSS) version 31. The significance level was calculated via an independent sample t-test.

## Results and discussions

### Quantification of proteins

The quantification of protein extracts was done through the Bradford assay and revealed that sample BPR7-75 had the greatest quantity among all samples. The Protein contents (µg/µL) of all samples are mentioned in the supplementary data Table 2.

### α-glucosidase inhibition activity of protein fractions

**Fig. 6:**
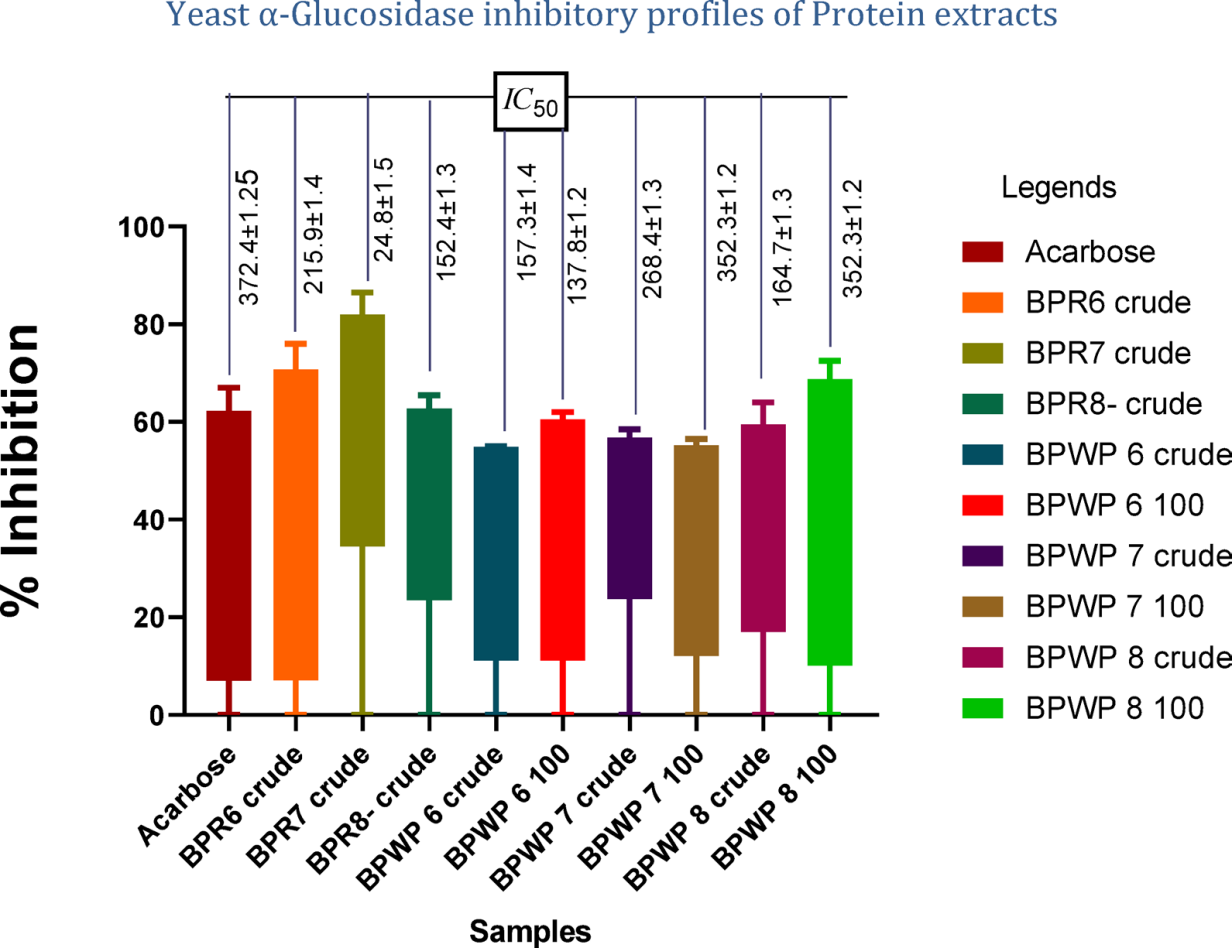
Yeast α-glucosidase inhibitory activity profiles of protein extracts.

The α-Glucosidase inhibition activity of the crude plant extract and protein sample was assessed. Acarbose was utilized as the standard for this purpose, with an IC_50_ of 372.4 ± 1.25 µg/mL. BPR-07 (crude) outperformed all other crude samples in terms of enzyme inhibitory effect with an IC_50_ value of 24.8 ± 1.5 µg/mL and an Inhibition (%) of 85.9 at 0.5 mg/mL. This protein fraction was found to be even better than conventional acarbose. Only three of the ammonium sulphate-precipitated protein samples (BPWP6-100, BPWP7-100, and BPWP8-100) were active, while samples (BPR6-50 to BPWP6-75, BPWP7-50, BPWP7-75, BPWP8-50, and BPWP8-75) showed no activity as given in Fig. 6 and supplementary data Table 3. In comparison to normal acarbose, these results demonstrated that all active samples exhibited much higher % inhibition and lower IC_50_. All of these active protein samples could be a more effective α-glucosidase inhibitor treatment for type 2 diabetes than acarbose.

### α-amylase inhibition assay

**Fig. 7:**
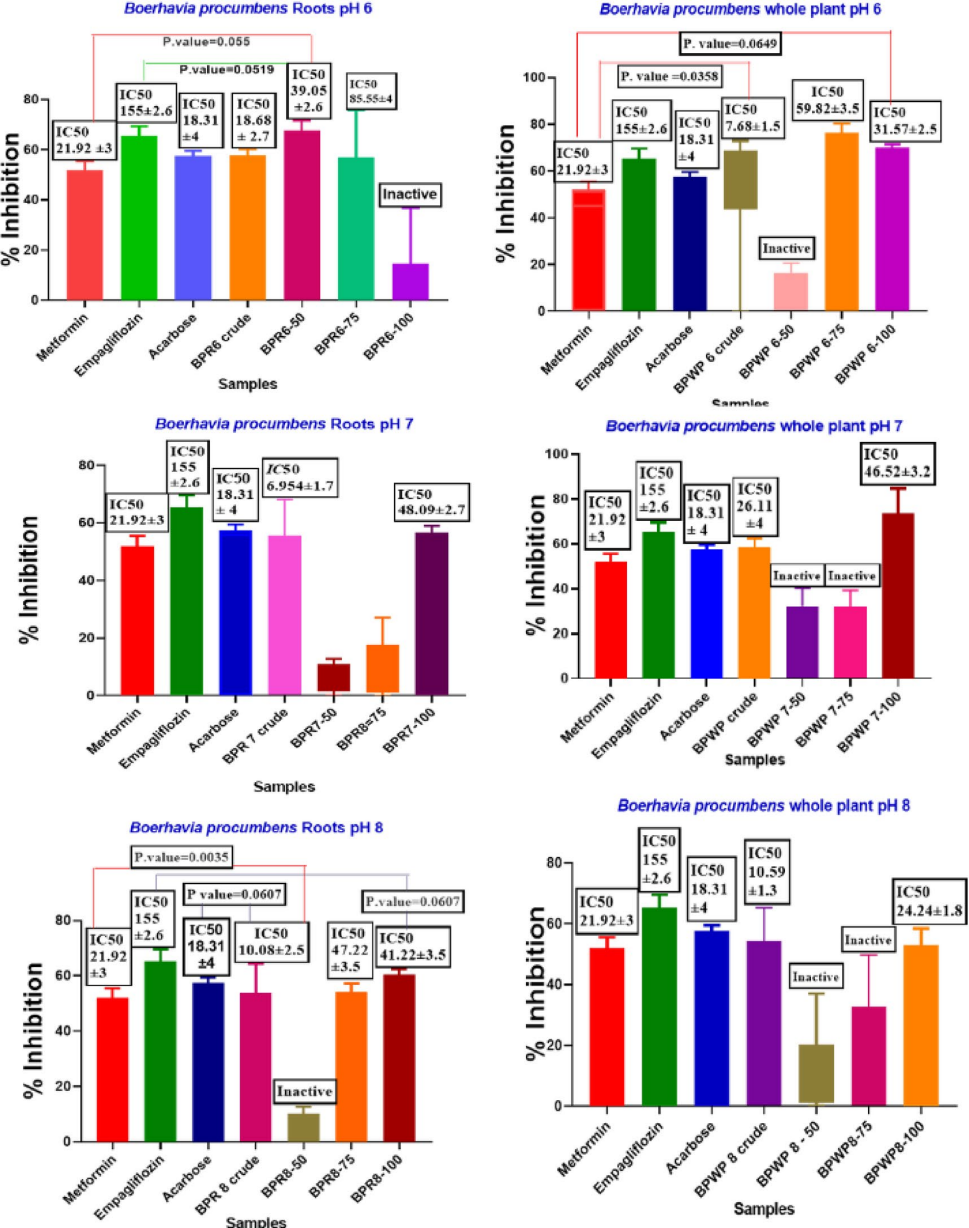
α-Amylase inhibition Assay results of crude plant extract and protein fractions, (**A**) $\:\alpha\:$.Amylase assay results of *Boerhavia procumbens* protein fraction root sample pH 6, crude, and ammonium sulphate precipitation 50%, 75%, 100% comparison with standard Metformin, Empagliflozin and Acarbose. (**B**) $\alpha$.Amylase assay results of *Boerhavia procumbens* protein fraction root sample pH 7, crude, and ammonium sulphate precipitation 50%, 75%, 100% comparison with standard Metformin, Empagliflozin and Acarbose. (**C**) $\alpha$.Amylase assay results of *Boerhavia procumbens* protein fraction root sample pH 8, crude, and ammonium sulphate precipitation 50%, 75%, 100% comparison with Metformin, Empagliflozin and Acarbose. (**D**) $\alpha$.Amylase assay results of *Boerhavia procumbens* protein fraction whole plant sample pH 6, crude, and ammonium sulphate precipitation 50%, 75%, 100% comparison with Metformin, Empagliflozin and Acarbose. (**E**) $\alpha$.Amylase assay results of *Boerhavia procumbens* protein fraction whole plant sample pH 7 crude, and ammonium sulphate precipitation 50%, 75%, 100% comparison with Metformin, Empagliflozin and Acarbose. (**F**) $\alpha$.Amylase assay results of *Boerhavia procumbens* protein fraction whole plant sample pH 8, crude, and ammonium sulphate precipitation 50%, 75%, 100% comparison with Metformin, Empagliflozin and Acarbose.

The ability of each crude plant extract and protein fraction to inhibit α-amylase with an enzyme was examined. Three of the greatest diabetes tablets, acarbose, metformin, and empagliflozin, were utilized for this purpose. Their respective IC_50_ values were 18.31 ± 4 µg/mL, 21.92 ± 3 µg/mL, and 155 ± 2.6 µg/mL, respectively. All crude samples actively inhibited α-amylase, in which BPR-07 and BPWP 6 showed the best α-amylase inhibitory action, while 9 protein fractions, BPR6-50, BPR6-75, BPR7-100, BPR8-50, BPWP6-75, BPWP6-100, BPWP7-100, and BPWP8-100 were found to have α-Amylase inhibition capacity. The protein extract’s α-amylase inhibitory activity profiles are shown in Fig. 7 and supplementary data Table 4.

### SDS PAGE results

All protein bands on SDS-PAGE were detected and analyzed through gel analyzer 19.1 as mentioned in the supplementary data in Fig. 1.

Among 9 protein fractions of *B. procumbens* roots, only 4 fractions showed prominent bands, in which fraction showed a common band of molecular weight 20 kDa, while the highest bands were observed in the range of 102 to 107 kDa. While in 9 protein fractions of the whole plant *B. procumbens*, only 6 samples showed protein bands. All these 4 fractions showed a common band of Molecular weight 46KDa. Sample BPWP7-100 showed 4 protein bands, in which the highest protein band was observed with a molecular weight of 118 KDa. Details are shown in the supplementary data Table 5.

### LC-MS/MS analysis

The gel bands were cut and subjected to sequencing by LC-MS/MS spectrometry. Proteins were identified by molecular weight. Proteins of various types were identified. No protein was novel. All of these proteins have already been isolated from other plant sources. But this study is the first to report the presence of the identified proteins in the plant *B. procumbens*. Further work is continued in our laboratory for the purification of these proteins that will help to reveal the novelty of the identified protein, if any. Initially, every band was cut and sent for LC-MS analysis because no information was available about the nature of the protein; hence, the approach for LC-MS analysis was non-specific because the study did not target any specific class of the protein. Although the interest of this study was to identify antidiabetic proteins, there have been no educated guesses to which specific protein band we should investigate. Hence, all protein bands were analyzed through LC-MS analysis and identified 20 proteins, and among them, 4 were antidiabetic.

The structures of all the proteins and enzymes were generated using the Alfafold software based on their sequences.

### Proteins having an antidiabetic effect

Some of the detected proteins are previously known for antidiabetic and antioxidant activity, which are given in Table 2.

Zerumbone synthase is involved in the synthesis of zerumbone^29^. Zerumbone synthase (Molecular weight 31 KDa) was detected in our plant *B. procumbens* for the first time in our study (Supplementary data Table 6 and sequence 1). A protein band with a molecular weight of 31 KDa was also observed in the SDS-PAGE result of sample BPR8-50. Zerumbone ((2E,6E,10E)-2,6,9,9-tetramethylcycloundeca-2,6,10-trien-1-one) was isolated from *Zingiber zerumbet* and found to have a potential broad use against different cancers, including leukemia, as well as viral infections^26,33,34^. Zerumbone also induces several pharmacological effects, including anti-inflammatory, antioxidant, and antibacterial^35,36^. *Z. zerumbet* also reduces the TG level in plasma and hepatic tissues^37^. Zerumbone is suggested to reduce hepatic steatosis (accumulation of fat in the liver) and improve insulin sensitivity in high-fat diet-induced diabetic mice^38^. Zerumbone confers its anti-cancer effects by causing great suppression of angiogenesis, proliferation, survival, invasion, and metastasis through the molecular modulation of different signaling pathways^39^.

γ-conglutin (Molecular weight 46 KDa) is one of the important proteins identified in our plant *B. procumbens* through LCMS/MS analysis (Supplementary data Table 7 and sequence 2). A band with the same molecular weight was also observed in SDS –PAGE result of the sample BPWP6-100. Sample BPWP6-100 also showed great α-amylase inhibitory effect having IC50 31.57 ± 2.5. γ-conglutin is known for its antidiabetic potential and capability to control glucose levels in the body. It has been formerly isolated from lupin seeds and also gained global attention due to its property of reducing the risk of type II diabetes development^66^. γ-conglutin proteins (PDB ID = 4PPH) are involved in insulin signaling, promote translocation of GLUT-4 receptors to the cell membrane, and regulate muscle-specific gene transcription, similar to insulin in vitro^40^. Primarily, γ-conglutin was known as a factor to contribute in secretion of insulin from beta cells, but in 2021, Mrunmai Tapadia isolated γ-conglutin from lupins, purified it up to 95%, and worked to find out its mechanism. According to his findings, γ-conglutin peptides are not involved in promoting insulin secretion in β-cells but elicit a unique insulin-mimetic action by activating insulin signaling pathways responsible for protein synthesis, glycogen, and glucose transport into myotubes. They also have the potential to modify the half-life of incretin hormones in circulation^41^.

Thioredoxin-dependent peroxidases were isolated by Aymaric Goyer in 2002 from *Chlamydomonas reinhardtii* and reported to play an antioxidant role^42^. It is also identified in *B. procumbens* during the present study. (Supplementary data 8 and sequence 3). Thioredoxin-dependent peroxiredoxin (Molecular weight 29) acts as a protector for β-cells. It detoxifies ROS, promotes β-cell function, and enhances survival in response to a variety of oxidative stressors^43^. Pancreatic β-cells are known as the primary source of insulin, which maintains glucose homeostasis. The main role of this cell is synthesis, storage, and secretion of insulin, which is carefully regulated in response to change in the body’s metabolic status^44^. Oxidative stress plays a crucial role in pancreatic β-cell damage, which leads to β-cells’ dysfunction and death in Diabetes (both type 1 and type 2).

Deepak Kadam isolated and characterized proteins from *Lepidium sativum*, including 7S protein of molecular weight 52 kDa, which was also detected in *B. procumbens* during the present study. (Supplementary data 9 and sequence 4). These proteins exhibit an antidiabetic effect by inhibiting alpha amylase and are considered antioxidants due to their ability to inhibit DPPH^45^. Basic 7S protein (PDB ID = 3ksc) is isolated from many plants and was primarily considered as a storage protein, but now many of its functions are known, such as antidiabetic, antioxidant, controls glucose level, blood pressure and plasma cholesterol level, and cancer cell and anti-bacterial^46^. Proteins isolated from *Momordica charantia* (*Mc*) seeds, including 7S protein (MW = 52 kDa), have the property of angiotensin-converting enzyme (ACE). ACS-inhibiting drugs are used for the treatment of myocardial infarction, hypertension, heart failure, and diabetic nephropathy. This protein also acts as an antioxidant^47^.

### Proteins/enzymes involved in glucose metabolism

Glucose is an important metabolite utilized by cells as a major energy source. It is not only the source of energy but also the substrate of cellular biosynthesis. The metabolic nonfunctioning of glucose homeostasis is the main cause of diabetes and diabetic patient mortality and morbidity^67^. Many proteins involved in glucose metabolism were detected in *B. procumbens* (Table 3).

Phosphopyruvate hydratase, also known as enolase, was first isolated from spinach^68^. It is an enzyme involved in glycolysis. It was detected in our plant *B. procumbens* for the first time in our study (Supplementary data 10 and sequence 5). Glycolysis is a metabolic pathway that breaks down glucose to produce energy. While phosphopyruvate hydratase itself does not have a direct antidiabetic role. It is indirectly related to diabetes through its involvement in regulating glucose metabolism and tyrosine nitration of alpha-enolase could contribute to an impaired glycolytic activity in diabetic cardiomyopathy. Meanwhile, to neutralize oxidative and nitrative stress in diabetes, the up-regulation of alpha-enolase expression could be a protective mechanism^48^.

The fructose-bisphosphate aldolase was primarily isolated and characterized from spinach^69^ and later isolated from *Sesuvium portulacastrum*^70^. Our findings confirmed the presence of this enzyme in *B. procumbens* (Supplementary data 11 and sequence 6). It is another glycolytic enzyme that catalyzes the conversion of fructose 1-6-diphosphate to dihydroxy-acetone phosphate and glyceraldehyde 3-phosphate. Increased ALDOB gene expression in human islets is affiliated with lower insulin secretion^49^.

Glucan was earlier extracted and isolated from grains, mainly from oats and barley^71^. α-Glucan was also identified in our plant *B. procumbens* (Supplementary data 12 and sequence 7). α-Glucan phosphorylases (α-GPs) catalyze the reversible phosphorolysis of α-1,4-linked polysaccharides such as starch, glycogens, and maltodextrins, and it is, therefore, playing the main role in the usage of storage polysaccharides.

Aldo keto reductase (AKR) isolated from *Arabidopsis thaliana*, named 4C8, 4C9, 4C10, and 4C11, detoxifies toxic aldehydes and ketones produced during stress^51^. This stress managing enzyme was also identified in our plant *B. procumbens* (Supplementary data 13 and sequence 8). MsALR is an aldo-keto reductase isolated from *Medicago sativa* that detoxifies lipid peroxidation-derived reactive aldehydes^52^. Vr-ERE/VrALR from *Vigna radiate* reduce fungal toxin eutypine and help to neutralize drought stress-induced reactive aldehyde^53^. *Oryza sativa ssp.*. Indica has AKR named AKR4C14 (OsI-04428), which is responsible for metabolization of sugars and reactive aldehydes, including methylglyoxal, glutaraldehyde, and Trans-2-hexenal^54^. PpAKR1 from *Prunus persica is* responsible for cold, salt, and oxidative stress inducible^55^.

UTP-glucose-1-phosphate uridylyltransferase, also known as UGP, was identified in our plant B. procumbens during our study (Supplementary data 14 and sequence 9). It is an enzyme that plays a crucial role in glycogen biosynthesis, which is a process closely related to glucose metabolism. The balance between glycogen storage (glycogenesis) and glycogen breakdown (glycogenolysis) must be regulated. UGP’s role in glycogen synthesis contributes to promoting glycogen storage, which can help to lower and stabilize blood glucose levels when necessary. In plant leaves, UTP—UTP-glucose-1-phosphate uridylyltransferase is the main enzyme involved in the biosynthetic pathway of sucrose, providing Uridine diphosphate glucose to Sucrose phosphate synthase that combines fructose 6 phosphate and UDP glucose into sucrose 6 phosphate^56^.

Vicilin (PDB ID = 2phl) isolated from *Phaseolus vulgaris*^72^ (common beans) resembled the vicilin protein present in *B. procumbens*. (Supplementary data 15 and sequence 10). Vicilin serves as a storage source of amino acids and nutrients for the developing plant embryo. It has no direct antidiabetic effect. However, vicilin-rich food can provide proteins, fibers, and nutrients that can regulate the blood sugar level of blood^57^.

### Miscellaneous proteins

Some other proteins of diverse function were detected through LC/MS. The detail is mentioned in Table 4.

Serene protease, identified in our plant *B. procumbens* (Supplementary data 16 and sequence 11), was primarily isolated from *Wrightia tinctoria* and is known for its anti-microbial role^73^. This enzyme is responsible for cleaving peptide bonds in proteins. At the active site of the enzyme, the serine protease acts as a nucleophilic amino acid^58^. Prunifoline, a new protease, was purified from the latex of *Euphorbia prunifolia*, which has great importance in the food and biotechnology industry.

Amine oxidase is an important enzyme that was detected in our plant *B. procumbens* (Supplementary data 17 and sequence 12). Formerly, it was isolated and characterized from many plant sources like pea seedlings^74^* Pisum sativum*^75^ and the *Fabaceae* plant^76^. It activates oxidative deamination reactions, producing an aldehyde and ammonia. In eukaryotes, they have an extensive range of functions, including cell differentiation and growth, wound healing, detoxification, and cell signaling^59^.

Legumin (Supplementary data 18 and sequence 13) was previously isolated mainly from beans and pea seeds^77^. It is known as vegetable milk because it is similar to the casein present in mammalian milk. The primary function of legumin is the storage of angiosperms and gymnosperms^38^.

The α -amylase (PDB ID = 1amy) was primarily isolated from barley^78^ and present study is the first to report this enzyme in plant *B. procumbens* (Supplementary data 19 and sequence 14) The inhibition of α -glucosidase and amylase (α- and β-, enzymes involved in the digestion of carbohydrates, can remarkably decrease the after meal increase of blood glucose and therefore can be an strategy for the control of blood glucose level in type 2 diabetic and borderline patients^60^.

Glucosidases (α-and β-) detected in our plant *B. procumbens* (Supplementary data 20 and sequence 15) initiate the hydrolysis of polysaccharides to simple sugars, which increase glucose levels in blood. The principal role of β-glucosidase in cellulolytic microorganisms is to catalyze the hydrolysis of cellobiose and cello-oligosaccharides, producing glucose during bioconversion^61^.

Glyceraldehyde 3-phosphate dehydrogenase was first identified in *Zea mays* L leaves^79^ and then it was reported in *Arabidopsis thaliana*^80^ but during current study for the first time it is identified in *B. procumbens* (Supplementary data 21 and sequence 16) Glyceraldehyde 3-phosphate dehydrogenase (GAPDH) plays a key regulatory function in glucose oxidation by mediating fluxes through glycolysis or the pentose phosphate pathway (PPP) in an oxidative stress–dependent fashion^62^.

Lactoylglutathione lyase (Supplementary data 22 and sequence 17) plays an important role in the survival of *Salmonella* in the nutrient-rich environment due to its ability to detoxify methylglyoxal detoxification^63^. Lacto glutathione lyase is also known as glyoxalase (GLO1). Some studies suggest that reduced GLO1 activity could result in the accumulation of methyl glyoxal, which, in turn, may impair insulin signaling and contribute to insulin resistance.

Ascorbate oxidase was identified in tobacco^81^ and also isolated from *Cucurbita pepo* var. melopepo^82^ (PDB ID = 1aoz), which resembles a protein present in *B. procumbens* (Supplementary data 23 and sequence 18). L-ascorbate oxidase is an enzyme involved in the biosynthesis of Vitamin C. Vitamin C is a well-known antioxidant that can reduce oxidative stress which is a factor of diabetes. Vitamin C is an electron donor, and this property accounts for all its known functions. As an electron donor, vitamin C is a potent water-soluble antioxidant in humans. Vitamin C is a strong antioxidant^64^.

Cysteine proteinases (Supplementary data 24 and sequence 19), also referred to as thiol proteases, play an essential role in plant growth and development but also in senescence and programmed cell death, in the accumulation of storage proteins such as in seeds, and in storage protein mobilization. Papain-like cysteine proteases (PLCPs) play crucial roles in plant-pathogen/pest interactions^65^.

## Conclusion

The α-glucosidase and α-amylase inhibitory potential of *B. procumbens*’s partially purified protein fractions was found better than the standard antidiabetic drugs tested during present study; hence, these active protein fractions could be a better alternative approach to cure diabetes rather than the synthetic drugs available in the market. Results of LC-MS/MS analysis revealed that this plant contains proteins with many beneficial biological effects, including antidiabetic, anticancer, storage proteins, antitumor, and antiallergic roles. While some of the identified proteins participate in various essential biological processes such as the Krebs’ cycle, and glucose metabolism, etc.

## Future horizon

Although we have been able to detect many valuable proteins with diverse biological roles in *B. procumbens*, these proteins need to be purified, crystallized, and further analyzed for detailed functional evaluation. To confirm the antidiabetic potential, it is recommended that the purified protein fraction must be tested, in vivo, in diabetic mice models. Hence, the research work is in progress in our lab to purify the antidiabetic proteins from the partially purified protein fractions, and structure elucidation and functional characterization of the purified proteins via wide range of bioassays including in vivo testing in diabetic mice models. It is suggested that the folk use of this conventional antidiabetic herb must be translated into a more medicinally acceptable therapeutic strategy involving the use of antidiabetic proteins/peptides purified from *B. procumbens.*

## Supplementary Information

Below is the link to the electronic supplementary material.

Supplementary Material 1

## Data Availability

All the data analyzed or studied during the study are included in this article and the attached supplementary file.
