# Supplementary material for: Analysis of proteins and peptides to investigate the molecular signatures of a conventional antidiabetic herb, Boerhavia procumbens Banks ex Roxb. (Nyctaginaceae)
Source: Sci Rep. 2025 Dec 31;16:31. doi: 10.1038/s41598-025-16474-8 (PMC12764989; doi:10.1038/s41598-025-16474-8)
Supplement: Supplementary file 1 — Supplementary Material 1 [file 41598_2025_16474_MOESM1_ESM.docx]

**Analysis of Proteins and Peptides to Investigate the Molecular Signatures of a Conventional Anti-Diabetic Herb, *Boerhavia procumbens* Banks ex Roxb. *(Nyctaginaceae),* from Cholistan Desert, Pakistan**

Ghazala Hassan,^1^ Shazia Anjum,*^1,3^ Samina Ejaz,*^2^ Muhammad Ashraf,^1^Ayesha Momen^1^

^1^Department of Chemistry, The Islamia University of Bahawalpur, Bahawalpur-63100, Pakistan

^2^Department of Biochemistry, The Islamia University of Bahawalpur, Bahawalpur-63100, Pakistan

^3^International Center for Chemical and Biological Sciences, H.E.J. Research Institute of Chemistry, Karachi University Karachi-75270, Pakistan

**Supplementary data**

**Tables**

**Table 1:** Composition of gel solutions

| Gel type | Water  (mL) | 30% acrylamide  (mL) | 1.5 M  Tris HCL pH 8.8 (mL) | 0.5 Tris HCl, pH 6.8 (mL) | 10% SDS (µL) | 10% APS (µL) | Temed |
| --- | --- | --- | --- | --- | --- | --- | --- |
| Sealing | 5.13 | 3.14 | 1.3 | N/A | 50 | 100 | 10 |
| Resolving | 9.4 | 13.1 | 10 | 00 | 400 | 200 | 20 |
| Stacking | 6.7 | 1.75 | 00 | 1.25 | 100 | 100 | 10 |

**Table 2:** Quantity of protein (µg/µl) in extracts

| **Sr No.** | **Sample code** | **pH** | **Precipitatio of Protein%** | **Quantity of protein content (µg/µl)** |
| --- | --- | --- | --- | --- |
| **Roots** | | | | |
| 1 | BPR6-50 | 6 | 50 | 0.364314 |
| 2 | BPR6-75 |  | 75 | 0.002876 |
| 3 | BPR6-100 |  | 100 | 0.044706 |
| 4 | BPR7-50 | 7 | 50 | 0.341438 |
| 5 | BPR7-75 |  | 75 | 0.73098 |
| 6 | BPR7-100 |  | 100 | 0.053856 |
| 7 | BPR8-50 | 8 | 50 | 0.475425 |
| 8 | BPR8-75 |  | 75 | 0.30549 |
| 9 | BPR8-100 |  | 100 | 0.155817 |
| **Whole plant** | | | | |
| 10 | BPW6-50 | 6 | 50 | 0.366928 |
| 11 | BPW6-75 |  | 75 | 0.07085 |
| 12 | BPW6-100 |  | 100 | 0.291111 |
| 13 | BPW7-50 | 7 | 50 | 0.384933 |
| 14 | BPW7-75 |  | 75 | 0.104183 |
| 15 | BPW7-100 |  | 100 | 0.000915 |
| 16 | BPW8-50 | 8 | 50 | 0.39634 |
| 17 | BPW8-75 |  | 75 | 0.534902 |
| 18 | BPW8-100 |  | 100 | 0.07085 |

**Table 3** α-Glucosidase inhibitory profiles of crude and Protein extracts. (Mean ± SEM, n=3).

| **Sr. No.** | **Comp Code** | **Inhibition (%) at 0.5 mg/mL** | **IC_50_ (µg/mL)** |
| --- | --- | --- | --- |
| 1. | BPR6-50 | 32.5 ±1.34 | - |
| 2. | BPR6-75 | 21.9±1.23 | - |
| 3. | BPR6-100 | 24.6±1.32 | - |
| 4. | BPR7-50 | 36.4±1.43 | - |
| 5. | BPR7-75 | 27.8±1.38 | - |
| 6. | BPR7-100 | 38.5±1.27 | - |
| 7. | BPR8-50 | 41.6±1.25 | - |
| 8. | BPR8-75 | 25.8±1.32 | - |
| 9. | BPR8-100 | 47.9±1.42 | - |
| 10. | BPWP6-50 | 34.7±1.39 | - |
| 11. | BPWP6-75 | 38.5±1.51 | - |
| 12. | BPWP6-100 | 62.9±1.42 | 137.8±1.2 |
| 13. | BPWP7-50 | 46.5±1.35 | - |
| 14. | BPWP7-75 | 41.6±1.53 | - |
| 15. | BPWP7-100 | 56.8±1.42 | 352.3±1.2 |
| 16. | BPWP8-50 | 43.6±1.37 | - |
| 17. | BPWP8-75 | 48.7±1.36 | - |
| 18. | BPWP-100 | 72.9±1.53 | 187.4±1.3 |
| 19. | BPR-06 (crude) | 65.7±1.47 | 215.9±1.4 |
| 20. | BPR-07 (crude) | 85.9±1.58 | 24.8±1.5 |
| 21. | BPR-08 (crude) | 67.4±1.53 | 152.4±1.3 |
| 22. | BPWP-06 (crude) | 62.5±1.59 | 157.3±1.4 |
| 23. | BPWP-07 (crude) | 59.2±1.48 | 268.4±1.3 |
| 24. | BPWP-08 (crude) | 64.7±1.57 | 164.7±1.3 |
| 26. | Standard acarbose | 56.8±1.37 | 372.41±1.25 |

**Table 4:** α-amylase inhibitory profiles of crude and Protein extracts. (Mean ± SEM, n=3).

| **Sr. No.** | **Comp Code** | **Inhibition (%) at 0.5 mg/mL** | **IC_50_ (µg/mL)** |
| --- | --- | --- | --- |
| 1. | BPR6-50 | 71±1.43 | 39.05±2.6 |
| 2. | BPR6-75 | 57±1.22 | 85.55±4 |
| 3. | BPR6-100 | 37±1.23 | - |
| 4. | BPR7-50 | 12±1.34 | - |
| 5. | BPR7-75 | 27±1.38 | - |
| 6. | BPR7-100 | 58.92±1.27 | 48.09±2.7 |
| 7. | BPR8-50 | 62±1.25 | 41.22±3.5 |
| 8. | BPR8-75 | 47±1.42 | - |
| 9. | BPR8-100 | 12±1.32 | - |
| 10. | BPWP6-50 | 28±1.29 | - |
| 11. | BPWP6-75 | 38.5±1.51 | 59.82±3.5 |
| 12. | BPWP6-100 | 62.9±1.42 | 31.57±2.5 |
| 13. | BPWP7-50 | 37.5±1.55 | - |
| 14. | BPWP7-75 | 45.6±1.35 | - |
| 15. | BPWP7-100 | 84±1.42 | 46.52±3.2 |
| 16. | BPWP8-50 | 34.6±1.37 | - |
| 17. | BPWP8-75 | 38.7±1.36 | - |
| 18. | BPWP-100 | 72.9±1.53 | 24.24±1.8 |
| 19. | BPR-06 (crude) | 60±1.47 | 18.68±2.7 |
| 20. | BPR-07 (crude) | 68.15±1.58 | 6.954±1.7 |
| 21. | BPR-08 (crude) | 79±1.53 | 10.08±2.5 |
| 22. | BPWP-06 (crude) | 62.5±1.59 | 7.68±1.5 |
| 23. | BPWP-07 (crude) | 62.2±1.48 | 26.11±4 |
| 24. | BPWP-08 (crude) | 81.7±1.57 | 10.59±1.3 |
| 25 | Std Acarbose | 60±1.22 | 18.31 ± 4 |
| 26 | Std Metformin | 56±1.25 | 21.92±3 |
| 27 | Std Empagliflozin | 52±1.46 | 155±2.6 |

**Table 5:** SDS PAGE detected bands with molecular weight and band volume of protein sample

| **Sr. No.** | **Plant part** | **Sample** | **No. of protein bands on sample** | **Mol. weight of band** | **Band volume** |
| --- | --- | --- | --- | --- | --- |
| 1 | Roots | BPR6-50 | 3 | 102 | 126 |
|  |  |  |  | 44 | 25 |
|  |  |  |  | 20 | 815 |
| 2 |  | BPR6-75 | 3 | 105 | 234 |
|  |  |  |  | 94 | 353 |
|  |  |  |  | 20 | 1087 |
| 3 |  | BPR7-100 | 2 | 107 | 230 |
|  |  |  |  | 20 | 1326 |
| 4 |  | BPR8-50 | 3 | 107 | 174 |
|  |  |  |  | 31 | 43 |
|  |  |  |  | 20 | 1371 |
| 5 | Whole plant | BPWP6-75 | 4 | 105 | 169 |
|  |  |  |  | 43 | 18 |
|  |  |  |  | 21 | 1359 |
|  |  |  |  | 20 | 51 |
| 6 |  | BPWP6-100 | 3 | 46 | 70 |
|  |  |  |  | 22 | 279 |
|  |  |  |  | 20 | 1609 |
| 7 |  | BPWP7-100 | 4 | 118 | 340 |
|  |  |  |  | 46 | 51 |
|  |  |  |  | 28 | 109 |
|  |  |  |  | 20 | 1308 |
| 8 |  | BPWP8-100 | 3 | 114 | 387 |
|  |  |  |  | 47 | 53 |
|  |  |  |  | 20 | 984 |
| 9 |  | BPWP8-50 | 2 | 46 | 27 |
|  |  |  |  | 20 | 773 |
| 10 |  | BPWP8-75 | 3 | 110 | 417 |
|  |  |  |  | 45 | 139 |
|  |  |  |  | 20 | 242 |

Sequence no 1 (Zerumbone synthase)

| [>tr\|A0A1U8M7S6\|A0A1U8M7S6_GOSHI Zerumbone synthase-like OS=Gossypium hirsutum OX=3635 GN=LOC107934860 PE=4 SV=1](http://www.uniprot.org/uniprot/?query=A0A1U8M7S6_GOSHI) |
| --- |
| MLRSLARHFK FATNDVCSKK LRFYSAQPDG SATSRLQGKV ALITGGASGL GKATAIEFVK NGAHVIIADI DPQVGHEAAN ALGPSARFVQ CDVTMESQVA  EAVQVAMEQH GKLDIMFNNA GITGQAFPPS IAELDLEEFD RVMRINVRGM VAGIKHAARV MVPVGSGSIL CTSSISGLMG GLGPHPYTIA KFTIPGIVKS  VASELCRTGV RINCISPAPI PTPMVIRQIA EIYQGIPKEK VVEIINGVGE LKGAKCEEID VAKAALYLAS DEAKYVTGHN LVVDGGFTSF KNLTFPSLSS |

| Legend | | |
| --- | --- | --- |
| THISREFERE NCE | UNMATCHED | OTHERREFER ENCES |

| \| 31703.44 Da \| Protein Coverage \| \| \| --- \| --- \| --- \| \| 300 AA \| 8 AA \| **2.67 %** \| |
| --- | --- | --- | --- | --- | --- | --- |

Data table 6

| \| **Sequence** \| \| --- \| \| FTIPGIVK \| |
| --- | --- | --- |
| **Predicted Fragmentation Pattern**   \| **Seq** \| **#** \| **b: Δ Error** \| **b** \| **y** \| **y: Δ Error** \| **+1** \| \| --- \| --- \| --- \| --- \| --- \| --- \| --- \| \| **F** \| **1** \| **---** \| [148.076](https://tmsf.med.harvard.edu/core/www/modules/sdig/index.php?run_id=92259&search_id=93773&scans_id=92255&peptide_id=2831&scanf=11955&charge=2&value=K.FTIPGIVK.S&mz_l=48.076&mz_h=248.076) \| --- \| **---** \| **8** \| \| **T** \| **2** \| **-0.433** \| [**249.123**](https://tmsf.med.harvard.edu/core/www/modules/sdig/index.php?run_id=92259&search_id=93773&scans_id=92255&peptide_id=2831&scanf=11955&charge=2&value=K.FTIPGIVK.S&mz_l=149.123&mz_h=349.123) \| [**727.471**](https://tmsf.med.harvard.edu/core/www/modules/sdig/index.php?run_id=92259&search_id=93773&scans_id=92255&peptide_id=2831&scanf=11955&charge=2&value=K.FTIPGIVK.S&mz_l=627.471&mz_h=827.471) \| **-1.426** \| **7** \| \| **I** \| **3** \| **---** \| [362.207](https://tmsf.med.harvard.edu/core/www/modules/sdig/index.php?run_id=92259&search_id=93773&scans_id=92255&peptide_id=2831&scanf=11955&charge=2&value=K.FTIPGIVK.S&mz_l=262.207&mz_h=462.207) \| [**626.424**](https://tmsf.med.harvard.edu/core/www/modules/sdig/index.php?run_id=92259&search_id=93773&scans_id=92255&peptide_id=2831&scanf=11955&charge=2&value=K.FTIPGIVK.S&mz_l=526.424&mz_h=726.424) \| **-0.958** \| **6** \| \| **P** \| **4** \| **---** \| [459.260](https://tmsf.med.harvard.edu/core/www/modules/sdig/index.php?run_id=92259&search_id=93773&scans_id=92255&peptide_id=2831&scanf=11955&charge=2&value=K.FTIPGIVK.S&mz_l=359.26&mz_h=559.26) \| [**513.340**](https://tmsf.med.harvard.edu/core/www/modules/sdig/index.php?run_id=92259&search_id=93773&scans_id=92255&peptide_id=2831&scanf=11955&charge=2&value=K.FTIPGIVK.S&mz_l=413.34&mz_h=613.34) \| **-0.896** \| **5** \| \| **G** \| **5** \| **---** \| [516.282](https://tmsf.med.harvard.edu/core/www/modules/sdig/index.php?run_id=92259&search_id=93773&scans_id=92255&peptide_id=2831&scanf=11955&charge=2&value=K.FTIPGIVK.S&mz_l=416.282&mz_h=616.282) \| [**416.287**](https://tmsf.med.harvard.edu/core/www/modules/sdig/index.php?run_id=92259&search_id=93773&scans_id=92255&peptide_id=2831&scanf=11955&charge=2&value=K.FTIPGIVK.S&mz_l=316.287&mz_h=516.287) \| **-0.887** \| **4** \| \| **I** \| **6** \| **---** \| [629.366](https://tmsf.med.harvard.edu/core/www/modules/sdig/index.php?run_id=92259&search_id=93773&scans_id=92255&peptide_id=2831&scanf=11955&charge=2&value=K.FTIPGIVK.S&mz_l=529.366&mz_h=729.366) \| [359.265](https://tmsf.med.harvard.edu/core/www/modules/sdig/index.php?run_id=92259&search_id=93773&scans_id=92255&peptide_id=2831&scanf=11955&charge=2&value=K.FTIPGIVK.S&mz_l=259.265&mz_h=459.265) \| **---** \| **3** \| \| **V** \| **7** \| **---** \| [728.434](https://tmsf.med.harvard.edu/core/www/modules/sdig/index.php?run_id=92259&search_id=93773&scans_id=92255&peptide_id=2831&scanf=11955&charge=2&value=K.FTIPGIVK.S&mz_l=628.434&mz_h=828.434) \| [246.181](https://tmsf.med.harvard.edu/core/www/modules/sdig/index.php?run_id=92259&search_id=93773&scans_id=92255&peptide_id=2831&scanf=11955&charge=2&value=K.FTIPGIVK.S&mz_l=146.181&mz_h=346.181) \| **---** \| **2** \| \| **K** \| **8** \| **---** \| --- \| [**147.113**](https://tmsf.med.harvard.edu/core/www/modules/sdig/index.php?run_id=92259&search_id=93773&scans_id=92255&peptide_id=2831&scanf=11955&charge=2&value=K.FTIPGIVK.S&mz_l=47.113&mz_h=247.113) \| **-0.701** \| **1** \| |

Supplementary sequence no 2

| [>sp\|P82952\|CONG1_PRUDU Gamma conglutin 1 OS=Prunus dulcis OX=3755 GN=Cgamma1 PE=1 SV=2](http://www.uniprot.org/uniprot/?query=CONG1_PRUDU) |
| --- |
| MASFLHNFLL FFCSLSLIIL TSSATKSQTH VPIRPNKLVL KVQKDRATNL HVVQIHKRTP LVQFPFVIDL TGRFLSVNCE NQYTSSTYKA PVCHSSQCAR  ANSHTCRTCS SSKTRPGCHT NACGLLTTNP VTQQSAQGEL AEDVLKIPST QGSSPGPMVT YPHFLFACAP SNILQKGLPK NVQGVAGLGH SPISLPYQLA  SHFGFPPKFA VCLTSSPGKN GAVFFGEGPY FMKPGIDVSR QLTYAPFTIG QQGEYYINVQ SFKINNAMLP SIPKGGFGGA MISTTTPYTT LQTPIFRALN  QLFMNQLRGV PHVKPVAPFG ACFDANRIPT SKMGPTVPSI DLVLDNKKNI MWRIFGANAM IQPRPGVMCL AFVDGGMRPK APIVIGTQQL EDNLLQFDLM  NSRLGFSSSL LFRRTNCANF NFGTSSTNTD P |

| Legend | | |
| --- | --- | --- |
| THISREFERE NCE | UNMATCHED | OTHERREFER ENCES |

| \| 46914.84 Da \| Protein Coverage \| \| \| --- \| --- \| --- \| \| 431 AA \| 15 AA \| **3.48 %** \| |
| --- | --- | --- | --- | --- | --- | --- |

**Data table # 7**

| \| **Sequence** \| \| --- \| \| TPLVQFPFVIDLTGR \| |
| --- | --- | --- |
| **Predicted Fragmentation Pattern**   \| **Seq** \| **#** \| **b: Δ Error** \| **b** \| **y** \| **y: Δ Error** \| **+1** \| \| --- \| --- \| --- \| --- \| --- \| --- \| --- \| \| **T** \| **1** \| **---** \| [102.055](https://tmsf.med.harvard.edu/core/www/modules/sdig/index.php?value=13/28&pvkey=&run_id=92258&search_id=93772&scans_id=92254&peptide_id=9060&charge=2&scanf=18027&mz_l=2.055&mz_h=202.055) \| --- \| **---** \| **15** \| \| **P** \| **2** \| **-0.799** \| [**199.108**](https://tmsf.med.harvard.edu/core/www/modules/sdig/index.php?value=13/28&pvkey=&run_id=92258&search_id=93772&scans_id=92254&peptide_id=9060&charge=2&scanf=18027&mz_l=99.108&mz_h=299.108) \| [1601.905](https://tmsf.med.harvard.edu/core/www/modules/sdig/index.php?value=13/28&pvkey=&run_id=92258&search_id=93772&scans_id=92254&peptide_id=9060&charge=2&scanf=18027&mz_l=1501.905&mz_h=1701.905) \| **---** \| **14** \| \| **L** \| **3** \| **-0.814** \| [**312.192**](https://tmsf.med.harvard.edu/core/www/modules/sdig/index.php?value=13/28&pvkey=&run_id=92258&search_id=93772&scans_id=92254&peptide_id=9060&charge=2&scanf=18027&mz_l=212.192&mz_h=412.192) \| [**1504.852**](https://tmsf.med.harvard.edu/core/www/modules/sdig/index.php?value=13/28&pvkey=&run_id=92258&search_id=93772&scans_id=92254&peptide_id=9060&charge=2&scanf=18027&mz_l=1404.852&mz_h=1604.852) \| **0.208** \| **13** \| \| **V** \| **4** \| **-1.789** \| [**411.260**](https://tmsf.med.harvard.edu/core/www/modules/sdig/index.php?value=13/28&pvkey=&run_id=92258&search_id=93772&scans_id=92254&peptide_id=9060&charge=2&scanf=18027&mz_l=311.26&mz_h=511.26) \| [**1391.768**](https://tmsf.med.harvard.edu/core/www/modules/sdig/index.php?value=13/28&pvkey=&run_id=92258&search_id=93772&scans_id=92254&peptide_id=9060&charge=2&scanf=18027&mz_l=1291.768&mz_h=1491.768) \| **-1.034** \| **12** \| \| **Q** \| **5** \| **-1.447** \| [**539.319**](https://tmsf.med.harvard.edu/core/www/modules/sdig/index.php?value=13/28&pvkey=&run_id=92258&search_id=93772&scans_id=92254&peptide_id=9060&charge=2&scanf=18027&mz_l=439.319&mz_h=639.319) \| [**1292.700**](https://tmsf.med.harvard.edu/core/www/modules/sdig/index.php?value=13/28&pvkey=&run_id=92258&search_id=93772&scans_id=92254&peptide_id=9060&charge=2&scanf=18027&mz_l=1192.7&mz_h=1392.7) \| **-1.166** \| **11** \| \| **F** \| **6** \| **1.451** \| [**686.387**](https://tmsf.med.harvard.edu/core/www/modules/sdig/index.php?value=13/28&pvkey=&run_id=92258&search_id=93772&scans_id=92254&peptide_id=9060&charge=2&scanf=18027&mz_l=586.387&mz_h=786.387) \| [**1164.641**](https://tmsf.med.harvard.edu/core/www/modules/sdig/index.php?value=13/28&pvkey=&run_id=92258&search_id=93772&scans_id=92254&peptide_id=9060&charge=2&scanf=18027&mz_l=1064.641&mz_h=1264.641) \| **-1.308** \| **10** \| \| **P** \| **7** \| **---** \| [783.440](https://tmsf.med.harvard.edu/core/www/modules/sdig/index.php?value=13/28&pvkey=&run_id=92258&search_id=93772&scans_id=92254&peptide_id=9060&charge=2&scanf=18027&mz_l=683.44&mz_h=883.44) \| [**1017.573**](https://tmsf.med.harvard.edu/core/www/modules/sdig/index.php?value=13/28&pvkey=&run_id=92258&search_id=93772&scans_id=92254&peptide_id=9060&charge=2&scanf=18027&mz_l=917.573&mz_h=1117.573) \| **-1.083** \| **9** \| \| **F** \| **8** \| **---** \| [930.508](https://tmsf.med.harvard.edu/core/www/modules/sdig/index.php?value=13/28&pvkey=&run_id=92258&search_id=93772&scans_id=92254&peptide_id=9060&charge=2&scanf=18027&mz_l=830.508&mz_h=1030.508) \| [**920.520**](https://tmsf.med.harvard.edu/core/www/modules/sdig/index.php?value=13/28&pvkey=&run_id=92258&search_id=93772&scans_id=92254&peptide_id=9060&charge=2&scanf=18027&mz_l=820.52&mz_h=1020.52) \| **5.266** \| **8** \| \| **V** \| **9** \| **-8.297** \| [**1029.577**](https://tmsf.med.harvard.edu/core/www/modules/sdig/index.php?value=13/28&pvkey=&run_id=92258&search_id=93772&scans_id=92254&peptide_id=9060&charge=2&scanf=18027&mz_l=929.577&mz_h=1129.577) \| [**773.452**](https://tmsf.med.harvard.edu/core/www/modules/sdig/index.php?value=13/28&pvkey=&run_id=92258&search_id=93772&scans_id=92254&peptide_id=9060&charge=2&scanf=18027&mz_l=673.452&mz_h=873.452) \| **6.733** \| **7** \| \| **I** \| **10** \| **---** \| [1142.661](https://tmsf.med.harvard.edu/core/www/modules/sdig/index.php?value=13/28&pvkey=&run_id=92258&search_id=93772&scans_id=92254&peptide_id=9060&charge=2&scanf=18027&mz_l=1042.661&mz_h=1242.661) \| [**674.383**](https://tmsf.med.harvard.edu/core/www/modules/sdig/index.php?value=13/28&pvkey=&run_id=92258&search_id=93772&scans_id=92254&peptide_id=9060&charge=2&scanf=18027&mz_l=574.383&mz_h=774.383) \| **-13.376** \| **6** \| \| **D** \| **11** \| **-7.701** \| [**1257.688**](https://tmsf.med.harvard.edu/core/www/modules/sdig/index.php?value=13/28&pvkey=&run_id=92258&search_id=93772&scans_id=92254&peptide_id=9060&charge=2&scanf=18027&mz_l=1157.688&mz_h=1357.688) \| [**561.299**](https://tmsf.med.harvard.edu/core/www/modules/sdig/index.php?value=13/28&pvkey=&run_id=92258&search_id=93772&scans_id=92254&peptide_id=9060&charge=2&scanf=18027&mz_l=461.299&mz_h=661.299) \| **2.014** \| **5** \| \| **L** \| **12** \| **---** \| [1370.772](https://tmsf.med.harvard.edu/core/www/modules/sdig/index.php?value=13/28&pvkey=&run_id=92258&search_id=93772&scans_id=92254&peptide_id=9060&charge=2&scanf=18027&mz_l=1270.772&mz_h=1470.772) \| [**446.272**](https://tmsf.med.harvard.edu/core/www/modules/sdig/index.php?value=13/28&pvkey=&run_id=92258&search_id=93772&scans_id=92254&peptide_id=9060&charge=2&scanf=18027&mz_l=346.272&mz_h=546.272) \| **-1.648** \| **4** \| \| **T** \| **13** \| **---** \| [1471.820](https://tmsf.med.harvard.edu/core/www/modules/sdig/index.php?value=13/28&pvkey=&run_id=92258&search_id=93772&scans_id=92254&peptide_id=9060&charge=2&scanf=18027&mz_l=1371.82&mz_h=1571.82) \| [**333.188**](https://tmsf.med.harvard.edu/core/www/modules/sdig/index.php?value=13/28&pvkey=&run_id=92258&search_id=93772&scans_id=92254&peptide_id=9060&charge=2&scanf=18027&mz_l=233.188&mz_h=433.188) \| **-0.136** \| **3** \| \| **G** \| **14** \| **---** \| [1528.841](https://tmsf.med.harvard.edu/core/www/modules/sdig/index.php?value=13/28&pvkey=&run_id=92258&search_id=93772&scans_id=92254&peptide_id=9060&charge=2&scanf=18027&mz_l=1428.841&mz_h=1628.841) \| [**232.140**](https://tmsf.med.harvard.edu/core/www/modules/sdig/index.php?value=13/28&pvkey=&run_id=92258&search_id=93772&scans_id=92254&peptide_id=9060&charge=2&scanf=18027&mz_l=132.14&mz_h=332.14) \| **-0.876** \| **2** \| \| **R** \| **15** \| **---** \| --- \| [**175.119**](https://tmsf.med.harvard.edu/core/www/modules/sdig/index.php?value=13/28&pvkey=&run_id=92258&search_id=93772&scans_id=92254&peptide_id=9060&charge=2&scanf=18027&mz_l=75.119&mz_h=275.119) \| **-1.104** \| **1** \| |

**Sequence # 3**

| [>tr\|A0A1U8IM99\|A0A1U8IM99_GOSHI thioredoxin-dependent peroxiredoxin OS=Gossypium hirsutum OX=3635 GN=LOC107898212 PE=4 SV=1](http://www.uniprot.org/uniprot/?query=A0A1U8IM99_GOSHI) |
| --- |
| MACSATSSAA ALISSKPRAF SSNSKPFSQS LSLPNPFTGL PAPLLSRPAS FSLSRTSPSR KSFVVKATSE LPLVGNPAPD FEAEAVFDQE FIKVKLSEYI  GKKYVILFFY PLDFTFVCPT EITAFSDRYE EFEKLNTEIL GVSIDSVFSH LAWVQTDRKS GGLGDLKYPL ISDVTKTIAK AYGVLIPDQG IALRGLFIID  KEGIIQHSTI NNLAIGRSVD ETMRTLQALQ YVQENPSCLD SKGVASSKDP ITNGIVPIVL GKKGGISLRP N |

| Legend | | |
| --- | --- | --- |
| THISREFERE NCE | UNMATCHED | OTHERREFER ENCES |

| \| 29368.41 Da \| Protein Coverage \| \| \| --- \| --- \| --- \| \| 271 AA \| 49 AA \| **18.08 %** \| |
| --- | --- | --- | --- | --- | --- | --- |

**Data table # 8**

| \| **Sequence** \| \| --- \| \| EGIIQHSTINNLAIGR \| |
| --- | --- | --- |
| **Predicted Fragmentation Pattern**  **+1**   \| **Seq** \| **#** \| **b: Δ Error** \| **b** \| **y** \| **y: Δ Error** \| **+1** \| \| --- \| --- \| --- \| --- \| --- \| --- \| --- \| \| **E** \| **1** \| **0.326** \| [**130.050**](https://tmsf.med.harvard.edu/core/www/modules/sdig/index.php?value=11/60&pvkey=&run_id=92259&search_id=93773&scans_id=92255&peptide_id=1562&charge=3&scanf=10708&mz_l=30.05&mz_h=230.05) \| --- \| **---** \| **16** \| \| **G** \| **2** \| **-0.560** \| [**187.071**](https://tmsf.med.harvard.edu/core/www/modules/sdig/index.php?value=11/60&pvkey=&run_id=92259&search_id=93773&scans_id=92255&peptide_id=1562&charge=3&scanf=10708&mz_l=87.071&mz_h=287.071) \| [1606.902](https://tmsf.med.harvard.edu/core/www/modules/sdig/index.php?value=11/60&pvkey=&run_id=92259&search_id=93773&scans_id=92255&peptide_id=1562&charge=3&scanf=10708&mz_l=1506.902&mz_h=1706.902) \| **---** \| **15** \| \| **I** \| **3** \| **-1.733** \| [**300.155**](https://tmsf.med.harvard.edu/core/www/modules/sdig/index.php?value=11/60&pvkey=&run_id=92259&search_id=93773&scans_id=92255&peptide_id=1562&charge=3&scanf=10708&mz_l=200.155&mz_h=400.155) \| [1549.881](https://tmsf.med.harvard.edu/core/www/modules/sdig/index.php?value=11/60&pvkey=&run_id=92259&search_id=93773&scans_id=92255&peptide_id=1562&charge=3&scanf=10708&mz_l=1449.881&mz_h=1649.881) \| **---** \| **14** \| \| **I** \| **4** \| **---** \| [413.239](https://tmsf.med.harvard.edu/core/www/modules/sdig/index.php?value=11/60&pvkey=&run_id=92259&search_id=93773&scans_id=92255&peptide_id=1562&charge=3&scanf=10708&mz_l=313.239&mz_h=513.239) \| [1436.797](https://tmsf.med.harvard.edu/core/www/modules/sdig/index.php?value=11/60&pvkey=&run_id=92259&search_id=93773&scans_id=92255&peptide_id=1562&charge=3&scanf=10708&mz_l=1336.797&mz_h=1536.797) \| **---** \| **13** \| \| **Q** \| **5** \| **-3.164** \| [**541.298**](https://tmsf.med.harvard.edu/core/www/modules/sdig/index.php?value=11/60&pvkey=&run_id=92259&search_id=93773&scans_id=92255&peptide_id=1562&charge=3&scanf=10708&mz_l=441.298&mz_h=641.298) \| [1323.713](https://tmsf.med.harvard.edu/core/www/modules/sdig/index.php?value=11/60&pvkey=&run_id=92259&search_id=93773&scans_id=92255&peptide_id=1562&charge=3&scanf=10708&mz_l=1223.713&mz_h=1423.713) \| **---** \| **12** \| \| **H** \| **6** \| **5.734** \| [**678.357**](https://tmsf.med.harvard.edu/core/www/modules/sdig/index.php?value=11/60&pvkey=&run_id=92259&search_id=93773&scans_id=92255&peptide_id=1562&charge=3&scanf=10708&mz_l=578.357&mz_h=778.357) \| [**1195.654**](https://tmsf.med.harvard.edu/core/www/modules/sdig/index.php?value=11/60&pvkey=&run_id=92259&search_id=93773&scans_id=92255&peptide_id=1562&charge=3&scanf=10708&mz_l=1095.654&mz_h=1295.654) \| **-0.426** \| **11** \| \| **S** \| **7** \| **-7.259** \| [**765.389**](https://tmsf.med.harvard.edu/core/www/modules/sdig/index.php?value=11/60&pvkey=&run_id=92259&search_id=93773&scans_id=92255&peptide_id=1562&charge=3&scanf=10708&mz_l=665.389&mz_h=865.389) \| [**1058.595**](https://tmsf.med.harvard.edu/core/www/modules/sdig/index.php?value=11/60&pvkey=&run_id=92259&search_id=93773&scans_id=92255&peptide_id=1562&charge=3&scanf=10708&mz_l=958.595&mz_h=1158.595) \| **2.011** \| **10** \| \| **T** \| **8** \| **1.607** \| [**866.437**](https://tmsf.med.harvard.edu/core/www/modules/sdig/index.php?value=11/60&pvkey=&run_id=92259&search_id=93773&scans_id=92255&peptide_id=1562&charge=3&scanf=10708&mz_l=766.437&mz_h=966.437) \| [**971.563**](https://tmsf.med.harvard.edu/core/www/modules/sdig/index.php?value=11/60&pvkey=&run_id=92259&search_id=93773&scans_id=92255&peptide_id=1562&charge=3&scanf=10708&mz_l=871.563&mz_h=1071.563) \| **-1.971** \| **9** \| \| **I** \| **9** \| **3.770** \| [**979.521**](https://tmsf.med.harvard.edu/core/www/modules/sdig/index.php?value=11/60&pvkey=&run_id=92259&search_id=93773&scans_id=92255&peptide_id=1562&charge=3&scanf=10708&mz_l=879.521&mz_h=1079.521) \| [**870.516**](https://tmsf.med.harvard.edu/core/www/modules/sdig/index.php?value=11/60&pvkey=&run_id=92259&search_id=93773&scans_id=92255&peptide_id=1562&charge=3&scanf=10708&mz_l=770.516&mz_h=970.516) \| **6.365** \| **8** \| \| **N** \| **10** \| **-1.943** \| [**1093.564**](https://tmsf.med.harvard.edu/core/www/modules/sdig/index.php?value=11/60&pvkey=&run_id=92259&search_id=93773&scans_id=92255&peptide_id=1562&charge=3&scanf=10708&mz_l=993.564&mz_h=1193.564) \| [**757.432**](https://tmsf.med.harvard.edu/core/www/modules/sdig/index.php?value=11/60&pvkey=&run_id=92259&search_id=93773&scans_id=92255&peptide_id=1562&charge=3&scanf=10708&mz_l=657.432&mz_h=857.432) \| **0.330** \| **7** \| \| **N** \| **11** \| **0.296** \| [**1207.607**](https://tmsf.med.harvard.edu/core/www/modules/sdig/index.php?value=11/60&pvkey=&run_id=92259&search_id=93773&scans_id=92255&peptide_id=1562&charge=3&scanf=10708&mz_l=1107.607&mz_h=1307.607) \| [**643.389**](https://tmsf.med.harvard.edu/core/www/modules/sdig/index.php?value=11/60&pvkey=&run_id=92259&search_id=93773&scans_id=92255&peptide_id=1562&charge=3&scanf=10708&mz_l=543.389&mz_h=743.389) \| **-0.245** \| **6** \| \| **L** \| **12** \| **---** \| [1320.691](https://tmsf.med.harvard.edu/core/www/modules/sdig/index.php?value=11/60&pvkey=&run_id=92259&search_id=93773&scans_id=92255&peptide_id=1562&charge=3&scanf=10708&mz_l=1220.691&mz_h=1420.691) \| [**529.346**](https://tmsf.med.harvard.edu/core/www/modules/sdig/index.php?value=11/60&pvkey=&run_id=92259&search_id=93773&scans_id=92255&peptide_id=1562&charge=3&scanf=10708&mz_l=429.346&mz_h=629.346) \| **-1.644** \| **5** \| \| **A** \| **13** \| **---** \| [1391.728](https://tmsf.med.harvard.edu/core/www/modules/sdig/index.php?value=11/60&pvkey=&run_id=92259&search_id=93773&scans_id=92255&peptide_id=1562&charge=3&scanf=10708&mz_l=1291.728&mz_h=1491.728) \| [**416.262**](https://tmsf.med.harvard.edu/core/www/modules/sdig/index.php?value=11/60&pvkey=&run_id=92259&search_id=93773&scans_id=92255&peptide_id=1562&charge=3&scanf=10708&mz_l=316.262&mz_h=516.262) \| **-1.020** \| **4** \| \| **I** \| **14** \| **---** \| [1504.812](https://tmsf.med.harvard.edu/core/www/modules/sdig/index.php?value=11/60&pvkey=&run_id=92259&search_id=93773&scans_id=92255&peptide_id=1562&charge=3&scanf=10708&mz_l=1404.812&mz_h=1604.812) \| [**345.224**](https://tmsf.med.harvard.edu/core/www/modules/sdig/index.php?value=11/60&pvkey=&run_id=92259&search_id=93773&scans_id=92255&peptide_id=1562&charge=3&scanf=10708&mz_l=245.224&mz_h=445.224) \| **-0.687** \| **3** \| \| **G** \| **15** \| **---** \| [1561.833](https://tmsf.med.harvard.edu/core/www/modules/sdig/index.php?value=11/60&pvkey=&run_id=92259&search_id=93773&scans_id=92255&peptide_id=1562&charge=3&scanf=10708&mz_l=1461.833&mz_h=1661.833) \| [**232.140**](https://tmsf.med.harvard.edu/core/www/modules/sdig/index.php?value=11/60&pvkey=&run_id=92259&search_id=93773&scans_id=92255&peptide_id=1562&charge=3&scanf=10708&mz_l=132.14&mz_h=332.14) \| **-0.284** \| **2** \| \| **R** \| **16** \| **---** \| --- \| [**175.119**](https://tmsf.med.harvard.edu/core/www/modules/sdig/index.php?value=11/60&pvkey=&run_id=92259&search_id=93773&scans_id=92255&peptide_id=1562&charge=3&scanf=10708&mz_l=75.119&mz_h=275.119) \| **-0.494** \| **1** \|   **+2**   \| **Seq** \| **#** \| **b: Δ Error** \| **b** \| **y** \| **y: Δ Error** \| **+1** \| \| --- \| --- \| --- \| --- \| --- \| --- \| --- \| \| **E** \| **1** \| **---** \| [65.529](https://tmsf.med.harvard.edu/core/www/modules/sdig/index.php?value=11/60&pvkey=&run_id=92259&search_id=93773&scans_id=92255&peptide_id=1562&charge=3&scanf=10708&mz_l=-34.471&mz_h=165.529) \| --- \| **---** \| **16** \| \| **G** \| **2** \| **---** \| [94.039](https://tmsf.med.harvard.edu/core/www/modules/sdig/index.php?value=11/60&pvkey=&run_id=92259&search_id=93773&scans_id=92255&peptide_id=1562&charge=3&scanf=10708&mz_l=-5.961&mz_h=194.039) \| [**803.955**](https://tmsf.med.harvard.edu/core/www/modules/sdig/index.php?value=11/60&pvkey=&run_id=92259&search_id=93773&scans_id=92255&peptide_id=1562&charge=3&scanf=10708&mz_l=703.955&mz_h=903.955) \| **2.902** \| **15** \| \| **I** \| **3** \| **---** \| [150.581](https://tmsf.med.harvard.edu/core/www/modules/sdig/index.php?value=11/60&pvkey=&run_id=92259&search_id=93773&scans_id=92255&peptide_id=1562&charge=3&scanf=10708&mz_l=50.581&mz_h=250.581) \| [775.444](https://tmsf.med.harvard.edu/core/www/modules/sdig/index.php?value=11/60&pvkey=&run_id=92259&search_id=93773&scans_id=92255&peptide_id=1562&charge=3&scanf=10708&mz_l=675.444&mz_h=875.444) \| **---** \| **14** \| \| **I** \| **4** \| **-52.903** \| [**207.123**](https://tmsf.med.harvard.edu/core/www/modules/sdig/index.php?value=11/60&pvkey=&run_id=92259&search_id=93773&scans_id=92255&peptide_id=1562&charge=3&scanf=10708&mz_l=107.123&mz_h=307.123) \| [**718.902**](https://tmsf.med.harvard.edu/core/www/modules/sdig/index.php?value=11/60&pvkey=&run_id=92259&search_id=93773&scans_id=92255&peptide_id=1562&charge=3&scanf=10708&mz_l=618.902&mz_h=818.902) \| **-2.232** \| **13** \| \| **Q** \| **5** \| **---** \| [271.153](https://tmsf.med.harvard.edu/core/www/modules/sdig/index.php?value=11/60&pvkey=&run_id=92259&search_id=93773&scans_id=92255&peptide_id=1562&charge=3&scanf=10708&mz_l=171.153&mz_h=371.153) \| [**662.360**](https://tmsf.med.harvard.edu/core/www/modules/sdig/index.php?value=11/60&pvkey=&run_id=92259&search_id=93773&scans_id=92255&peptide_id=1562&charge=3&scanf=10708&mz_l=562.36&mz_h=762.36) \| **-0.059** \| **12** \| \| **H** \| **6** \| **---** \| [339.682](https://tmsf.med.harvard.edu/core/www/modules/sdig/index.php?value=11/60&pvkey=&run_id=92259&search_id=93773&scans_id=92255&peptide_id=1562&charge=3&scanf=10708&mz_l=239.682&mz_h=439.682) \| [**598.331**](https://tmsf.med.harvard.edu/core/www/modules/sdig/index.php?value=11/60&pvkey=&run_id=92259&search_id=93773&scans_id=92255&peptide_id=1562&charge=3&scanf=10708&mz_l=498.331&mz_h=698.331) \| **7.367** \| **11** \| \| **S** \| **7** \| **-13.876** \| [**383.198**](https://tmsf.med.harvard.edu/core/www/modules/sdig/index.php?value=11/60&pvkey=&run_id=92259&search_id=93773&scans_id=92255&peptide_id=1562&charge=3&scanf=10708&mz_l=283.198&mz_h=483.198) \| [529.801](https://tmsf.med.harvard.edu/core/www/modules/sdig/index.php?value=11/60&pvkey=&run_id=92259&search_id=93773&scans_id=92255&peptide_id=1562&charge=3&scanf=10708&mz_l=429.801&mz_h=629.801) \| **---** \| **10** \| \| **T** \| **8** \| **---** \| [433.722](https://tmsf.med.harvard.edu/core/www/modules/sdig/index.php?value=11/60&pvkey=&run_id=92259&search_id=93773&scans_id=92255&peptide_id=1562&charge=3&scanf=10708&mz_l=333.722&mz_h=533.722) \| [486.285](https://tmsf.med.harvard.edu/core/www/modules/sdig/index.php?value=11/60&pvkey=&run_id=92259&search_id=93773&scans_id=92255&peptide_id=1562&charge=3&scanf=10708&mz_l=386.285&mz_h=586.285) \| **---** \| **9** \| \| **I** \| **9** \| **-3.343** \| [**490.264**](https://tmsf.med.harvard.edu/core/www/modules/sdig/index.php?value=11/60&pvkey=&run_id=92259&search_id=93773&scans_id=92255&peptide_id=1562&charge=3&scanf=10708&mz_l=390.264&mz_h=590.264) \| [435.761](https://tmsf.med.harvard.edu/core/www/modules/sdig/index.php?value=11/60&pvkey=&run_id=92259&search_id=93773&scans_id=92255&peptide_id=1562&charge=3&scanf=10708&mz_l=335.761&mz_h=535.761) \| **---** \| **8** \| \| **N** \| **10** \| **-4.797** \| [**547.285**](https://tmsf.med.harvard.edu/core/www/modules/sdig/index.php?value=11/60&pvkey=&run_id=92259&search_id=93773&scans_id=92255&peptide_id=1562&charge=3&scanf=10708&mz_l=447.285&mz_h=647.285) \| [**379.219**](https://tmsf.med.harvard.edu/core/www/modules/sdig/index.php?value=11/60&pvkey=&run_id=92259&search_id=93773&scans_id=92255&peptide_id=1562&charge=3&scanf=10708&mz_l=279.219&mz_h=479.219) \| **-28.901** \| **7** \| \| **N** \| **11** \| **20.535** \| [**604.307**](https://tmsf.med.harvard.edu/core/www/modules/sdig/index.php?value=11/60&pvkey=&run_id=92259&search_id=93773&scans_id=92255&peptide_id=1562&charge=3&scanf=10708&mz_l=504.307&mz_h=704.307) \| [322.198](https://tmsf.med.harvard.edu/core/www/modules/sdig/index.php?value=11/60&pvkey=&run_id=92259&search_id=93773&scans_id=92255&peptide_id=1562&charge=3&scanf=10708&mz_l=222.198&mz_h=422.198) \| **---** \| **6** \| \| **L** \| **12** \| **3.479** \| [**660.849**](https://tmsf.med.harvard.edu/core/www/modules/sdig/index.php?value=11/60&pvkey=&run_id=92259&search_id=93773&scans_id=92255&peptide_id=1562&charge=3&scanf=10708&mz_l=560.849&mz_h=760.849) \| [**265.176**](https://tmsf.med.harvard.edu/core/www/modules/sdig/index.php?value=11/60&pvkey=&run_id=92259&search_id=93773&scans_id=92255&peptide_id=1562&charge=3&scanf=10708&mz_l=165.176&mz_h=365.176) \| **-1.896** \| **5** \| \| **A** \| **13** \| **---** \| [696.368](https://tmsf.med.harvard.edu/core/www/modules/sdig/index.php?value=11/60&pvkey=&run_id=92259&search_id=93773&scans_id=92255&peptide_id=1562&charge=3&scanf=10708&mz_l=596.368&mz_h=796.368) \| [208.634](https://tmsf.med.harvard.edu/core/www/modules/sdig/index.php?value=11/60&pvkey=&run_id=92259&search_id=93773&scans_id=92255&peptide_id=1562&charge=3&scanf=10708&mz_l=108.634&mz_h=308.634) \| **---** \| **4** \| \| **I** \| **14** \| **---** \| [752.910](https://tmsf.med.harvard.edu/core/www/modules/sdig/index.php?value=11/60&pvkey=&run_id=92259&search_id=93773&scans_id=92255&peptide_id=1562&charge=3&scanf=10708&mz_l=652.91&mz_h=852.91) \| [**173.116**](https://tmsf.med.harvard.edu/core/www/modules/sdig/index.php?value=11/60&pvkey=&run_id=92259&search_id=93773&scans_id=92255&peptide_id=1562&charge=3&scanf=10708&mz_l=73.116&mz_h=273.116) \| **71.902** \| **3** \| \| **G** \| **15** \| **---** \| [781.420](https://tmsf.med.harvard.edu/core/www/modules/sdig/index.php?value=11/60&pvkey=&run_id=92259&search_id=93773&scans_id=92255&peptide_id=1562&charge=3&scanf=10708&mz_l=681.42&mz_h=881.42) \| [116.574](https://tmsf.med.harvard.edu/core/www/modules/sdig/index.php?value=11/60&pvkey=&run_id=92259&search_id=93773&scans_id=92255&peptide_id=1562&charge=3&scanf=10708&mz_l=16.574&mz_h=216.574) \| **---** \| **2** \| \| **R** \| **16** \| **---** \| --- \| [88.063](https://tmsf.med.harvard.edu/core/www/modules/sdig/index.php?value=11/60&pvkey=&run_id=92259&search_id=93773&scans_id=92255&peptide_id=1562&charge=3&scanf=10708&mz_l=-11.937&mz_h=188.063) \| **---** \| **1** \| |

**Sequence no 4**

| [>tr\|A0A1U8IAU3\|A0A1U8IAU3_GOSHI Basic 7S globulin-like OS=Gossypium hirsutum OX=3635 GN=LOC107894474 PE=3 SV=1](http://www.uniprot.org/uniprot/?query=A0A1U8IAU3_GOSHI) |
| --- |
| MASSFLLFLL IFLSVSSFIL LSESQKTSKP NRFILQLQKD PKTKLYVTNI YKRTPSQKVP FVVDLNGRLL WVTCEKSYRS STYHAPRCHS TQCSRAGSHY  CHICSTRDGP GCHNNTCGVM SMNPVTGLTA MSELAQDVLS IQSTQGSNPG PMVRVPQLLF TCAPSLLLQR GLPSTVQGVA GLGHSLISLP TQLTSHFSSA  GFAPIFALCL APKGVMFFGD SPYYMLPNVD ITRPLSYTPL IISPQGEYYM EVKSIKINDK DVPIDTALLS INKQGVGGTK LSTINPYTIL HHSIFKAVTQ  FFSKELSAIP QVKPVAPFGV CFKSKSIKNS RVGLEVPNID LVLHDKHVMW RIYGANSIVE AAPGVSCLAF VDGGMDNNGA SIIIGAYQME NNLVQFDKAR  SRLGFSSSLL FYKTSCNNFN FTAIP |

| Legend | | |
| --- | --- | --- |
| THISREFERE NCE | UNMATCHED | OTHERREFER ENCES |

| \| 46496.83 Da \| Protein Coverage \| \| \| --- \| --- \| --- \| \| 425 AA \| 77 AA \| **18.12 %** \| |
| --- | --- | --- | --- | --- | --- | --- |

**Data table 9**

| \| **Sequence** \| \| --- \| \| LGFSSSLLFYK \| |
| --- | --- | --- |
| **Predicted Fragmentation Pattern**   \| **Seq** \| **#** \| **b: Δ Error** \| **b** \| **y** \| **y: Δ Error** \| **+1** \| \| --- \| --- \| --- \| --- \| --- \| --- \| --- \| \| **L** \| **1** \| **---** \| [114.091](https://tmsf.med.harvard.edu/core/www/modules/sdig/index.php?value=14/20&pvkey=&run_id=92259&search_id=93773&scans_id=92255&peptide_id=6588&charge=2&scanf=15637&mz_l=14.091&mz_h=214.091) \| --- \| **---** \| **11** \| \| **G** \| **2** \| **0.205** \| [**171.113**](https://tmsf.med.harvard.edu/core/www/modules/sdig/index.php?value=14/20&pvkey=&run_id=92259&search_id=93773&scans_id=92255&peptide_id=6588&charge=2&scanf=15637&mz_l=71.113&mz_h=271.113) \| [**1148.599**](https://tmsf.med.harvard.edu/core/www/modules/sdig/index.php?value=14/20&pvkey=&run_id=92259&search_id=93773&scans_id=92255&peptide_id=6588&charge=2&scanf=15637&mz_l=1048.599&mz_h=1248.599) \| **-0.536** \| **10** \| \| **F** \| **3** \| **-0.972** \| [**318.181**](https://tmsf.med.harvard.edu/core/www/modules/sdig/index.php?value=14/20&pvkey=&run_id=92259&search_id=93773&scans_id=92255&peptide_id=6588&charge=2&scanf=15637&mz_l=218.181&mz_h=418.181) \| [**1091.577**](https://tmsf.med.harvard.edu/core/www/modules/sdig/index.php?value=14/20&pvkey=&run_id=92259&search_id=93773&scans_id=92255&peptide_id=6588&charge=2&scanf=15637&mz_l=991.577&mz_h=1191.577) \| **-0.694** \| **9** \| \| **S** \| **4** \| **-0.802** \| [**405.213**](https://tmsf.med.harvard.edu/core/www/modules/sdig/index.php?value=14/20&pvkey=&run_id=92259&search_id=93773&scans_id=92255&peptide_id=6588&charge=2&scanf=15637&mz_l=305.213&mz_h=505.213) \| [**944.509**](https://tmsf.med.harvard.edu/core/www/modules/sdig/index.php?value=14/20&pvkey=&run_id=92259&search_id=93773&scans_id=92255&peptide_id=6588&charge=2&scanf=15637&mz_l=844.509&mz_h=1044.509) \| **-0.616** \| **8** \| \| **S** \| **5** \| **---** \| [492.245](https://tmsf.med.harvard.edu/core/www/modules/sdig/index.php?value=14/20&pvkey=&run_id=92259&search_id=93773&scans_id=92255&peptide_id=6588&charge=2&scanf=15637&mz_l=392.245&mz_h=592.245) \| [**857.477**](https://tmsf.med.harvard.edu/core/www/modules/sdig/index.php?value=14/20&pvkey=&run_id=92259&search_id=93773&scans_id=92255&peptide_id=6588&charge=2&scanf=15637&mz_l=757.477&mz_h=957.477) \| **-0.624** \| **7** \| \| **S** \| **6** \| **-0.035** \| [**579.277**](https://tmsf.med.harvard.edu/core/www/modules/sdig/index.php?value=14/20&pvkey=&run_id=92259&search_id=93773&scans_id=92255&peptide_id=6588&charge=2&scanf=15637&mz_l=479.277&mz_h=679.277) \| [**770.445**](https://tmsf.med.harvard.edu/core/www/modules/sdig/index.php?value=14/20&pvkey=&run_id=92259&search_id=93773&scans_id=92255&peptide_id=6588&charge=2&scanf=15637&mz_l=670.445&mz_h=870.445) \| **0.632** \| **6** \| \| **L** \| **7** \| **-6.932** \| [**692.361**](https://tmsf.med.harvard.edu/core/www/modules/sdig/index.php?value=14/20&pvkey=&run_id=92259&search_id=93773&scans_id=92255&peptide_id=6588&charge=2&scanf=15637&mz_l=592.361&mz_h=792.361) \| [**683.413**](https://tmsf.med.harvard.edu/core/www/modules/sdig/index.php?value=14/20&pvkey=&run_id=92259&search_id=93773&scans_id=92255&peptide_id=6588&charge=2&scanf=15637&mz_l=583.413&mz_h=783.413) \| **3.102** \| **5** \| \| **L** \| **8** \| **---** \| [805.445](https://tmsf.med.harvard.edu/core/www/modules/sdig/index.php?value=14/20&pvkey=&run_id=92259&search_id=93773&scans_id=92255&peptide_id=6588&charge=2&scanf=15637&mz_l=705.445&mz_h=905.445) \| [**570.329**](https://tmsf.med.harvard.edu/core/www/modules/sdig/index.php?value=14/20&pvkey=&run_id=92259&search_id=93773&scans_id=92255&peptide_id=6588&charge=2&scanf=15637&mz_l=470.329&mz_h=670.329) \| **0.967** \| **4** \| \| **F** \| **9** \| **---** \| [952.514](https://tmsf.med.harvard.edu/core/www/modules/sdig/index.php?value=14/20&pvkey=&run_id=92259&search_id=93773&scans_id=92255&peptide_id=6588&charge=2&scanf=15637&mz_l=852.514&mz_h=1052.514) \| [**457.245**](https://tmsf.med.harvard.edu/core/www/modules/sdig/index.php?value=14/20&pvkey=&run_id=92259&search_id=93773&scans_id=92255&peptide_id=6588&charge=2&scanf=15637&mz_l=357.245&mz_h=557.245) \| **-0.288** \| **3** \| \| **Y** \| **10** \| **---** \| [1115.577](https://tmsf.med.harvard.edu/core/www/modules/sdig/index.php?value=14/20&pvkey=&run_id=92259&search_id=93773&scans_id=92255&peptide_id=6588&charge=2&scanf=15637&mz_l=1015.577&mz_h=1215.577) \| [**310.176**](https://tmsf.med.harvard.edu/core/www/modules/sdig/index.php?value=14/20&pvkey=&run_id=92259&search_id=93773&scans_id=92255&peptide_id=6588&charge=2&scanf=15637&mz_l=210.176&mz_h=410.176) \| **-0.642** \| **2** \| \| **K** \| **11** \| **---** \| --- \| [**147.113**](https://tmsf.med.harvard.edu/core/www/modules/sdig/index.php?value=14/20&pvkey=&run_id=92259&search_id=93773&scans_id=92255&peptide_id=6588&charge=2&scanf=15637&mz_l=47.113&mz_h=247.113) \| **0.129** \| **1** \| \|  \|  \|  \|  \|  \|  \|  \| |

**Sequence no 5**

| [>tr\|A0A4Y1RTK3\|A0A4Y1RTK3_PRUDU phosphopyruvate hydratase OS=Prunus dulcis OX=3755 GN=ALMOND_2B032175 PE=3 SV=1](http://www.uniprot.org/uniprot/?query=A0A4Y1RTK3_PRUDU) |
| --- |
| MATIQTVKAR QIFDSRGNPT VEVDIVLSDG TLARAAVPSG ASTGVYEALE LRDGGSDYLG KGVSKAVNNV NSIIGPALIG KDPSEQTAID NFMVQQLDGT  VNEWGWCKQK LGANAILAVS LAVAKAGASV KKIPLYKHIA NLAGNKNLVL PVPAFNVING GSHAGNKLAM QEFMILPVGA SSFKEAMKMG VEVYHHLKAV  IKKKYGQDAT NVGDEGGFAP NIQENKEGLE LLKTAIEKAG YTGKVVIGMD VAASEFYGSD QTYDLNFKEE KNDGSQKISG NALKDLYKSF VSEYPIVSIE  DPFDQDDWEH YAKMTAECGE QVQIVGDDLL VTNPKRVEKA IKEKSCNALL LKVNQIGSVT ESIEAVRMSK KAGWGVMASH RSGETEDTFI ADLSVGLATG  QIKTGAPCRS ERLAKYNQLL RIEEELGAEA VYAGAKFRVP VEPY |

| Legend | | |
| --- | --- | --- |
| THISREFERE NCE | UNMATCHED | OTHERREFER ENCES |

| \| 47737.46 Da \| Protein Coverage \| \| \| --- \| --- \| --- \| \| 444 AA \| 22 AA \| **4.95 %** \| |
| --- | --- | --- | --- | --- | --- | --- |

**Data table 10**

**Sequence**

| \| SGETEDTFIADLSVGLATGQIK \| \| --- \| |
| --- | --- |
| **Predicted Fragmentation Pattern**  **+1**   \| **Seq** \| **#** \| **b: Δ Error** \| **b** \| **y** \| **y: Δ Error** \| **+1** \| \| --- \| --- \| --- \| --- \| --- \| --- \| --- \| \| **S** \| **1** \| **---** \| [88.039](https://tmsf.med.harvard.edu/core/www/modules/sdig/index.php?value=14/84&pvkey=&run_id=92258&search_id=93772&scans_id=92254&peptide_id=7739&charge=3&scanf=16751&mz_l=-11.961&mz_h=188.039&show_mass_error=1&match_percentile=35&precision=3&soleil_threshold=0&soleil_sort=m/z&opt_show_bion_1=1&opt_label_bion_1=1&opt_show_yion_1=1&opt_label_yion_1=1&opt_show_bion_2=1&opt_label_bion_2=1&opt_show_yion_2=1&opt_label_yion_2=1&opt_show_pre=1&opt_only_matches=1&opt_label_pre=0&opt_show_soleil=0&opt_pin_soleil=1&old_zoomlevel=0&zoomlevel=3&old_izoomlevel=0&izoomlevel=0&label_method=0&peak_font_size=&action=panright) \| --- \| **---** \| **22** \| \| **G** \| **2** \| **-1.736** \| [**145.061**](https://tmsf.med.harvard.edu/core/www/modules/sdig/index.php?value=14/84&pvkey=&run_id=92258&search_id=93772&scans_id=92254&peptide_id=7739&charge=3&scanf=16751&mz_l=45.061&mz_h=245.061&show_mass_error=1&match_percentile=35&precision=3&soleil_threshold=0&soleil_sort=m/z&opt_show_bion_1=1&opt_label_bion_1=1&opt_show_yion_1=1&opt_label_yion_1=1&opt_show_bion_2=1&opt_label_bion_2=1&opt_show_yion_2=1&opt_label_yion_2=1&opt_show_pre=1&opt_only_matches=1&opt_label_pre=0&opt_show_soleil=0&opt_pin_soleil=1&old_zoomlevel=0&zoomlevel=3&old_izoomlevel=0&izoomlevel=0&label_method=0&peak_font_size=&action=panright) \| [2165.097](https://tmsf.med.harvard.edu/core/www/modules/sdig/index.php?value=14/84&pvkey=&run_id=92258&search_id=93772&scans_id=92254&peptide_id=7739&charge=3&scanf=16751&mz_l=2065.097&mz_h=2265.097&show_mass_error=1&match_percentile=35&precision=3&soleil_threshold=0&soleil_sort=m/z&opt_show_bion_1=1&opt_label_bion_1=1&opt_show_yion_1=1&opt_label_yion_1=1&opt_show_bion_2=1&opt_label_bion_2=1&opt_show_yion_2=1&opt_label_yion_2=1&opt_show_pre=1&opt_only_matches=1&opt_label_pre=0&opt_show_soleil=0&opt_pin_soleil=1&old_zoomlevel=0&zoomlevel=3&old_izoomlevel=0&izoomlevel=0&label_method=0&peak_font_size=&action=panright) \| **---** \| **21** \| \| **E** \| **3** \| **0.341** \| [**274.103**](https://tmsf.med.harvard.edu/core/www/modules/sdig/index.php?value=14/84&pvkey=&run_id=92258&search_id=93772&scans_id=92254&peptide_id=7739&charge=3&scanf=16751&mz_l=174.103&mz_h=374.103&show_mass_error=1&match_percentile=35&precision=3&soleil_threshold=0&soleil_sort=m/z&opt_show_bion_1=1&opt_label_bion_1=1&opt_show_yion_1=1&opt_label_yion_1=1&opt_show_bion_2=1&opt_label_bion_2=1&opt_show_yion_2=1&opt_label_yion_2=1&opt_show_pre=1&opt_only_matches=1&opt_label_pre=0&opt_show_soleil=0&opt_pin_soleil=1&old_zoomlevel=0&zoomlevel=3&old_izoomlevel=0&izoomlevel=0&label_method=0&peak_font_size=&action=panright) \| [2108.076](https://tmsf.med.harvard.edu/core/www/modules/sdig/index.php?value=14/84&pvkey=&run_id=92258&search_id=93772&scans_id=92254&peptide_id=7739&charge=3&scanf=16751&mz_l=2008.076&mz_h=2208.076&show_mass_error=1&match_percentile=35&precision=3&soleil_threshold=0&soleil_sort=m/z&opt_show_bion_1=1&opt_label_bion_1=1&opt_show_yion_1=1&opt_label_yion_1=1&opt_show_bion_2=1&opt_label_bion_2=1&opt_show_yion_2=1&opt_label_yion_2=1&opt_show_pre=1&opt_only_matches=1&opt_label_pre=0&opt_show_soleil=0&opt_pin_soleil=1&old_zoomlevel=0&zoomlevel=3&old_izoomlevel=0&izoomlevel=0&label_method=0&peak_font_size=&action=panright) \| **---** \| **20** \| \| **T** \| **4** \| **---** \| [375.151](https://tmsf.med.harvard.edu/core/www/modules/sdig/index.php?value=14/84&pvkey=&run_id=92258&search_id=93772&scans_id=92254&peptide_id=7739&charge=3&scanf=16751&mz_l=275.151&mz_h=475.151&show_mass_error=1&match_percentile=35&precision=3&soleil_threshold=0&soleil_sort=m/z&opt_show_bion_1=1&opt_label_bion_1=1&opt_show_yion_1=1&opt_label_yion_1=1&opt_show_bion_2=1&opt_label_bion_2=1&opt_show_yion_2=1&opt_label_yion_2=1&opt_show_pre=1&opt_only_matches=1&opt_label_pre=0&opt_show_soleil=0&opt_pin_soleil=1&old_zoomlevel=0&zoomlevel=3&old_izoomlevel=0&izoomlevel=0&label_method=0&peak_font_size=&action=panright) \| [1979.033](https://tmsf.med.harvard.edu/core/www/modules/sdig/index.php?value=14/84&pvkey=&run_id=92258&search_id=93772&scans_id=92254&peptide_id=7739&charge=3&scanf=16751&mz_l=1879.033&mz_h=2079.033&show_mass_error=1&match_percentile=35&precision=3&soleil_threshold=0&soleil_sort=m/z&opt_show_bion_1=1&opt_label_bion_1=1&opt_show_yion_1=1&opt_label_yion_1=1&opt_show_bion_2=1&opt_label_bion_2=1&opt_show_yion_2=1&opt_label_yion_2=1&opt_show_pre=1&opt_only_matches=1&opt_label_pre=0&opt_show_soleil=0&opt_pin_soleil=1&old_zoomlevel=0&zoomlevel=3&old_izoomlevel=0&izoomlevel=0&label_method=0&peak_font_size=&action=panright) \| **---** \| **19** \| \| **E** \| **5** \| **4.421** \| [**504.194**](https://tmsf.med.harvard.edu/core/www/modules/sdig/index.php?value=14/84&pvkey=&run_id=92258&search_id=93772&scans_id=92254&peptide_id=7739&charge=3&scanf=16751&mz_l=404.194&mz_h=604.194&show_mass_error=1&match_percentile=35&precision=3&soleil_threshold=0&soleil_sort=m/z&opt_show_bion_1=1&opt_label_bion_1=1&opt_show_yion_1=1&opt_label_yion_1=1&opt_show_bion_2=1&opt_label_bion_2=1&opt_show_yion_2=1&opt_label_yion_2=1&opt_show_pre=1&opt_only_matches=1&opt_label_pre=0&opt_show_soleil=0&opt_pin_soleil=1&old_zoomlevel=0&zoomlevel=3&old_izoomlevel=0&izoomlevel=0&label_method=0&peak_font_size=&action=panright) \| [1877.985](https://tmsf.med.harvard.edu/core/www/modules/sdig/index.php?value=14/84&pvkey=&run_id=92258&search_id=93772&scans_id=92254&peptide_id=7739&charge=3&scanf=16751&mz_l=1777.985&mz_h=1977.985&show_mass_error=1&match_percentile=35&precision=3&soleil_threshold=0&soleil_sort=m/z&opt_show_bion_1=1&opt_label_bion_1=1&opt_show_yion_1=1&opt_label_yion_1=1&opt_show_bion_2=1&opt_label_bion_2=1&opt_show_yion_2=1&opt_label_yion_2=1&opt_show_pre=1&opt_only_matches=1&opt_label_pre=0&opt_show_soleil=0&opt_pin_soleil=1&old_zoomlevel=0&zoomlevel=3&old_izoomlevel=0&izoomlevel=0&label_method=0&peak_font_size=&action=panright) \| **---** \| **18** \| \| **D** \| **6** \| **3.557** \| [**619.221**](https://tmsf.med.harvard.edu/core/www/modules/sdig/index.php?value=14/84&pvkey=&run_id=92258&search_id=93772&scans_id=92254&peptide_id=7739&charge=3&scanf=16751&mz_l=519.221&mz_h=719.221&show_mass_error=1&match_percentile=35&precision=3&soleil_threshold=0&soleil_sort=m/z&opt_show_bion_1=1&opt_label_bion_1=1&opt_show_yion_1=1&opt_label_yion_1=1&opt_show_bion_2=1&opt_label_bion_2=1&opt_show_yion_2=1&opt_label_yion_2=1&opt_show_pre=1&opt_only_matches=1&opt_label_pre=0&opt_show_soleil=0&opt_pin_soleil=1&old_zoomlevel=0&zoomlevel=3&old_izoomlevel=0&izoomlevel=0&label_method=0&peak_font_size=&action=panright) \| [1748.943](https://tmsf.med.harvard.edu/core/www/modules/sdig/index.php?value=14/84&pvkey=&run_id=92258&search_id=93772&scans_id=92254&peptide_id=7739&charge=3&scanf=16751&mz_l=1648.943&mz_h=1848.943&show_mass_error=1&match_percentile=35&precision=3&soleil_threshold=0&soleil_sort=m/z&opt_show_bion_1=1&opt_label_bion_1=1&opt_show_yion_1=1&opt_label_yion_1=1&opt_show_bion_2=1&opt_label_bion_2=1&opt_show_yion_2=1&opt_label_yion_2=1&opt_show_pre=1&opt_only_matches=1&opt_label_pre=0&opt_show_soleil=0&opt_pin_soleil=1&old_zoomlevel=0&zoomlevel=3&old_izoomlevel=0&izoomlevel=0&label_method=0&peak_font_size=&action=panright) \| **---** \| **17** \| \| **T** \| **7** \| **-2.718** \| [**720.268**](https://tmsf.med.harvard.edu/core/www/modules/sdig/index.php?value=14/84&pvkey=&run_id=92258&search_id=93772&scans_id=92254&peptide_id=7739&charge=3&scanf=16751&mz_l=620.268&mz_h=820.268&show_mass_error=1&match_percentile=35&precision=3&soleil_threshold=0&soleil_sort=m/z&opt_show_bion_1=1&opt_label_bion_1=1&opt_show_yion_1=1&opt_label_yion_1=1&opt_show_bion_2=1&opt_label_bion_2=1&opt_show_yion_2=1&opt_label_yion_2=1&opt_show_pre=1&opt_only_matches=1&opt_label_pre=0&opt_show_soleil=0&opt_pin_soleil=1&old_zoomlevel=0&zoomlevel=3&old_izoomlevel=0&izoomlevel=0&label_method=0&peak_font_size=&action=panright) \| [1633.916](https://tmsf.med.harvard.edu/core/www/modules/sdig/index.php?value=14/84&pvkey=&run_id=92258&search_id=93772&scans_id=92254&peptide_id=7739&charge=3&scanf=16751&mz_l=1533.916&mz_h=1733.916&show_mass_error=1&match_percentile=35&precision=3&soleil_threshold=0&soleil_sort=m/z&opt_show_bion_1=1&opt_label_bion_1=1&opt_show_yion_1=1&opt_label_yion_1=1&opt_show_bion_2=1&opt_label_bion_2=1&opt_show_yion_2=1&opt_label_yion_2=1&opt_show_pre=1&opt_only_matches=1&opt_label_pre=0&opt_show_soleil=0&opt_pin_soleil=1&old_zoomlevel=0&zoomlevel=3&old_izoomlevel=0&izoomlevel=0&label_method=0&peak_font_size=&action=panright) \| **---** \| **16** \| \| **F** \| **8** \| **-0.420** \| [**867.337**](https://tmsf.med.harvard.edu/core/www/modules/sdig/index.php?value=14/84&pvkey=&run_id=92258&search_id=93772&scans_id=92254&peptide_id=7739&charge=3&scanf=16751&mz_l=767.337&mz_h=967.337&show_mass_error=1&match_percentile=35&precision=3&soleil_threshold=0&soleil_sort=m/z&opt_show_bion_1=1&opt_label_bion_1=1&opt_show_yion_1=1&opt_label_yion_1=1&opt_show_bion_2=1&opt_label_bion_2=1&opt_show_yion_2=1&opt_label_yion_2=1&opt_show_pre=1&opt_only_matches=1&opt_label_pre=0&opt_show_soleil=0&opt_pin_soleil=1&old_zoomlevel=0&zoomlevel=3&old_izoomlevel=0&izoomlevel=0&label_method=0&peak_font_size=&action=panright) \| [1532.868](https://tmsf.med.harvard.edu/core/www/modules/sdig/index.php?value=14/84&pvkey=&run_id=92258&search_id=93772&scans_id=92254&peptide_id=7739&charge=3&scanf=16751&mz_l=1432.868&mz_h=1632.868&show_mass_error=1&match_percentile=35&precision=3&soleil_threshold=0&soleil_sort=m/z&opt_show_bion_1=1&opt_label_bion_1=1&opt_show_yion_1=1&opt_label_yion_1=1&opt_show_bion_2=1&opt_label_bion_2=1&opt_show_yion_2=1&opt_label_yion_2=1&opt_show_pre=1&opt_only_matches=1&opt_label_pre=0&opt_show_soleil=0&opt_pin_soleil=1&old_zoomlevel=0&zoomlevel=3&old_izoomlevel=0&izoomlevel=0&label_method=0&peak_font_size=&action=panright) \| **---** \| **15** \| \| **I** \| **9** \| **-4.251** \| [**980.421**](https://tmsf.med.harvard.edu/core/www/modules/sdig/index.php?value=14/84&pvkey=&run_id=92258&search_id=93772&scans_id=92254&peptide_id=7739&charge=3&scanf=16751&mz_l=880.421&mz_h=1080.421&show_mass_error=1&match_percentile=35&precision=3&soleil_threshold=0&soleil_sort=m/z&opt_show_bion_1=1&opt_label_bion_1=1&opt_show_yion_1=1&opt_label_yion_1=1&opt_show_bion_2=1&opt_label_bion_2=1&opt_show_yion_2=1&opt_label_yion_2=1&opt_show_pre=1&opt_only_matches=1&opt_label_pre=0&opt_show_soleil=0&opt_pin_soleil=1&old_zoomlevel=0&zoomlevel=3&old_izoomlevel=0&izoomlevel=0&label_method=0&peak_font_size=&action=panright) \| [1385.800](https://tmsf.med.harvard.edu/core/www/modules/sdig/index.php?value=14/84&pvkey=&run_id=92258&search_id=93772&scans_id=92254&peptide_id=7739&charge=3&scanf=16751&mz_l=1285.8&mz_h=1485.8&show_mass_error=1&match_percentile=35&precision=3&soleil_threshold=0&soleil_sort=m/z&opt_show_bion_1=1&opt_label_bion_1=1&opt_show_yion_1=1&opt_label_yion_1=1&opt_show_bion_2=1&opt_label_bion_2=1&opt_show_yion_2=1&opt_label_yion_2=1&opt_show_pre=1&opt_only_matches=1&opt_label_pre=0&opt_show_soleil=0&opt_pin_soleil=1&old_zoomlevel=0&zoomlevel=3&old_izoomlevel=0&izoomlevel=0&label_method=0&peak_font_size=&action=panright) \| **---** \| **14** \| \| **A** \| **10** \| **-5.303** \| [**1051.458**](https://tmsf.med.harvard.edu/core/www/modules/sdig/index.php?value=14/84&pvkey=&run_id=92258&search_id=93772&scans_id=92254&peptide_id=7739&charge=3&scanf=16751&mz_l=951.458&mz_h=1151.458&show_mass_error=1&match_percentile=35&precision=3&soleil_threshold=0&soleil_sort=m/z&opt_show_bion_1=1&opt_label_bion_1=1&opt_show_yion_1=1&opt_label_yion_1=1&opt_show_bion_2=1&opt_label_bion_2=1&opt_show_yion_2=1&opt_label_yion_2=1&opt_show_pre=1&opt_only_matches=1&opt_label_pre=0&opt_show_soleil=0&opt_pin_soleil=1&old_zoomlevel=0&zoomlevel=3&old_izoomlevel=0&izoomlevel=0&label_method=0&peak_font_size=&action=panright) \| [**1272.716**](https://tmsf.med.harvard.edu/core/www/modules/sdig/index.php?value=14/84&pvkey=&run_id=92258&search_id=93772&scans_id=92254&peptide_id=7739&charge=3&scanf=16751&mz_l=1172.716&mz_h=1372.716&show_mass_error=1&match_percentile=35&precision=3&soleil_threshold=0&soleil_sort=m/z&opt_show_bion_1=1&opt_label_bion_1=1&opt_show_yion_1=1&opt_label_yion_1=1&opt_show_bion_2=1&opt_label_bion_2=1&opt_show_yion_2=1&opt_label_yion_2=1&opt_show_pre=1&opt_only_matches=1&opt_label_pre=0&opt_show_soleil=0&opt_pin_soleil=1&old_zoomlevel=0&zoomlevel=3&old_izoomlevel=0&izoomlevel=0&label_method=0&peak_font_size=&action=panright) \| **-0.938** \| **13** \| \| **D** \| **11** \| **-1.506** \| [**1166.485**](https://tmsf.med.harvard.edu/core/www/modules/sdig/index.php?value=14/84&pvkey=&run_id=92258&search_id=93772&scans_id=92254&peptide_id=7739&charge=3&scanf=16751&mz_l=1066.485&mz_h=1266.485&show_mass_error=1&match_percentile=35&precision=3&soleil_threshold=0&soleil_sort=m/z&opt_show_bion_1=1&opt_label_bion_1=1&opt_show_yion_1=1&opt_label_yion_1=1&opt_show_bion_2=1&opt_label_bion_2=1&opt_show_yion_2=1&opt_label_yion_2=1&opt_show_pre=1&opt_only_matches=1&opt_label_pre=0&opt_show_soleil=0&opt_pin_soleil=1&old_zoomlevel=0&zoomlevel=3&old_izoomlevel=0&izoomlevel=0&label_method=0&peak_font_size=&action=panright) \| [**1201.679**](https://tmsf.med.harvard.edu/core/www/modules/sdig/index.php?value=14/84&pvkey=&run_id=92258&search_id=93772&scans_id=92254&peptide_id=7739&charge=3&scanf=16751&mz_l=1101.679&mz_h=1301.679&show_mass_error=1&match_percentile=35&precision=3&soleil_threshold=0&soleil_sort=m/z&opt_show_bion_1=1&opt_label_bion_1=1&opt_show_yion_1=1&opt_label_yion_1=1&opt_show_bion_2=1&opt_label_bion_2=1&opt_show_yion_2=1&opt_label_yion_2=1&opt_show_pre=1&opt_only_matches=1&opt_label_pre=0&opt_show_soleil=0&opt_pin_soleil=1&old_zoomlevel=0&zoomlevel=3&old_izoomlevel=0&izoomlevel=0&label_method=0&peak_font_size=&action=panright) \| **-0.278** \| **12** \| \| **L** \| **12** \| **---** \| [1279.569](https://tmsf.med.harvard.edu/core/www/modules/sdig/index.php?value=14/84&pvkey=&run_id=92258&search_id=93772&scans_id=92254&peptide_id=7739&charge=3&scanf=16751&mz_l=1179.569&mz_h=1379.569&show_mass_error=1&match_percentile=35&precision=3&soleil_threshold=0&soleil_sort=m/z&opt_show_bion_1=1&opt_label_bion_1=1&opt_show_yion_1=1&opt_label_yion_1=1&opt_show_bion_2=1&opt_label_bion_2=1&opt_show_yion_2=1&opt_label_yion_2=1&opt_show_pre=1&opt_only_matches=1&opt_label_pre=0&opt_show_soleil=0&opt_pin_soleil=1&old_zoomlevel=0&zoomlevel=3&old_izoomlevel=0&izoomlevel=0&label_method=0&peak_font_size=&action=panright) \| [1086.652](https://tmsf.med.harvard.edu/core/www/modules/sdig/index.php?value=14/84&pvkey=&run_id=92258&search_id=93772&scans_id=92254&peptide_id=7739&charge=3&scanf=16751&mz_l=986.652&mz_h=1186.652&show_mass_error=1&match_percentile=35&precision=3&soleil_threshold=0&soleil_sort=m/z&opt_show_bion_1=1&opt_label_bion_1=1&opt_show_yion_1=1&opt_label_yion_1=1&opt_show_bion_2=1&opt_label_bion_2=1&opt_show_yion_2=1&opt_label_yion_2=1&opt_show_pre=1&opt_only_matches=1&opt_label_pre=0&opt_show_soleil=0&opt_pin_soleil=1&old_zoomlevel=0&zoomlevel=3&old_izoomlevel=0&izoomlevel=0&label_method=0&peak_font_size=&action=panright) \| **---** \| **11** \| \| **S** \| **13** \| **---** \| [1366.601](https://tmsf.med.harvard.edu/core/www/modules/sdig/index.php?value=14/84&pvkey=&run_id=92258&search_id=93772&scans_id=92254&peptide_id=7739&charge=3&scanf=16751&mz_l=1266.601&mz_h=1466.601&show_mass_error=1&match_percentile=35&precision=3&soleil_threshold=0&soleil_sort=m/z&opt_show_bion_1=1&opt_label_bion_1=1&opt_show_yion_1=1&opt_label_yion_1=1&opt_show_bion_2=1&opt_label_bion_2=1&opt_show_yion_2=1&opt_label_yion_2=1&opt_show_pre=1&opt_only_matches=1&opt_label_pre=0&opt_show_soleil=0&opt_pin_soleil=1&old_zoomlevel=0&zoomlevel=3&old_izoomlevel=0&izoomlevel=0&label_method=0&peak_font_size=&action=panright) \| [**973.568**](https://tmsf.med.harvard.edu/core/www/modules/sdig/index.php?value=14/84&pvkey=&run_id=92258&search_id=93772&scans_id=92254&peptide_id=7739&charge=3&scanf=16751&mz_l=873.568&mz_h=1073.568&show_mass_error=1&match_percentile=35&precision=3&soleil_threshold=0&soleil_sort=m/z&opt_show_bion_1=1&opt_label_bion_1=1&opt_show_yion_1=1&opt_label_yion_1=1&opt_show_bion_2=1&opt_label_bion_2=1&opt_show_yion_2=1&opt_label_yion_2=1&opt_show_pre=1&opt_only_matches=1&opt_label_pre=0&opt_show_soleil=0&opt_pin_soleil=1&old_zoomlevel=0&zoomlevel=3&old_izoomlevel=0&izoomlevel=0&label_method=0&peak_font_size=&action=panright) \| **1.333** \| **10** \| \| **V** \| **14** \| **---** \| [1465.669](https://tmsf.med.harvard.edu/core/www/modules/sdig/index.php?value=14/84&pvkey=&run_id=92258&search_id=93772&scans_id=92254&peptide_id=7739&charge=3&scanf=16751&mz_l=1365.669&mz_h=1565.669&show_mass_error=1&match_percentile=35&precision=3&soleil_threshold=0&soleil_sort=m/z&opt_show_bion_1=1&opt_label_bion_1=1&opt_show_yion_1=1&opt_label_yion_1=1&opt_show_bion_2=1&opt_label_bion_2=1&opt_show_yion_2=1&opt_label_yion_2=1&opt_show_pre=1&opt_only_matches=1&opt_label_pre=0&opt_show_soleil=0&opt_pin_soleil=1&old_zoomlevel=0&zoomlevel=3&old_izoomlevel=0&izoomlevel=0&label_method=0&peak_font_size=&action=panright) \| [**886.536**](https://tmsf.med.harvard.edu/core/www/modules/sdig/index.php?value=14/84&pvkey=&run_id=92258&search_id=93772&scans_id=92254&peptide_id=7739&charge=3&scanf=16751&mz_l=786.536&mz_h=986.536&show_mass_error=1&match_percentile=35&precision=3&soleil_threshold=0&soleil_sort=m/z&opt_show_bion_1=1&opt_label_bion_1=1&opt_show_yion_1=1&opt_label_yion_1=1&opt_show_bion_2=1&opt_label_bion_2=1&opt_show_yion_2=1&opt_label_yion_2=1&opt_show_pre=1&opt_only_matches=1&opt_label_pre=0&opt_show_soleil=0&opt_pin_soleil=1&old_zoomlevel=0&zoomlevel=3&old_izoomlevel=0&izoomlevel=0&label_method=0&peak_font_size=&action=panright) \| **3.512** \| **9** \| \| **G** \| **15** \| **---** \| [1522.691](https://tmsf.med.harvard.edu/core/www/modules/sdig/index.php?value=14/84&pvkey=&run_id=92258&search_id=93772&scans_id=92254&peptide_id=7739&charge=3&scanf=16751&mz_l=1422.691&mz_h=1622.691&show_mass_error=1&match_percentile=35&precision=3&soleil_threshold=0&soleil_sort=m/z&opt_show_bion_1=1&opt_label_bion_1=1&opt_show_yion_1=1&opt_label_yion_1=1&opt_show_bion_2=1&opt_label_bion_2=1&opt_show_yion_2=1&opt_label_yion_2=1&opt_show_pre=1&opt_only_matches=1&opt_label_pre=0&opt_show_soleil=0&opt_pin_soleil=1&old_zoomlevel=0&zoomlevel=3&old_izoomlevel=0&izoomlevel=0&label_method=0&peak_font_size=&action=panright) \| [**787.467**](https://tmsf.med.harvard.edu/core/www/modules/sdig/index.php?value=14/84&pvkey=&run_id=92258&search_id=93772&scans_id=92254&peptide_id=7739&charge=3&scanf=16751&mz_l=687.467&mz_h=887.467&show_mass_error=1&match_percentile=35&precision=3&soleil_threshold=0&soleil_sort=m/z&opt_show_bion_1=1&opt_label_bion_1=1&opt_show_yion_1=1&opt_label_yion_1=1&opt_show_bion_2=1&opt_label_bion_2=1&opt_show_yion_2=1&opt_label_yion_2=1&opt_show_pre=1&opt_only_matches=1&opt_label_pre=0&opt_show_soleil=0&opt_pin_soleil=1&old_zoomlevel=0&zoomlevel=3&old_izoomlevel=0&izoomlevel=0&label_method=0&peak_font_size=&action=panright) \| **-0.395** \| **8** \| \| **L** \| **16** \| **---** \| [1635.775](https://tmsf.med.harvard.edu/core/www/modules/sdig/index.php?value=14/84&pvkey=&run_id=92258&search_id=93772&scans_id=92254&peptide_id=7739&charge=3&scanf=16751&mz_l=1535.775&mz_h=1735.775&show_mass_error=1&match_percentile=35&precision=3&soleil_threshold=0&soleil_sort=m/z&opt_show_bion_1=1&opt_label_bion_1=1&opt_show_yion_1=1&opt_label_yion_1=1&opt_show_bion_2=1&opt_label_bion_2=1&opt_show_yion_2=1&opt_label_yion_2=1&opt_show_pre=1&opt_only_matches=1&opt_label_pre=0&opt_show_soleil=0&opt_pin_soleil=1&old_zoomlevel=0&zoomlevel=3&old_izoomlevel=0&izoomlevel=0&label_method=0&peak_font_size=&action=panright) \| [**730.446**](https://tmsf.med.harvard.edu/core/www/modules/sdig/index.php?value=14/84&pvkey=&run_id=92258&search_id=93772&scans_id=92254&peptide_id=7739&charge=3&scanf=16751&mz_l=630.446&mz_h=830.446&show_mass_error=1&match_percentile=35&precision=3&soleil_threshold=0&soleil_sort=m/z&opt_show_bion_1=1&opt_label_bion_1=1&opt_show_yion_1=1&opt_label_yion_1=1&opt_show_bion_2=1&opt_label_bion_2=1&opt_show_yion_2=1&opt_label_yion_2=1&opt_show_pre=1&opt_only_matches=1&opt_label_pre=0&opt_show_soleil=0&opt_pin_soleil=1&old_zoomlevel=0&zoomlevel=3&old_izoomlevel=0&izoomlevel=0&label_method=0&peak_font_size=&action=panright) \| **3.390** \| **7** \| \| **A** \| **17** \| **---** \| [1706.812](https://tmsf.med.harvard.edu/core/www/modules/sdig/index.php?value=14/84&pvkey=&run_id=92258&search_id=93772&scans_id=92254&peptide_id=7739&charge=3&scanf=16751&mz_l=1606.812&mz_h=1806.812&show_mass_error=1&match_percentile=35&precision=3&soleil_threshold=0&soleil_sort=m/z&opt_show_bion_1=1&opt_label_bion_1=1&opt_show_yion_1=1&opt_label_yion_1=1&opt_show_bion_2=1&opt_label_bion_2=1&opt_show_yion_2=1&opt_label_yion_2=1&opt_show_pre=1&opt_only_matches=1&opt_label_pre=0&opt_show_soleil=0&opt_pin_soleil=1&old_zoomlevel=0&zoomlevel=3&old_izoomlevel=0&izoomlevel=0&label_method=0&peak_font_size=&action=panright) \| [**617.362**](https://tmsf.med.harvard.edu/core/www/modules/sdig/index.php?value=14/84&pvkey=&run_id=92258&search_id=93772&scans_id=92254&peptide_id=7739&charge=3&scanf=16751&mz_l=517.362&mz_h=717.362&show_mass_error=1&match_percentile=35&precision=3&soleil_threshold=0&soleil_sort=m/z&opt_show_bion_1=1&opt_label_bion_1=1&opt_show_yion_1=1&opt_label_yion_1=1&opt_show_bion_2=1&opt_label_bion_2=1&opt_show_yion_2=1&opt_label_yion_2=1&opt_show_pre=1&opt_only_matches=1&opt_label_pre=0&opt_show_soleil=0&opt_pin_soleil=1&old_zoomlevel=0&zoomlevel=3&old_izoomlevel=0&izoomlevel=0&label_method=0&peak_font_size=&action=panright) \| **1.372** \| **6** \| \| **T** \| **18** \| **---** \| [1807.860](https://tmsf.med.harvard.edu/core/www/modules/sdig/index.php?value=14/84&pvkey=&run_id=92258&search_id=93772&scans_id=92254&peptide_id=7739&charge=3&scanf=16751&mz_l=1707.86&mz_h=1907.86&show_mass_error=1&match_percentile=35&precision=3&soleil_threshold=0&soleil_sort=m/z&opt_show_bion_1=1&opt_label_bion_1=1&opt_show_yion_1=1&opt_label_yion_1=1&opt_show_bion_2=1&opt_label_bion_2=1&opt_show_yion_2=1&opt_label_yion_2=1&opt_show_pre=1&opt_only_matches=1&opt_label_pre=0&opt_show_soleil=0&opt_pin_soleil=1&old_zoomlevel=0&zoomlevel=3&old_izoomlevel=0&izoomlevel=0&label_method=0&peak_font_size=&action=panright) \| [**546.325**](https://tmsf.med.harvard.edu/core/www/modules/sdig/index.php?value=14/84&pvkey=&run_id=92258&search_id=93772&scans_id=92254&peptide_id=7739&charge=3&scanf=16751&mz_l=446.325&mz_h=646.325&show_mass_error=1&match_percentile=35&precision=3&soleil_threshold=0&soleil_sort=m/z&opt_show_bion_1=1&opt_label_bion_1=1&opt_show_yion_1=1&opt_label_yion_1=1&opt_show_bion_2=1&opt_label_bion_2=1&opt_show_yion_2=1&opt_label_yion_2=1&opt_show_pre=1&opt_only_matches=1&opt_label_pre=0&opt_show_soleil=0&opt_pin_soleil=1&old_zoomlevel=0&zoomlevel=3&old_izoomlevel=0&izoomlevel=0&label_method=0&peak_font_size=&action=panright) \| **-0.118** \| **5** \| \| **G** \| **19** \| **---** \| [1864.881](https://tmsf.med.harvard.edu/core/www/modules/sdig/index.php?value=14/84&pvkey=&run_id=92258&search_id=93772&scans_id=92254&peptide_id=7739&charge=3&scanf=16751&mz_l=1764.881&mz_h=1964.881&show_mass_error=1&match_percentile=35&precision=3&soleil_threshold=0&soleil_sort=m/z&opt_show_bion_1=1&opt_label_bion_1=1&opt_show_yion_1=1&opt_label_yion_1=1&opt_show_bion_2=1&opt_label_bion_2=1&opt_show_yion_2=1&opt_label_yion_2=1&opt_show_pre=1&opt_only_matches=1&opt_label_pre=0&opt_show_soleil=0&opt_pin_soleil=1&old_zoomlevel=0&zoomlevel=3&old_izoomlevel=0&izoomlevel=0&label_method=0&peak_font_size=&action=panright) \| [**445.277**](https://tmsf.med.harvard.edu/core/www/modules/sdig/index.php?value=14/84&pvkey=&run_id=92258&search_id=93772&scans_id=92254&peptide_id=7739&charge=3&scanf=16751&mz_l=345.277&mz_h=545.277&show_mass_error=1&match_percentile=35&precision=3&soleil_threshold=0&soleil_sort=m/z&opt_show_bion_1=1&opt_label_bion_1=1&opt_show_yion_1=1&opt_label_yion_1=1&opt_show_bion_2=1&opt_label_bion_2=1&opt_show_yion_2=1&opt_label_yion_2=1&opt_show_pre=1&opt_only_matches=1&opt_label_pre=0&opt_show_soleil=0&opt_pin_soleil=1&old_zoomlevel=0&zoomlevel=3&old_izoomlevel=0&izoomlevel=0&label_method=0&peak_font_size=&action=panright) \| **0.152** \| **4** \| \| **Q** \| **20** \| **---** \| [1992.940](https://tmsf.med.harvard.edu/core/www/modules/sdig/index.php?value=14/84&pvkey=&run_id=92258&search_id=93772&scans_id=92254&peptide_id=7739&charge=3&scanf=16751&mz_l=1892.94&mz_h=2092.94&show_mass_error=1&match_percentile=35&precision=3&soleil_threshold=0&soleil_sort=m/z&opt_show_bion_1=1&opt_label_bion_1=1&opt_show_yion_1=1&opt_label_yion_1=1&opt_show_bion_2=1&opt_label_bion_2=1&opt_show_yion_2=1&opt_label_yion_2=1&opt_show_pre=1&opt_only_matches=1&opt_label_pre=0&opt_show_soleil=0&opt_pin_soleil=1&old_zoomlevel=0&zoomlevel=3&old_izoomlevel=0&izoomlevel=0&label_method=0&peak_font_size=&action=panright) \| [388.255](https://tmsf.med.harvard.edu/core/www/modules/sdig/index.php?value=14/84&pvkey=&run_id=92258&search_id=93772&scans_id=92254&peptide_id=7739&charge=3&scanf=16751&mz_l=288.255&mz_h=488.255&show_mass_error=1&match_percentile=35&precision=3&soleil_threshold=0&soleil_sort=m/z&opt_show_bion_1=1&opt_label_bion_1=1&opt_show_yion_1=1&opt_label_yion_1=1&opt_show_bion_2=1&opt_label_bion_2=1&opt_show_yion_2=1&opt_label_yion_2=1&opt_show_pre=1&opt_only_matches=1&opt_label_pre=0&opt_show_soleil=0&opt_pin_soleil=1&old_zoomlevel=0&zoomlevel=3&old_izoomlevel=0&izoomlevel=0&label_method=0&peak_font_size=&action=panright) \| **---** \| **3** \| \| **I** \| **21** \| **---** \| [2106.024](https://tmsf.med.harvard.edu/core/www/modules/sdig/index.php?value=14/84&pvkey=&run_id=92258&search_id=93772&scans_id=92254&peptide_id=7739&charge=3&scanf=16751&mz_l=2006.024&mz_h=2206.024&show_mass_error=1&match_percentile=35&precision=3&soleil_threshold=0&soleil_sort=m/z&opt_show_bion_1=1&opt_label_bion_1=1&opt_show_yion_1=1&opt_label_yion_1=1&opt_show_bion_2=1&opt_label_bion_2=1&opt_show_yion_2=1&opt_label_yion_2=1&opt_show_pre=1&opt_only_matches=1&opt_label_pre=0&opt_show_soleil=0&opt_pin_soleil=1&old_zoomlevel=0&zoomlevel=3&old_izoomlevel=0&izoomlevel=0&label_method=0&peak_font_size=&action=panright) \| [**260.197**](https://tmsf.med.harvard.edu/core/www/modules/sdig/index.php?value=14/84&pvkey=&run_id=92258&search_id=93772&scans_id=92254&peptide_id=7739&charge=3&scanf=16751&mz_l=160.197&mz_h=360.197&show_mass_error=1&match_percentile=35&precision=3&soleil_threshold=0&soleil_sort=m/z&opt_show_bion_1=1&opt_label_bion_1=1&opt_show_yion_1=1&opt_label_yion_1=1&opt_show_bion_2=1&opt_label_bion_2=1&opt_show_yion_2=1&opt_label_yion_2=1&opt_show_pre=1&opt_only_matches=1&opt_label_pre=0&opt_show_soleil=0&opt_pin_soleil=1&old_zoomlevel=0&zoomlevel=3&old_izoomlevel=0&izoomlevel=0&label_method=0&peak_font_size=&action=panright) \| **1.409** \| **2** \| \| **K** \| **22** \| **---** \| --- \| [**147.113**](https://tmsf.med.harvard.edu/core/www/modules/sdig/index.php?value=14/84&pvkey=&run_id=92258&search_id=93772&scans_id=92254&peptide_id=7739&charge=3&scanf=16751&mz_l=47.113&mz_h=247.113&show_mass_error=1&match_percentile=35&precision=3&soleil_threshold=0&soleil_sort=m/z&opt_show_bion_1=1&opt_label_bion_1=1&opt_show_yion_1=1&opt_label_yion_1=1&opt_show_bion_2=1&opt_label_bion_2=1&opt_show_yion_2=1&opt_label_yion_2=1&opt_show_pre=1&opt_only_matches=1&opt_label_pre=0&opt_show_soleil=0&opt_pin_soleil=1&old_zoomlevel=0&zoomlevel=3&old_izoomlevel=0&izoomlevel=0&label_method=0&peak_font_size=&action=panright) \| **-0.286** \| **1** \| |

**Sequence no 6**

| [>tr\|A0A1U8IW55\|A0A1U8IW55_GOSHI Fructose-bisphosphate aldolase OS=Gossypium hirsutum OX=3635 GN=LOC107899428 PE=3 SV=1](http://www.uniprot.org/uniprot/?query=A0A1U8IW55_GOSHI) |
| --- |
| MSCFISKYAD ELAKNAAYIG TPGKGILAAD ESTGTIGKRL SSINVENVED NRRALRELLF TTPGALQYLS GVILFEETLY QKTASGKPFV DVLKEGGVLP  GIKVDKGTVE LNGTNGETFT QGLDGLAQRC QKYYEAGARF AKWRAVLKIS ANEPTELAIQ ENANGLAMYA AICQQCGLVP IVEPEILVDG SHDIKKCAAV  TERVLAACYK ALNDHHVMLE GTLLKPNMVT PGSDSPKVAP EVIAEYTVRA LQRTVPAAVP AIVFLSGGQS EEEATLNLNA MNKLQTKKPW SLSFSFGRAL  QQSTLKAWAG KEENVKKAQD AFLVRCKANS EATLGTYKGD AKISEGAAES LHVKDYKY |

| Legend | | |
| --- | --- | --- |
| THISREFERE NCE | UNMATCHED | OTHERREFER ENCES |

| \| 38629.94 Da \| Protein Coverage \| \| \| --- \| --- \| --- \| \| 358 AA \| 12 AA \| **3.35 %** \| |
| --- | --- | --- | --- | --- | --- | --- |

**Data table 11**

| \| **Sequence** \| \| --- \| \| VAPEVIAEYTVR \| |
| --- | --- | --- |
| **Predicted Fragmentation Pattern**   \| **Seq** \| **#** \| **b: Δ Error** \| **b** \| **y** \| **y: Δ Error** \| **+1** \| \| --- \| --- \| --- \| --- \| --- \| --- \| --- \| \| **V** \| **1** \| **---** \| [100.076](https://tmsf.med.harvard.edu/core/www/modules/sdig/index.php?value=11/22&pvkey=&run_id=92259&search_id=93773&scans_id=92255&peptide_id=2637&charge=2&scanf=11765&mz_l=0.075999999999993&mz_h=200.076) \| --- \| **---** \| **12** \| \| **A** \| **2** \| **-0.687** \| [**171.113**](https://tmsf.med.harvard.edu/core/www/modules/sdig/index.php?value=11/22&pvkey=&run_id=92259&search_id=93773&scans_id=92255&peptide_id=2637&charge=2&scanf=11765&mz_l=71.113&mz_h=271.113) \| [**1247.663**](https://tmsf.med.harvard.edu/core/www/modules/sdig/index.php?value=11/22&pvkey=&run_id=92259&search_id=93773&scans_id=92255&peptide_id=2637&charge=2&scanf=11765&mz_l=1147.663&mz_h=1347.663) \| **0.437** \| **11** \| \| **P** \| **3** \| **5.426** \| [**268.166**](https://tmsf.med.harvard.edu/core/www/modules/sdig/index.php?value=11/22&pvkey=&run_id=92259&search_id=93773&scans_id=92255&peptide_id=2637&charge=2&scanf=11765&mz_l=168.166&mz_h=368.166) \| [**1176.626**](https://tmsf.med.harvard.edu/core/www/modules/sdig/index.php?value=11/22&pvkey=&run_id=92259&search_id=93773&scans_id=92255&peptide_id=2637&charge=2&scanf=11765&mz_l=1076.626&mz_h=1276.626) \| **9.285** \| **10** \| \| **E** \| **4** \| **2.458** \| [**397.208**](https://tmsf.med.harvard.edu/core/www/modules/sdig/index.php?value=11/22&pvkey=&run_id=92259&search_id=93773&scans_id=92255&peptide_id=2637&charge=2&scanf=11765&mz_l=297.208&mz_h=497.208) \| [**1079.573**](https://tmsf.med.harvard.edu/core/www/modules/sdig/index.php?value=11/22&pvkey=&run_id=92259&search_id=93773&scans_id=92255&peptide_id=2637&charge=2&scanf=11765&mz_l=979.573&mz_h=1179.573) \| **-0.820** \| **9** \| \| **V** \| **5** \| **0.812** \| [**496.277**](https://tmsf.med.harvard.edu/core/www/modules/sdig/index.php?value=11/22&pvkey=&run_id=92259&search_id=93773&scans_id=92255&peptide_id=2637&charge=2&scanf=11765&mz_l=396.277&mz_h=596.277) \| [**950.531**](https://tmsf.med.harvard.edu/core/www/modules/sdig/index.php?value=11/22&pvkey=&run_id=92259&search_id=93773&scans_id=92255&peptide_id=2637&charge=2&scanf=11765&mz_l=850.531&mz_h=1050.531) \| **0.150** \| **8** \| \| **I** \| **6** \| **1.733** \| [**609.361**](https://tmsf.med.harvard.edu/core/www/modules/sdig/index.php?value=11/22&pvkey=&run_id=92259&search_id=93773&scans_id=92255&peptide_id=2637&charge=2&scanf=11765&mz_l=509.361&mz_h=709.361) \| [**851.462**](https://tmsf.med.harvard.edu/core/www/modules/sdig/index.php?value=11/22&pvkey=&run_id=92259&search_id=93773&scans_id=92255&peptide_id=2637&charge=2&scanf=11765&mz_l=751.462&mz_h=951.462) \| **-0.629** \| **7** \| \| **A** \| **7** \| **---** \| [680.398](https://tmsf.med.harvard.edu/core/www/modules/sdig/index.php?value=11/22&pvkey=&run_id=92259&search_id=93773&scans_id=92255&peptide_id=2637&charge=2&scanf=11765&mz_l=580.398&mz_h=780.398) \| [**738.378**](https://tmsf.med.harvard.edu/core/www/modules/sdig/index.php?value=11/22&pvkey=&run_id=92259&search_id=93773&scans_id=92255&peptide_id=2637&charge=2&scanf=11765&mz_l=638.378&mz_h=838.378) \| **-0.452** \| **6** \| \| **E** \| **8** \| **---** \| [809.440](https://tmsf.med.harvard.edu/core/www/modules/sdig/index.php?value=11/22&pvkey=&run_id=92259&search_id=93773&scans_id=92255&peptide_id=2637&charge=2&scanf=11765&mz_l=709.44&mz_h=909.44) \| [**667.341**](https://tmsf.med.harvard.edu/core/www/modules/sdig/index.php?value=11/22&pvkey=&run_id=92259&search_id=93773&scans_id=92255&peptide_id=2637&charge=2&scanf=11765&mz_l=567.341&mz_h=767.341) \| **0.055** \| **5** \| \| **Y** \| **9** \| **-14.761** \| [**972.504**](https://tmsf.med.harvard.edu/core/www/modules/sdig/index.php?value=11/22&pvkey=&run_id=92259&search_id=93773&scans_id=92255&peptide_id=2637&charge=2&scanf=11765&mz_l=872.504&mz_h=1072.504) \| [**538.298**](https://tmsf.med.harvard.edu/core/www/modules/sdig/index.php?value=11/22&pvkey=&run_id=92259&search_id=93773&scans_id=92255&peptide_id=2637&charge=2&scanf=11765&mz_l=438.298&mz_h=638.298) \| **-2.103** \| **4** \| \| **T** \| **10** \| **---** \| [1073.551](https://tmsf.med.harvard.edu/core/www/modules/sdig/index.php?value=11/22&pvkey=&run_id=92259&search_id=93773&scans_id=92255&peptide_id=2637&charge=2&scanf=11765&mz_l=973.551&mz_h=1173.551) \| [**375.235**](https://tmsf.med.harvard.edu/core/www/modules/sdig/index.php?value=11/22&pvkey=&run_id=92259&search_id=93773&scans_id=92255&peptide_id=2637&charge=2&scanf=11765&mz_l=275.235&mz_h=475.235) \| **-0.809** \| **3** \| \| **V** \| **11** \| **---** \| [1172.620](https://tmsf.med.harvard.edu/core/www/modules/sdig/index.php?value=11/22&pvkey=&run_id=92259&search_id=93773&scans_id=92255&peptide_id=2637&charge=2&scanf=11765&mz_l=1072.62&mz_h=1272.62) \| [**274.187**](https://tmsf.med.harvard.edu/core/www/modules/sdig/index.php?value=11/22&pvkey=&run_id=92259&search_id=93773&scans_id=92255&peptide_id=2637&charge=2&scanf=11765&mz_l=174.187&mz_h=374.187) \| **-0.181** \| **2** \| \| **R** \| **12** \| **---** \| --- \| [**175.119**](https://tmsf.med.harvard.edu/core/www/modules/sdig/index.php?value=11/22&pvkey=&run_id=92259&search_id=93773&scans_id=92255&peptide_id=2637&charge=2&scanf=11765&mz_l=75.119&mz_h=275.119) \| **-0.756** \| **1** \| \|  \|  \|  \|  \|  \|  \|  \| |

**Sequence no 7**

| [>tr\|A0A1U8HQ71\|A0A1U8HQ71_GOSHI Alpha-1,4 glucan phosphorylase OS=Gossypium hirsutum OX=3635 GN=LOC107888550 PE=3 SV=1](http://www.uniprot.org/uniprot/?query=A0A1U8HQ71_GOSHI) |
| --- |
| MATWRYSTMP GGAEAVSSRN AVGRFVDFSH GGGGGRRGIK PDQLMLIRKW QLRPVKRSFS VRNVSSEQQQ KVQDLVTQQQ ESPGSYNPFP PDASSIASSI  KYHSEFTPLF SPEKFDPPKA FFATAQSIRD ALIINWNATY DYYERLNVKQ AYYLSMEFLQ GRALLNAIGN LGLTGAYAEA LSKLGHNLEN IASQEPDAAL  GNGGLGRLAS CFLDSLATLN YPAWGYGLRY RYGLFKQRIT KDGQEEVAEN WLEMSNPWEI VRNDVVYPIK FYGKVLTDSD GKKHWTGGED IQAVAYDVPI  PGYETKTTIN LRLWSTKAPS GDFDLSVFNS GKHTQAAEAL YTAEKICYVL YPGDESLEGK ILRLKQQYTL CSASLQDIIA RFERRSGAKV KWDEFPDKVA  VQMNDTHPTL CIPELMRILI DVKGLSWKEA WNITQRTVAY TNHTVLPEAL EKWSLELMQK LLPRHMEIIE MIDEELIRTI VSEHGNADSN LLEKKLKQMR  ILENVELPAA FSDLLVKPKK SLVAVPSDEL GESEEEEEEE EEEEEEEAEA EAEEEEEEEK LKPAGGKIKS IKEGTQGKKK KIPEPVPEPP KLVRMANLCV  VGGHAVNGVA AIHSEIVKDE VFNDFFQLWP EKFQNKTNGV TPRRWIRFCN PELSNIITSW TGSEDWVLNT EKLSELRKFA DNEDLQIQWR AAKRSNKLKV  ASLIKERTGY IVSPDSMFDI QVKRIHEYKR QLLNILGIVY RYKKMKEMSA SERKKKFVPR VCIFGGKAFA TYVQAKRIVK FITDVGATVN HDPDIGDLLK  VIFVPDYNVN VAELLIPASE LSQHISTAGM EASGTSNMKF AMNGCILIGT LDGANVEIRE EVGEENFFLF GAKAHEIAGL RKERAEGKFV PDPQFEEVKK  FIKSGVFGSS NYNELLGSLE GNEGFGRADY FLVGKDFPSY IECQEKVDET YKDQKVWTRM SIMNTAGSYN FSSDRTIHEY AREIWNIKPV ELP |

| Legend | | |
| --- | --- | --- |
| THISREFERE NCE | UNMATCHED | OTHERREFER ENCES |

| \| 112263.19 Da \| Protein Coverage \| \| \| --- \| --- \| --- \| \| 993 AA \| 11 AA \| **1.11 %** \| |
| --- | --- | --- | --- | --- | --- | --- |

**Data table 12**

| \| **Sequence** \| \| --- \| \| QLLNILGIVYR \| |
| --- | --- | --- |
| **Predicted Fragmentation Pattern**   \| **Seq** \| **#** \| **b: Δ Error** \| **b** \| **y** \| **y: Δ Error** \| **+1** \| \| --- \| --- \| --- \| --- \| --- \| --- \| --- \| \| **Q** \| **1** \| **-0.565** \| [**129.066**](https://tmsf.med.harvard.edu/core/www/modules/sdig/index.php?value=11/20&pvkey=&run_id=92259&search_id=93773&scans_id=92255&peptide_id=8259&charge=2&scanf=17257&mz_l=29.066&mz_h=229.066) \| --- \| **---** \| **11** \| \| **L** \| **2** \| **-0.063** \| [**242.150**](https://tmsf.med.harvard.edu/core/www/modules/sdig/index.php?value=11/20&pvkey=&run_id=92259&search_id=93773&scans_id=92255&peptide_id=8259&charge=2&scanf=17257&mz_l=142.15&mz_h=342.15) \| [**1173.735**](https://tmsf.med.harvard.edu/core/www/modules/sdig/index.php?value=11/20&pvkey=&run_id=92259&search_id=93773&scans_id=92255&peptide_id=8259&charge=2&scanf=17257&mz_l=1073.735&mz_h=1273.735) \| **2.450** \| **10** \| \| **L** \| **3** \| **-0.353** \| [**355.234**](https://tmsf.med.harvard.edu/core/www/modules/sdig/index.php?value=11/20&pvkey=&run_id=92259&search_id=93773&scans_id=92255&peptide_id=8259&charge=2&scanf=17257&mz_l=255.234&mz_h=455.234) \| [**1060.651**](https://tmsf.med.harvard.edu/core/www/modules/sdig/index.php?value=11/20&pvkey=&run_id=92259&search_id=93773&scans_id=92255&peptide_id=8259&charge=2&scanf=17257&mz_l=960.651&mz_h=1160.651) \| **-0.437** \| **9** \| \| **N** \| **4** \| **-1.740** \| [**469.277**](https://tmsf.med.harvard.edu/core/www/modules/sdig/index.php?value=11/20&pvkey=&run_id=92259&search_id=93773&scans_id=92255&peptide_id=8259&charge=2&scanf=17257&mz_l=369.277&mz_h=569.277) \| [**947.567**](https://tmsf.med.harvard.edu/core/www/modules/sdig/index.php?value=11/20&pvkey=&run_id=92259&search_id=93773&scans_id=92255&peptide_id=8259&charge=2&scanf=17257&mz_l=847.567&mz_h=1047.567) \| **-0.727** \| **8** \| \| **I** \| **5** \| **---** \| [582.361](https://tmsf.med.harvard.edu/core/www/modules/sdig/index.php?value=11/20&pvkey=&run_id=92259&search_id=93773&scans_id=92255&peptide_id=8259&charge=2&scanf=17257&mz_l=482.361&mz_h=682.361) \| [**833.524**](https://tmsf.med.harvard.edu/core/www/modules/sdig/index.php?value=11/20&pvkey=&run_id=92259&search_id=93773&scans_id=92255&peptide_id=8259&charge=2&scanf=17257&mz_l=733.524&mz_h=933.524) \| **-0.363** \| **7** \| \| **L** \| **6** \| **---** \| [695.445](https://tmsf.med.harvard.edu/core/www/modules/sdig/index.php?value=11/20&pvkey=&run_id=92259&search_id=93773&scans_id=92255&peptide_id=8259&charge=2&scanf=17257&mz_l=595.445&mz_h=795.445) \| [**720.440**](https://tmsf.med.harvard.edu/core/www/modules/sdig/index.php?value=11/20&pvkey=&run_id=92259&search_id=93773&scans_id=92255&peptide_id=8259&charge=2&scanf=17257&mz_l=620.44&mz_h=820.44) \| **-0.818** \| **6** \| \| **G** \| **7** \| **---** \| [752.467](https://tmsf.med.harvard.edu/core/www/modules/sdig/index.php?value=11/20&pvkey=&run_id=92259&search_id=93773&scans_id=92255&peptide_id=8259&charge=2&scanf=17257&mz_l=652.467&mz_h=852.467) \| [**607.356**](https://tmsf.med.harvard.edu/core/www/modules/sdig/index.php?value=11/20&pvkey=&run_id=92259&search_id=93773&scans_id=92255&peptide_id=8259&charge=2&scanf=17257&mz_l=507.356&mz_h=707.356) \| **0.166** \| **5** \| \| **I** \| **8** \| **---** \| [865.551](https://tmsf.med.harvard.edu/core/www/modules/sdig/index.php?value=11/20&pvkey=&run_id=92259&search_id=93773&scans_id=92255&peptide_id=8259&charge=2&scanf=17257&mz_l=765.551&mz_h=965.551) \| [**550.335**](https://tmsf.med.harvard.edu/core/www/modules/sdig/index.php?value=11/20&pvkey=&run_id=92259&search_id=93773&scans_id=92255&peptide_id=8259&charge=2&scanf=17257&mz_l=450.335&mz_h=650.335) \| **1.365** \| **4** \| \| **V** \| **9** \| **---** \| [964.619](https://tmsf.med.harvard.edu/core/www/modules/sdig/index.php?value=11/20&pvkey=&run_id=92259&search_id=93773&scans_id=92255&peptide_id=8259&charge=2&scanf=17257&mz_l=864.619&mz_h=1064.619) \| [**437.251**](https://tmsf.med.harvard.edu/core/www/modules/sdig/index.php?value=11/20&pvkey=&run_id=92259&search_id=93773&scans_id=92255&peptide_id=8259&charge=2&scanf=17257&mz_l=337.251&mz_h=537.251) \| **-2.357** \| **3** \| \| **Y** \| **10** \| **---** \| [1127.682](https://tmsf.med.harvard.edu/core/www/modules/sdig/index.php?value=11/20&pvkey=&run_id=92259&search_id=93773&scans_id=92255&peptide_id=8259&charge=2&scanf=17257&mz_l=1027.682&mz_h=1227.682) \| [**338.182**](https://tmsf.med.harvard.edu/core/www/modules/sdig/index.php?value=11/20&pvkey=&run_id=92259&search_id=93773&scans_id=92255&peptide_id=8259&charge=2&scanf=17257&mz_l=238.182&mz_h=438.182) \| **1.716** \| **2** \| \| **R** \| **11** \| **---** \| --- \| [**175.119**](https://tmsf.med.harvard.edu/core/www/modules/sdig/index.php?value=11/20&pvkey=&run_id=92259&search_id=93773&scans_id=92255&peptide_id=8259&charge=2&scanf=17257&mz_l=75.119&mz_h=275.119) \| **-0.320** \| **1** \| |

**Sequence no 8**

| [>tr\|A0A5E4G7W8\|A0A5E4G7W8_PRUDU PREDICTED: probable aldo-keto reductase OS=Prunus dulcis OX=3755 GN=ALMOND_2B028170 PE=4 SV=1](http://www.uniprot.org/uniprot/?query=A0A5E4G7W8_PRUDU) |
| --- |
| MAGGVRRIKL GSQGLEVSAQ GLGCMGMSAF YGAPKPDADM ISLIHHAIDS GVTFLDTSDI YGPFTNEILL GKALKGGVRE KVELATKFGI SFADNKPEVR  GDPAYVRAAI EGSLKRLGVD SVDLYYQHRI DTRVPIEVTV GELKKLVEEG KVKYIGLSEA SASTIRRAHA VHPITAVQLE WSLWTRDVEE DIIPTCRELG  IGIVAYSPLG RGFFSSGAKF FENLPNDDFR KYLPRFQAEN LEHNKTIFER VSDLAARKGC TPSQLALAWV HHQGNDVCPI PGTTKIQNFN QNIGALSVKL  TPEELAELES FASADAVKGG RYQNDFSTWK NSETVRVSVI LAGF |

| Legend | | |
| --- | --- | --- |
| THISREFERE NCE | UNMATCHED | OTHERREFER ENCES |

| \| 37634.41 Da \| Protein Coverage \| \| \| --- \| --- \| --- \| \| 344 AA \| 14 AA \| **4.07 %** \| |
| --- | --- | --- | --- | --- | --- | --- |

**Data table 13**

| \| **Sequence** \| \| --- \| \| ELGIGIVAYSPLGR \| |
| --- | --- | --- |
| **Predicted Fragmentation Pattern**   \| **Seq** \| **#** \| **b: Δ Error** \| **b** \| **y** \| **y: Δ Error** \| **+1** \| \| --- \| --- \| --- \| --- \| --- \| --- \| --- \| \| **E** \| **1** \| **0.913** \| [**130.050**](https://tmsf.med.harvard.edu/core/www/modules/sdig/index.php?value=15/26&pvkey=&run_id=92258&search_id=93772&scans_id=92254&peptide_id=6631&charge=2&scanf=15679&mz_l=30.05&mz_h=230.05) \| --- \| **---** \| **14** \| \| **L** \| **2** \| **0.224** \| [**243.134**](https://tmsf.med.harvard.edu/core/www/modules/sdig/index.php?value=15/26&pvkey=&run_id=92258&search_id=93772&scans_id=92254&peptide_id=6631&charge=2&scanf=15679&mz_l=143.134&mz_h=343.134) \| [1315.773](https://tmsf.med.harvard.edu/core/www/modules/sdig/index.php?value=15/26&pvkey=&run_id=92258&search_id=93772&scans_id=92254&peptide_id=6631&charge=2&scanf=15679&mz_l=1215.773&mz_h=1415.773) \| **---** \| **13** \| \| **G** \| **3** \| **-0.411** \| [**300.155**](https://tmsf.med.harvard.edu/core/www/modules/sdig/index.php?value=15/26&pvkey=&run_id=92258&search_id=93772&scans_id=92254&peptide_id=6631&charge=2&scanf=15679&mz_l=200.155&mz_h=400.155) \| [**1202.689**](https://tmsf.med.harvard.edu/core/www/modules/sdig/index.php?value=15/26&pvkey=&run_id=92258&search_id=93772&scans_id=92254&peptide_id=6631&charge=2&scanf=15679&mz_l=1102.689&mz_h=1302.689) \| **0.122** \| **12** \| \| **I** \| **4** \| **-1.525** \| [**413.239**](https://tmsf.med.harvard.edu/core/www/modules/sdig/index.php?value=15/26&pvkey=&run_id=92258&search_id=93772&scans_id=92254&peptide_id=6631&charge=2&scanf=15679&mz_l=313.239&mz_h=513.239) \| [**1145.668**](https://tmsf.med.harvard.edu/core/www/modules/sdig/index.php?value=15/26&pvkey=&run_id=92258&search_id=93772&scans_id=92254&peptide_id=6631&charge=2&scanf=15679&mz_l=1045.668&mz_h=1245.668) \| **0.750** \| **11** \| \| **G** \| **5** \| **-0.582** \| [**470.261**](https://tmsf.med.harvard.edu/core/www/modules/sdig/index.php?value=15/26&pvkey=&run_id=92258&search_id=93772&scans_id=92254&peptide_id=6631&charge=2&scanf=15679&mz_l=370.261&mz_h=570.261) \| [**1032.584**](https://tmsf.med.harvard.edu/core/www/modules/sdig/index.php?value=15/26&pvkey=&run_id=92258&search_id=93772&scans_id=92254&peptide_id=6631&charge=2&scanf=15679&mz_l=932.584&mz_h=1132.584) \| **0.081** \| **10** \| \| **I** \| **6** \| **4.364** \| [**583.345**](https://tmsf.med.harvard.edu/core/www/modules/sdig/index.php?value=15/26&pvkey=&run_id=92258&search_id=93772&scans_id=92254&peptide_id=6631&charge=2&scanf=15679&mz_l=483.345&mz_h=683.345) \| [**975.562**](https://tmsf.med.harvard.edu/core/www/modules/sdig/index.php?value=15/26&pvkey=&run_id=92258&search_id=93772&scans_id=92254&peptide_id=6631&charge=2&scanf=15679&mz_l=875.562&mz_h=1075.562) \| **1.191** \| **9** \| \| **V** \| **7** \| **-0.374** \| [**682.413**](https://tmsf.med.harvard.edu/core/www/modules/sdig/index.php?value=15/26&pvkey=&run_id=92258&search_id=93772&scans_id=92254&peptide_id=6631&charge=2&scanf=15679&mz_l=582.413&mz_h=782.413) \| [**862.478**](https://tmsf.med.harvard.edu/core/www/modules/sdig/index.php?value=15/26&pvkey=&run_id=92258&search_id=93772&scans_id=92254&peptide_id=6631&charge=2&scanf=15679&mz_l=762.478&mz_h=962.478) \| **0.095** \| **8** \| \| **A** \| **8** \| **-8.284** \| [**753.451**](https://tmsf.med.harvard.edu/core/www/modules/sdig/index.php?value=15/26&pvkey=&run_id=92258&search_id=93772&scans_id=92254&peptide_id=6631&charge=2&scanf=15679&mz_l=653.451&mz_h=853.451) \| [**763.410**](https://tmsf.med.harvard.edu/core/www/modules/sdig/index.php?value=15/26&pvkey=&run_id=92258&search_id=93772&scans_id=92254&peptide_id=6631&charge=2&scanf=15679&mz_l=663.41&mz_h=863.41) \| **0.099** \| **7** \| \| **Y** \| **9** \| **---** \| [916.514](https://tmsf.med.harvard.edu/core/www/modules/sdig/index.php?value=15/26&pvkey=&run_id=92258&search_id=93772&scans_id=92254&peptide_id=6631&charge=2&scanf=15679&mz_l=816.514&mz_h=1016.514) \| [**692.373**](https://tmsf.med.harvard.edu/core/www/modules/sdig/index.php?value=15/26&pvkey=&run_id=92258&search_id=93772&scans_id=92254&peptide_id=6631&charge=2&scanf=15679&mz_l=592.373&mz_h=792.373) \| **1.878** \| **6** \| \| **S** \| **10** \| **---** \| [1003.546](https://tmsf.med.harvard.edu/core/www/modules/sdig/index.php?value=15/26&pvkey=&run_id=92258&search_id=93772&scans_id=92254&peptide_id=6631&charge=2&scanf=15679&mz_l=903.546&mz_h=1103.546) \| [**529.309**](https://tmsf.med.harvard.edu/core/www/modules/sdig/index.php?value=15/26&pvkey=&run_id=92258&search_id=93772&scans_id=92254&peptide_id=6631&charge=2&scanf=15679&mz_l=429.309&mz_h=629.309) \| **-0.360** \| **5** \| \| **P** \| **11** \| **---** \| [1100.599](https://tmsf.med.harvard.edu/core/www/modules/sdig/index.php?value=15/26&pvkey=&run_id=92258&search_id=93772&scans_id=92254&peptide_id=6631&charge=2&scanf=15679&mz_l=1000.599&mz_h=1200.599) \| [**442.277**](https://tmsf.med.harvard.edu/core/www/modules/sdig/index.php?value=15/26&pvkey=&run_id=92258&search_id=93772&scans_id=92254&peptide_id=6631&charge=2&scanf=15679&mz_l=342.277&mz_h=542.277) \| **-3.225** \| **4** \| \| **L** \| **12** \| **---** \| [1213.683](https://tmsf.med.harvard.edu/core/www/modules/sdig/index.php?value=15/26&pvkey=&run_id=92258&search_id=93772&scans_id=92254&peptide_id=6631&charge=2&scanf=15679&mz_l=1113.683&mz_h=1313.683) \| [**345.224**](https://tmsf.med.harvard.edu/core/www/modules/sdig/index.php?value=15/26&pvkey=&run_id=92258&search_id=93772&scans_id=92254&peptide_id=6631&charge=2&scanf=15679&mz_l=245.224&mz_h=445.224) \| **-0.687** \| **3** \| \| **G** \| **13** \| **---** \| [1270.704](https://tmsf.med.harvard.edu/core/www/modules/sdig/index.php?value=15/26&pvkey=&run_id=92258&search_id=93772&scans_id=92254&peptide_id=6631&charge=2&scanf=15679&mz_l=1170.704&mz_h=1370.704) \| [**232.140**](https://tmsf.med.harvard.edu/core/www/modules/sdig/index.php?value=15/26&pvkey=&run_id=92258&search_id=93772&scans_id=92254&peptide_id=6631&charge=2&scanf=15679&mz_l=132.14&mz_h=332.14) \| **-0.218** \| **2** \| \| **R** \| **14** \| **---** \| --- \| [**175.119**](https://tmsf.med.harvard.edu/core/www/modules/sdig/index.php?value=15/26&pvkey=&run_id=92258&search_id=93772&scans_id=92254&peptide_id=6631&charge=2&scanf=15679&mz_l=75.119&mz_h=275.119) \| **-0.059** \| **1** \| |

**Sequence no 9**

| [>tr\|A0A1U8II21\|A0A1U8II21_GOSHI UTP--glucose-1-phosphate uridylyltransferase OS=Gossypium hirsutum OX=3635 GN=LOC107895238 PE=3 SV=1](http://www.uniprot.org/uniprot/?query=A0A1U8II21_GOSHI) |
| --- |
| MEKLEHLKSA VAALSEISEN EKNGFINLVS RYLRYYYFTA ETKKLLDKLV VLKLNGGLGT TMGCTGPKSV IEVRNGLTFL DLIVIQIENL NSKYGCNVPL  VLMNSFNTHD DTLKIVDKYS NSNIEIHTFN QSQYPRLVVE DFAPLPSKGQ HGKDGWYPPG HGDVFPSLMN SGKLDAFLSQ GKEYVFVANS DNLGAIVDMK  ILNHLVQNKN EYCMEVTPKT LADVKGGTLI SYEGKVQLLE IAQVPDEHVN EFKSIEKFKI FNTNNLWVNL NAIKRLVEAD ALKMEIIPNP KEVNGIKVLQ  LETAAGAAIR FFDHAIGINV PRSRFLPVKA TSDLLLVQSD LYTLVDGFVI RNKDRANPTN PSIELGPEFK KVGNFLSRFK SIPSIIELDS LKVTGDVWFG  AGIVLKGKVS IAAKPGVKLE IPDGAVIEKK EINVPEDI |

| Legend | | |
| --- | --- | --- |
| THISREFERE NCE | UNMATCHED | OTHERREFER ENCES |

| \| 48557.66 Da \| Protein Coverage \| \| \| --- \| --- \| --- \| \| 438 AA \| 12 AA \| **2.74 %** \| |
| --- | --- | --- | --- | --- | --- | --- |

**Data table 14**

| \| **Sequence** \| \| --- \| \| FFDHAIGINVPR \| |
| --- | --- | --- |
| **Predicted Fragmentation Pattern**  **+1**   \| **Seq** \| **#** \| **b: Δ Error** \| **b** \| **y** \| **y: Δ Error** \| **+1** \| \| --- \| --- \| --- \| --- \| --- \| --- \| --- \| \| **F** \| **1** \| **---** \| [148.076](https://tmsf.med.harvard.edu/core/www/modules/sdig/index.php?value=14/44&pvkey=&run_id=92259&search_id=93773&scans_id=92255&peptide_id=3413&charge=3&scanf=12527&mz_l=48.076&mz_h=248.076) \| --- \| **---** \| **12** \| \| **F** \| **2** \| **-2.481** \| [**295.144**](https://tmsf.med.harvard.edu/core/www/modules/sdig/index.php?value=14/44&pvkey=&run_id=92259&search_id=93773&scans_id=92255&peptide_id=3413&charge=3&scanf=12527&mz_l=195.144&mz_h=395.144) \| [1238.664](https://tmsf.med.harvard.edu/core/www/modules/sdig/index.php?value=14/44&pvkey=&run_id=92259&search_id=93773&scans_id=92255&peptide_id=3413&charge=3&scanf=12527&mz_l=1138.664&mz_h=1338.664) \| **---** \| **11** \| \| **D** \| **3** \| **---** \| [410.171](https://tmsf.med.harvard.edu/core/www/modules/sdig/index.php?value=14/44&pvkey=&run_id=92259&search_id=93773&scans_id=92255&peptide_id=3413&charge=3&scanf=12527&mz_l=310.171&mz_h=510.171) \| [1091.596](https://tmsf.med.harvard.edu/core/www/modules/sdig/index.php?value=14/44&pvkey=&run_id=92259&search_id=93773&scans_id=92255&peptide_id=3413&charge=3&scanf=12527&mz_l=991.596&mz_h=1191.596) \| **---** \| **10** \| \| **H** \| **4** \| **-1.076** \| [**547.230**](https://tmsf.med.harvard.edu/core/www/modules/sdig/index.php?value=14/44&pvkey=&run_id=92259&search_id=93773&scans_id=92255&peptide_id=3413&charge=3&scanf=12527&mz_l=447.23&mz_h=647.23) \| [976.569](https://tmsf.med.harvard.edu/core/www/modules/sdig/index.php?value=14/44&pvkey=&run_id=92259&search_id=93773&scans_id=92255&peptide_id=3413&charge=3&scanf=12527&mz_l=876.569&mz_h=1076.569) \| **---** \| **9** \| \| **A** \| **5** \| **-1.354** \| [**618.267**](https://tmsf.med.harvard.edu/core/www/modules/sdig/index.php?value=14/44&pvkey=&run_id=92259&search_id=93773&scans_id=92255&peptide_id=3413&charge=3&scanf=12527&mz_l=518.267&mz_h=718.267) \| [**839.510**](https://tmsf.med.harvard.edu/core/www/modules/sdig/index.php?value=14/44&pvkey=&run_id=92259&search_id=93773&scans_id=92255&peptide_id=3413&charge=3&scanf=12527&mz_l=739.51&mz_h=939.51) \| **-0.433** \| **8** \| \| **I** \| **6** \| **-3.423** \| [**731.351**](https://tmsf.med.harvard.edu/core/www/modules/sdig/index.php?value=14/44&pvkey=&run_id=92259&search_id=93773&scans_id=92255&peptide_id=3413&charge=3&scanf=12527&mz_l=631.351&mz_h=831.351) \| [**768.473**](https://tmsf.med.harvard.edu/core/www/modules/sdig/index.php?value=14/44&pvkey=&run_id=92259&search_id=93773&scans_id=92255&peptide_id=3413&charge=3&scanf=12527&mz_l=668.473&mz_h=868.473) \| **-0.071** \| **7** \| \| **G** \| **7** \| **-1.214** \| [**788.373**](https://tmsf.med.harvard.edu/core/www/modules/sdig/index.php?value=14/44&pvkey=&run_id=92259&search_id=93773&scans_id=92255&peptide_id=3413&charge=3&scanf=12527&mz_l=688.373&mz_h=888.373) \| [**655.389**](https://tmsf.med.harvard.edu/core/www/modules/sdig/index.php?value=14/44&pvkey=&run_id=92259&search_id=93773&scans_id=92255&peptide_id=3413&charge=3&scanf=12527&mz_l=555.389&mz_h=755.389) \| **-0.334** \| **6** \| \| **I** \| **8** \| **-0.405** \| [**901.457**](https://tmsf.med.harvard.edu/core/www/modules/sdig/index.php?value=14/44&pvkey=&run_id=92259&search_id=93773&scans_id=92255&peptide_id=3413&charge=3&scanf=12527&mz_l=801.457&mz_h=1001.457) \| [**598.367**](https://tmsf.med.harvard.edu/core/www/modules/sdig/index.php?value=14/44&pvkey=&run_id=92259&search_id=93773&scans_id=92255&peptide_id=3413&charge=3&scanf=12527&mz_l=498.367&mz_h=698.367) \| **-2.134** \| **5** \| \| **N** \| **9** \| **1.664** \| [**1015.500**](https://tmsf.med.harvard.edu/core/www/modules/sdig/index.php?value=14/44&pvkey=&run_id=92259&search_id=93773&scans_id=92255&peptide_id=3413&charge=3&scanf=12527&mz_l=915.5&mz_h=1115.5) \| [**485.283**](https://tmsf.med.harvard.edu/core/www/modules/sdig/index.php?value=14/44&pvkey=&run_id=92259&search_id=93773&scans_id=92255&peptide_id=3413&charge=3&scanf=12527&mz_l=385.283&mz_h=585.283) \| **-0.392** \| **4** \| \| **V** \| **10** \| **---** \| [1114.568](https://tmsf.med.harvard.edu/core/www/modules/sdig/index.php?value=14/44&pvkey=&run_id=92259&search_id=93773&scans_id=92255&peptide_id=3413&charge=3&scanf=12527&mz_l=1014.568&mz_h=1214.568) \| [**371.240**](https://tmsf.med.harvard.edu/core/www/modules/sdig/index.php?value=14/44&pvkey=&run_id=92259&search_id=93773&scans_id=92255&peptide_id=3413&charge=3&scanf=12527&mz_l=271.24&mz_h=471.24) \| **0.280** \| **3** \| \| **P** \| **11** \| **---** \| [1211.621](https://tmsf.med.harvard.edu/core/www/modules/sdig/index.php?value=14/44&pvkey=&run_id=92259&search_id=93773&scans_id=92255&peptide_id=3413&charge=3&scanf=12527&mz_l=1111.621&mz_h=1311.621) \| [**272.172**](https://tmsf.med.harvard.edu/core/www/modules/sdig/index.php?value=14/44&pvkey=&run_id=92259&search_id=93773&scans_id=92255&peptide_id=3413&charge=3&scanf=12527&mz_l=172.172&mz_h=372.172) \| **-0.314** \| **2** \| \| **R** \| **12** \| **---** \| --- \| [**175.119**](https://tmsf.med.harvard.edu/core/www/modules/sdig/index.php?value=14/44&pvkey=&run_id=92259&search_id=93773&scans_id=92255&peptide_id=3413&charge=3&scanf=12527&mz_l=75.119&mz_h=275.119) \| **0.029** \| **1** \|   **+2**   \| **Seq** \| **#** \| **b: Δ Error** \| **b** \| **y** \| **y: Δ Error** \| **+1** \| \| --- \| --- \| --- \| --- \| --- \| --- \| --- \| \| **F** \| **1** \| **---** \| [74.541](https://tmsf.med.harvard.edu/core/www/modules/sdig/index.php?value=14/44&pvkey=&run_id=92259&search_id=93773&scans_id=92255&peptide_id=3413&charge=3&scanf=12527&mz_l=-25.459&mz_h=174.541) \| --- \| **---** \| **12** \| \| **F** \| **2** \| **---** \| [148.076](https://tmsf.med.harvard.edu/core/www/modules/sdig/index.php?value=14/44&pvkey=&run_id=92259&search_id=93773&scans_id=92255&peptide_id=3413&charge=3&scanf=12527&mz_l=48.076&mz_h=248.076) \| [**619.836**](https://tmsf.med.harvard.edu/core/www/modules/sdig/index.php?value=14/44&pvkey=&run_id=92259&search_id=93773&scans_id=92255&peptide_id=3413&charge=3&scanf=12527&mz_l=519.836&mz_h=719.836) \| **0.556** \| **11** \| \| **D** \| **3** \| **---** \| [205.589](https://tmsf.med.harvard.edu/core/www/modules/sdig/index.php?value=14/44&pvkey=&run_id=92259&search_id=93773&scans_id=92255&peptide_id=3413&charge=3&scanf=12527&mz_l=105.589&mz_h=305.589) \| [**546.301**](https://tmsf.med.harvard.edu/core/www/modules/sdig/index.php?value=14/44&pvkey=&run_id=92259&search_id=93773&scans_id=92255&peptide_id=3413&charge=3&scanf=12527&mz_l=446.301&mz_h=646.301) \| **-2.336** \| **10** \| \| **H** \| **4** \| **0.016** \| [**274.119**](https://tmsf.med.harvard.edu/core/www/modules/sdig/index.php?value=14/44&pvkey=&run_id=92259&search_id=93773&scans_id=92255&peptide_id=3413&charge=3&scanf=12527&mz_l=174.119&mz_h=374.119) \| [**488.788**](https://tmsf.med.harvard.edu/core/www/modules/sdig/index.php?value=14/44&pvkey=&run_id=92259&search_id=93773&scans_id=92255&peptide_id=3413&charge=3&scanf=12527&mz_l=388.788&mz_h=588.788) \| **-1.335** \| **9** \| \| **A** \| **5** \| **-0.880** \| [**309.637**](https://tmsf.med.harvard.edu/core/www/modules/sdig/index.php?value=14/44&pvkey=&run_id=92259&search_id=93773&scans_id=92255&peptide_id=3413&charge=3&scanf=12527&mz_l=209.637&mz_h=409.637) \| [420.259](https://tmsf.med.harvard.edu/core/www/modules/sdig/index.php?value=14/44&pvkey=&run_id=92259&search_id=93773&scans_id=92255&peptide_id=3413&charge=3&scanf=12527&mz_l=320.259&mz_h=520.259) \| **---** \| **8** \| \| **I** \| **6** \| **---** \| [366.179](https://tmsf.med.harvard.edu/core/www/modules/sdig/index.php?value=14/44&pvkey=&run_id=92259&search_id=93773&scans_id=92255&peptide_id=3413&charge=3&scanf=12527&mz_l=266.179&mz_h=466.179) \| [384.740](https://tmsf.med.harvard.edu/core/www/modules/sdig/index.php?value=14/44&pvkey=&run_id=92259&search_id=93773&scans_id=92255&peptide_id=3413&charge=3&scanf=12527&mz_l=284.74&mz_h=484.74) \| **---** \| **7** \| \| **G** \| **7** \| **---** \| [394.690](https://tmsf.med.harvard.edu/core/www/modules/sdig/index.php?value=14/44&pvkey=&run_id=92259&search_id=93773&scans_id=92255&peptide_id=3413&charge=3&scanf=12527&mz_l=294.69&mz_h=494.69) \| [328.198](https://tmsf.med.harvard.edu/core/www/modules/sdig/index.php?value=14/44&pvkey=&run_id=92259&search_id=93773&scans_id=92255&peptide_id=3413&charge=3&scanf=12527&mz_l=228.198&mz_h=428.198) \| **---** \| **6** \| \| **I** \| **8** \| **-5.830** \| [**451.232**](https://tmsf.med.harvard.edu/core/www/modules/sdig/index.php?value=14/44&pvkey=&run_id=92259&search_id=93773&scans_id=92255&peptide_id=3413&charge=3&scanf=12527&mz_l=351.232&mz_h=551.232) \| [299.687](https://tmsf.med.harvard.edu/core/www/modules/sdig/index.php?value=14/44&pvkey=&run_id=92259&search_id=93773&scans_id=92255&peptide_id=3413&charge=3&scanf=12527&mz_l=199.687&mz_h=399.687) \| **---** \| **5** \| \| **N** \| **9** \| **---** \| [508.253](https://tmsf.med.harvard.edu/core/www/modules/sdig/index.php?value=14/44&pvkey=&run_id=92259&search_id=93773&scans_id=92255&peptide_id=3413&charge=3&scanf=12527&mz_l=408.253&mz_h=608.253) \| [**243.145**](https://tmsf.med.harvard.edu/core/www/modules/sdig/index.php?value=14/44&pvkey=&run_id=92259&search_id=93773&scans_id=92255&peptide_id=3413&charge=3&scanf=12527&mz_l=143.145&mz_h=343.145) \| **-48.428** \| **4** \| \| **V** \| **10** \| **---** \| [557.788](https://tmsf.med.harvard.edu/core/www/modules/sdig/index.php?value=14/44&pvkey=&run_id=92259&search_id=93773&scans_id=92255&peptide_id=3413&charge=3&scanf=12527&mz_l=457.788&mz_h=657.788) \| [**186.124**](https://tmsf.med.harvard.edu/core/www/modules/sdig/index.php?value=14/44&pvkey=&run_id=92259&search_id=93773&scans_id=92255&peptide_id=3413&charge=3&scanf=12527&mz_l=86.124&mz_h=286.124) \| **-0.247** \| **3** \| \| **P** \| **11** \| **---** \| [606.314](https://tmsf.med.harvard.edu/core/www/modules/sdig/index.php?value=14/44&pvkey=&run_id=92259&search_id=93773&scans_id=92255&peptide_id=3413&charge=3&scanf=12527&mz_l=506.314&mz_h=706.314) \| [**136.589**](https://tmsf.med.harvard.edu/core/www/modules/sdig/index.php?value=14/44&pvkey=&run_id=92259&search_id=93773&scans_id=92255&peptide_id=3413&charge=3&scanf=12527&mz_l=36.589&mz_h=236.589) \| **-0.583** \| **2** \| \| **R** \| **12** \| **---** \| --- \| [88.063](https://tmsf.med.harvard.edu/core/www/modules/sdig/index.php?value=14/44&pvkey=&run_id=92259&search_id=93773&scans_id=92255&peptide_id=3413&charge=3&scanf=12527&mz_l=-11.937&mz_h=188.063) \| **---** \| **1** \| |

**Sequence no 10**

| [>tr\|A0A5E4EZP4\|A0A5E4EZP4_PRUDU PREDICTED: vicilin OS=Prunus dulcis OX=3755 GN=ALMOND_2B021144 PE=4 SV=1](http://www.uniprot.org/uniprot/?query=A0A5E4EZP4_PRUDU) |
| --- |
| MVIHIKPRLP LFLFLSLLFL AFSVSFTLGQ DREQHCQKRC QDFKRPIIHS ECIYQCRMWG PWSLPPAESS NHHKQQQQHE QEQQCKQRCQ PQHGHRQQQQ  CQQECYEQIR QQREREEMQQ MCQQSCEMQG SGGQQQQRQQ CQRQCEQQVE QLKQCQLRCQ MQGQGQQQEQ CQRTCRQELE DQQRRQQQEQ GPGGGDNKLE  DETLNGEPRR RFEQCMQGCE RQQQGQGEQE QCPRQCREQF DKEKRQYQQC KQSCEKSSQH DDEKRQCKQQ CKQQISLQQG GGNQEEDDDA LNGSGYGGAK  ERFEQCKLSC QSQQGQGQQE QCPKQCKQQF EHEKHQYKQC KRGCENQARD DVQKEQCKQQ CTQQMSQQYG QQQEGSTGGG GLNQQQEEEG QMGSQGQSQS  QSQSNNPFYF PSHRFQPRFQ SNEGGLYVLE RFTQRQSEVL RGIKNYRLAI FEARPNTFVL PHHCDAEAIY VVLSGQCTCT LLMQDRKESF NMEHGDVIRV  PAGATTYLVN NNSDKTLRIA KLLQPVNNPG RFEEFFPAGS RNSESYFSVF SNDILESAFN TPREQLEHGF KQGQQQQGQG IVMRAPREQL QALSQAASSR  RRGDRQSQGP FNLRQQRPVH SNNYGQFFEA RPEEFNQLQD MDASVSCIEI NQQAMMVPHF NSKATHLIMV VEGKGLVEMA CPYLANQSQE IMGQQEQQGE  QSGRYMKVTA QLSPGDVFVI PAGHPVALVA QNQNLRILGF GLYAQNNKRN FLAGQEDNII MHMDREARQL AFGPEMEQIF SKQQQSYFVP TQQGRSRNQH  LSSILEFAGV I |

| Legend | | |
| --- | --- | --- |
| THISREFERE NCE | UNMATCHED | OTHERREFER ENCES |

| \| 93710.38 Da \| Protein Coverage \| \| \| --- \| --- \| --- \| \| 811 AA \| 183 AA \| **22.56 %** \| |
| --- | --- | --- | --- | --- | --- | --- |

**Data Table 15**

| \| **Sequence** \| \| --- \| \| NSESYFSVFSNDILESAFNTPR \| |
| --- | --- | --- |
| **Predicted Fragmentation Pattern**  **+1**   \| **Seq** \| **#** \| **b: Δ Error** \| **b** \| **y** \| **y: Δ Error** \| **+1** \| \| --- \| --- \| --- \| --- \| --- \| --- \| --- \| \| **N** \| **1** \| **83.531** \| [**115.050**](https://tmsf.med.harvard.edu/core/www/modules/sdig/index.php?value=23/84&pvkey=&run_id=92258&search_id=93772&scans_id=92254&peptide_id=8658&charge=3&scanf=17642&mz_l=15.05&mz_h=215.05) \| --- \| **---** \| **22** \| \| **S** \| **2** \| **-0.917** \| [**202.082**](https://tmsf.med.harvard.edu/core/www/modules/sdig/index.php?value=23/84&pvkey=&run_id=92258&search_id=93772&scans_id=92254&peptide_id=8658&charge=3&scanf=17642&mz_l=102.082&mz_h=302.082) \| [2410.120](https://tmsf.med.harvard.edu/core/www/modules/sdig/index.php?value=23/84&pvkey=&run_id=92258&search_id=93772&scans_id=92254&peptide_id=8658&charge=3&scanf=17642&mz_l=2310.12&mz_h=2510.12) \| **---** \| **21** \| \| **E** \| **3** \| **-4.356** \| [**331.125**](https://tmsf.med.harvard.edu/core/www/modules/sdig/index.php?value=23/84&pvkey=&run_id=92258&search_id=93772&scans_id=92254&peptide_id=8658&charge=3&scanf=17642&mz_l=231.125&mz_h=431.125) \| [2323.088](https://tmsf.med.harvard.edu/core/www/modules/sdig/index.php?value=23/84&pvkey=&run_id=92258&search_id=93772&scans_id=92254&peptide_id=8658&charge=3&scanf=17642&mz_l=2223.088&mz_h=2423.088) \| **---** \| **20** \| \| **S** \| **4** \| **-2.319** \| [**418.157**](https://tmsf.med.harvard.edu/core/www/modules/sdig/index.php?value=23/84&pvkey=&run_id=92258&search_id=93772&scans_id=92254&peptide_id=8658&charge=3&scanf=17642&mz_l=318.157&mz_h=518.157) \| [2194.045](https://tmsf.med.harvard.edu/core/www/modules/sdig/index.php?value=23/84&pvkey=&run_id=92258&search_id=93772&scans_id=92254&peptide_id=8658&charge=3&scanf=17642&mz_l=2094.045&mz_h=2294.045) \| **---** \| **19** \| \| **Y** \| **5** \| **-3.199** \| [**581.220**](https://tmsf.med.harvard.edu/core/www/modules/sdig/index.php?value=23/84&pvkey=&run_id=92258&search_id=93772&scans_id=92254&peptide_id=8658&charge=3&scanf=17642&mz_l=481.22&mz_h=681.22) \| [2107.013](https://tmsf.med.harvard.edu/core/www/modules/sdig/index.php?value=23/84&pvkey=&run_id=92258&search_id=93772&scans_id=92254&peptide_id=8658&charge=3&scanf=17642&mz_l=2007.013&mz_h=2207.013) \| **---** \| **18** \| \| **F** \| **6** \| **-6.231** \| [**728.289**](https://tmsf.med.harvard.edu/core/www/modules/sdig/index.php?value=23/84&pvkey=&run_id=92258&search_id=93772&scans_id=92254&peptide_id=8658&charge=3&scanf=17642&mz_l=628.289&mz_h=828.289) \| [1943.950](https://tmsf.med.harvard.edu/core/www/modules/sdig/index.php?value=23/84&pvkey=&run_id=92258&search_id=93772&scans_id=92254&peptide_id=8658&charge=3&scanf=17642&mz_l=1843.95&mz_h=2043.95) \| **---** \| **17** \| \| **S** \| **7** \| **-1.730** \| [**815.321**](https://tmsf.med.harvard.edu/core/www/modules/sdig/index.php?value=23/84&pvkey=&run_id=92258&search_id=93772&scans_id=92254&peptide_id=8658&charge=3&scanf=17642&mz_l=715.321&mz_h=915.321) \| [**1796.881**](https://tmsf.med.harvard.edu/core/www/modules/sdig/index.php?value=23/84&pvkey=&run_id=92258&search_id=93772&scans_id=92254&peptide_id=8658&charge=3&scanf=17642&mz_l=1696.881&mz_h=1896.881) \| **-5.710** \| **16** \| \| **V** \| **8** \| **-1.402** \| [**914.389**](https://tmsf.med.harvard.edu/core/www/modules/sdig/index.php?value=23/84&pvkey=&run_id=92258&search_id=93772&scans_id=92254&peptide_id=8658&charge=3&scanf=17642&mz_l=814.389&mz_h=1014.389) \| [**1709.849**](https://tmsf.med.harvard.edu/core/www/modules/sdig/index.php?value=23/84&pvkey=&run_id=92258&search_id=93772&scans_id=92254&peptide_id=8658&charge=3&scanf=17642&mz_l=1609.849&mz_h=1809.849) \| **-4.189** \| **15** \| \| **F** \| **9** \| **-0.626** \| [**1061.457**](https://tmsf.med.harvard.edu/core/www/modules/sdig/index.php?value=23/84&pvkey=&run_id=92258&search_id=93772&scans_id=92254&peptide_id=8658&charge=3&scanf=17642&mz_l=961.457&mz_h=1161.457) \| [**1610.781**](https://tmsf.med.harvard.edu/core/www/modules/sdig/index.php?value=23/84&pvkey=&run_id=92258&search_id=93772&scans_id=92254&peptide_id=8658&charge=3&scanf=17642&mz_l=1510.781&mz_h=1710.781) \| **-1.458** \| **14** \| \| **S** \| **10** \| **-1.363** \| [**1148.489**](https://tmsf.med.harvard.edu/core/www/modules/sdig/index.php?value=23/84&pvkey=&run_id=92258&search_id=93772&scans_id=92254&peptide_id=8658&charge=3&scanf=17642&mz_l=1048.489&mz_h=1248.489) \| [**1463.712**](https://tmsf.med.harvard.edu/core/www/modules/sdig/index.php?value=23/84&pvkey=&run_id=92258&search_id=93772&scans_id=92254&peptide_id=8658&charge=3&scanf=17642&mz_l=1363.712&mz_h=1563.712) \| **-1.734** \| **13** \| \| **N** \| **11** \| **---** \| [1262.532](https://tmsf.med.harvard.edu/core/www/modules/sdig/index.php?value=23/84&pvkey=&run_id=92258&search_id=93772&scans_id=92254&peptide_id=8658&charge=3&scanf=17642&mz_l=1162.532&mz_h=1362.532) \| [**1376.680**](https://tmsf.med.harvard.edu/core/www/modules/sdig/index.php?value=23/84&pvkey=&run_id=92258&search_id=93772&scans_id=92254&peptide_id=8658&charge=3&scanf=17642&mz_l=1276.68&mz_h=1476.68) \| **1.471** \| **12** \| \| **D** \| **12** \| **---** \| [1377.559](https://tmsf.med.harvard.edu/core/www/modules/sdig/index.php?value=23/84&pvkey=&run_id=92258&search_id=93772&scans_id=92254&peptide_id=8658&charge=3&scanf=17642&mz_l=1277.559&mz_h=1477.559) \| [**1262.638**](https://tmsf.med.harvard.edu/core/www/modules/sdig/index.php?value=23/84&pvkey=&run_id=92258&search_id=93772&scans_id=92254&peptide_id=8658&charge=3&scanf=17642&mz_l=1162.638&mz_h=1362.638) \| **3.408** \| **11** \| \| **I** \| **13** \| **0.176** \| [**1490.643**](https://tmsf.med.harvard.edu/core/www/modules/sdig/index.php?value=23/84&pvkey=&run_id=92258&search_id=93772&scans_id=92254&peptide_id=8658&charge=3&scanf=17642&mz_l=1390.643&mz_h=1590.643) \| [**1147.611**](https://tmsf.med.harvard.edu/core/www/modules/sdig/index.php?value=23/84&pvkey=&run_id=92258&search_id=93772&scans_id=92254&peptide_id=8658&charge=3&scanf=17642&mz_l=1047.611&mz_h=1247.611) \| **-0.641** \| **10** \| \| **L** \| **14** \| **---** \| [1603.727](https://tmsf.med.harvard.edu/core/www/modules/sdig/index.php?value=23/84&pvkey=&run_id=92258&search_id=93772&scans_id=92254&peptide_id=8658&charge=3&scanf=17642&mz_l=1503.727&mz_h=1703.727) \| [**1034.527**](https://tmsf.med.harvard.edu/core/www/modules/sdig/index.php?value=23/84&pvkey=&run_id=92258&search_id=93772&scans_id=92254&peptide_id=8658&charge=3&scanf=17642&mz_l=934.527&mz_h=1134.527) \| **-1.107** \| **9** \| \| **E** \| **15** \| **---** \| [1732.770](https://tmsf.med.harvard.edu/core/www/modules/sdig/index.php?value=23/84&pvkey=&run_id=92258&search_id=93772&scans_id=92254&peptide_id=8658&charge=3&scanf=17642&mz_l=1632.77&mz_h=1832.77) \| [**921.442**](https://tmsf.med.harvard.edu/core/www/modules/sdig/index.php?value=23/84&pvkey=&run_id=92258&search_id=93772&scans_id=92254&peptide_id=8658&charge=3&scanf=17642&mz_l=821.442&mz_h=1021.442) \| **-1.222** \| **8** \| \| **S** \| **16** \| **---** \| [1819.802](https://tmsf.med.harvard.edu/core/www/modules/sdig/index.php?value=23/84&pvkey=&run_id=92258&search_id=93772&scans_id=92254&peptide_id=8658&charge=3&scanf=17642&mz_l=1719.802&mz_h=1919.802) \| [**792.400**](https://tmsf.med.harvard.edu/core/www/modules/sdig/index.php?value=23/84&pvkey=&run_id=92258&search_id=93772&scans_id=92254&peptide_id=8658&charge=3&scanf=17642&mz_l=692.4&mz_h=892.4) \| **-1.356** \| **7** \| \| **A** \| **17** \| **---** \| [1890.839](https://tmsf.med.harvard.edu/core/www/modules/sdig/index.php?value=23/84&pvkey=&run_id=92258&search_id=93772&scans_id=92254&peptide_id=8658&charge=3&scanf=17642&mz_l=1790.839&mz_h=1990.839) \| [**705.368**](https://tmsf.med.harvard.edu/core/www/modules/sdig/index.php?value=23/84&pvkey=&run_id=92258&search_id=93772&scans_id=92254&peptide_id=8658&charge=3&scanf=17642&mz_l=605.368&mz_h=805.368) \| **-0.766** \| **6** \| \| **F** \| **18** \| **---** \| [2037.908](https://tmsf.med.harvard.edu/core/www/modules/sdig/index.php?value=23/84&pvkey=&run_id=92258&search_id=93772&scans_id=92254&peptide_id=8658&charge=3&scanf=17642&mz_l=1937.908&mz_h=2137.908) \| [**634.331**](https://tmsf.med.harvard.edu/core/www/modules/sdig/index.php?value=23/84&pvkey=&run_id=92258&search_id=93772&scans_id=92254&peptide_id=8658&charge=3&scanf=17642&mz_l=534.331&mz_h=734.331) \| **-0.941** \| **5** \| \| **N** \| **19** \| **---** \| [2151.951](https://tmsf.med.harvard.edu/core/www/modules/sdig/index.php?value=23/84&pvkey=&run_id=92258&search_id=93772&scans_id=92254&peptide_id=8658&charge=3&scanf=17642&mz_l=2051.951&mz_h=2251.951) \| [**487.262**](https://tmsf.med.harvard.edu/core/www/modules/sdig/index.php?value=23/84&pvkey=&run_id=92258&search_id=93772&scans_id=92254&peptide_id=8658&charge=3&scanf=17642&mz_l=387.262&mz_h=587.262) \| **-1.427** \| **4** \| \| **T** \| **20** \| **---** \| [2252.998](https://tmsf.med.harvard.edu/core/www/modules/sdig/index.php?value=23/84&pvkey=&run_id=92258&search_id=93772&scans_id=92254&peptide_id=8658&charge=3&scanf=17642&mz_l=2152.998&mz_h=2352.998) \| [**373.219**](https://tmsf.med.harvard.edu/core/www/modules/sdig/index.php?value=23/84&pvkey=&run_id=92258&search_id=93772&scans_id=92254&peptide_id=8658&charge=3&scanf=17642&mz_l=273.219&mz_h=473.219) \| **-1.074** \| **3** \| \| **P** \| **21** \| **---** \| [2350.051](https://tmsf.med.harvard.edu/core/www/modules/sdig/index.php?value=23/84&pvkey=&run_id=92258&search_id=93772&scans_id=92254&peptide_id=8658&charge=3&scanf=17642&mz_l=2250.051&mz_h=2450.051) \| [**272.172**](https://tmsf.med.harvard.edu/core/www/modules/sdig/index.php?value=23/84&pvkey=&run_id=92258&search_id=93772&scans_id=92254&peptide_id=8658&charge=3&scanf=17642&mz_l=172.172&mz_h=372.172) \| **-0.987** \| **2** \| \| **R** \| **22** \| **---** \| --- \| [**175.119**](https://tmsf.med.harvard.edu/core/www/modules/sdig/index.php?value=23/84&pvkey=&run_id=92258&search_id=93772&scans_id=92254&peptide_id=8658&charge=3&scanf=17642&mz_l=75.119&mz_h=275.119) \| **-0.930** \| **1** \| |

**Sequence no 11**

| [>tr\|A0A1U8IN61\|A0A1U8IN61_GOSHI Probable serine protease EDA2 OS=Gossypium hirsutum OX=3635 GN=LOC107896563 PE=3 SV=1](http://www.uniprot.org/uniprot/?query=A0A1U8IN61_GOSHI) |
| --- |
| MGMMRLSKCL VSLLFLVALS GFTHGFVMSP HTLLNRLSQN SNYYLTTEEH WFDQTLDHYS PYDHRQFKQR YYEFLDNFQV PDGPIFLKIC GESSCSGISN  DYLGVLAKKF GAAVVSLEHR YYGKSSPFKS HTTENLKYLS SKQALFDLAV FRQWYQESLN LKRNKTGAEN SWFVFGISYS GALSAWFRLK FPHLTCGSLA  SSGVVLAVYN YTDFDKQVGE SAGPECKAVL QEISELVDRS LESNRKELKK QFGAAELEID GDFLYFLADA AVIAFQYGNP DALCTPLVEA KKAGEDLVAA  YAKYVKDFYV GTFGVSVETY NQNHLKNTAV NEGNSDRLWW FQVCTEVAYF QVAPSNDSIR STKINTKYHL DLCKNVFGEG IYPEVDMTNI YYGGTKIAGS  KIVFTNGSQD PWRHASKQTS SPDMPSYIIT CHNCGHGTDM RGCPQSPLSI EGNAENCSAP DAVNKVRQKM IEHIDLWLSE CKGTGRSSL |

| Legend | | |
| --- | --- | --- |
| THISREFERE NCE | UNMATCHED | OTHERREFER ENCES |

| \| 54863.93 Da \| Protein Coverage \| \| \| --- \| --- \| --- \| \| 489 AA \| 10 AA \| **2.04 %** \| |
| --- | --- | --- | --- | --- | --- | --- |

**Table no 16**

| \| **Sequence** \| \| --- \| \| QALFDLAVFR \| |
| --- | --- | --- |
| **Predicted Fragmentation Pattern**   \| **Seq** \| **#** \| **b: Δ Error** \| **b** \| **y** \| **y: Δ Error** \| **+1** \| \| --- \| --- \| --- \| --- \| --- \| --- \| --- \| \| **Q** \| **1** \| **0.618** \| [**129.066**](https://tmsf.med.harvard.edu/core/www/modules/sdig/index.php?run_id=92259&search_id=93773&scans_id=92255&peptide_id=7420&scanf=16441&charge=2&value=K.QALFDLAVFR.Q&mz_l=29.066&mz_h=229.066) \| --- \| **---** \| **10** \| \| **A** \| **2** \| **0.148** \| [**200.103**](https://tmsf.med.harvard.edu/core/www/modules/sdig/index.php?run_id=92259&search_id=93773&scans_id=92255&peptide_id=7420&scanf=16441&charge=2&value=K.QALFDLAVFR.Q&mz_l=100.103&mz_h=300.103) \| [**1051.593**](https://tmsf.med.harvard.edu/core/www/modules/sdig/index.php?run_id=92259&search_id=93773&scans_id=92255&peptide_id=7420&scanf=16441&charge=2&value=K.QALFDLAVFR.Q&mz_l=951.593&mz_h=1151.593) \| **-1.265** \| **9** \| \| **L** \| **3** \| **-0.842** \| [**313.187**](https://tmsf.med.harvard.edu/core/www/modules/sdig/index.php?run_id=92259&search_id=93773&scans_id=92255&peptide_id=7420&scanf=16441&charge=2&value=K.QALFDLAVFR.Q&mz_l=213.187&mz_h=413.187) \| [**980.556**](https://tmsf.med.harvard.edu/core/www/modules/sdig/index.php?run_id=92259&search_id=93773&scans_id=92255&peptide_id=7420&scanf=16441&charge=2&value=K.QALFDLAVFR.Q&mz_l=880.556&mz_h=1080.556) \| **-0.605** \| **8** \| \| **F** \| **4** \| **---** \| [460.255](https://tmsf.med.harvard.edu/core/www/modules/sdig/index.php?run_id=92259&search_id=93773&scans_id=92255&peptide_id=7420&scanf=16441&charge=2&value=K.QALFDLAVFR.Q&mz_l=360.255&mz_h=560.255) \| [**867.472**](https://tmsf.med.harvard.edu/core/www/modules/sdig/index.php?run_id=92259&search_id=93773&scans_id=92255&peptide_id=7420&scanf=16441&charge=2&value=K.QALFDLAVFR.Q&mz_l=767.472&mz_h=967.472) \| **-0.451** \| **7** \| \| **D** \| **5** \| **---** \| [575.282](https://tmsf.med.harvard.edu/core/www/modules/sdig/index.php?run_id=92259&search_id=93773&scans_id=92255&peptide_id=7420&scanf=16441&charge=2&value=K.QALFDLAVFR.Q&mz_l=475.282&mz_h=675.282) \| [**720.404**](https://tmsf.med.harvard.edu/core/www/modules/sdig/index.php?run_id=92259&search_id=93773&scans_id=92255&peptide_id=7420&scanf=16441&charge=2&value=K.QALFDLAVFR.Q&mz_l=620.404&mz_h=820.404) \| **-0.383** \| **6** \| \| **L** \| **6** \| **---** \| [688.366](https://tmsf.med.harvard.edu/core/www/modules/sdig/index.php?run_id=92259&search_id=93773&scans_id=92255&peptide_id=7420&scanf=16441&charge=2&value=K.QALFDLAVFR.Q&mz_l=588.366&mz_h=788.366) \| [**605.377**](https://tmsf.med.harvard.edu/core/www/modules/sdig/index.php?run_id=92259&search_id=93773&scans_id=92255&peptide_id=7420&scanf=16441&charge=2&value=K.QALFDLAVFR.Q&mz_l=505.377&mz_h=705.377) \| **-0.512** \| **5** \| \| **A** \| **7** \| **---** \| [759.404](https://tmsf.med.harvard.edu/core/www/modules/sdig/index.php?run_id=92259&search_id=93773&scans_id=92255&peptide_id=7420&scanf=16441&charge=2&value=K.QALFDLAVFR.Q&mz_l=659.404&mz_h=859.404) \| [**492.293**](https://tmsf.med.harvard.edu/core/www/modules/sdig/index.php?run_id=92259&search_id=93773&scans_id=92255&peptide_id=7420&scanf=16441&charge=2&value=K.QALFDLAVFR.Q&mz_l=392.293&mz_h=592.293) \| **-0.716** \| **4** \| \| **V** \| **8** \| **---** \| [858.472](https://tmsf.med.harvard.edu/core/www/modules/sdig/index.php?run_id=92259&search_id=93773&scans_id=92255&peptide_id=7420&scanf=16441&charge=2&value=K.QALFDLAVFR.Q&mz_l=758.472&mz_h=958.472) \| [**421.256**](https://tmsf.med.harvard.edu/core/www/modules/sdig/index.php?run_id=92259&search_id=93773&scans_id=92255&peptide_id=7420&scanf=16441&charge=2&value=K.QALFDLAVFR.Q&mz_l=321.256&mz_h=521.256) \| **-0.537** \| **3** \| \| **F** \| **9** \| **---** \| [1005.540](https://tmsf.med.harvard.edu/core/www/modules/sdig/index.php?run_id=92259&search_id=93773&scans_id=92255&peptide_id=7420&scanf=16441&charge=2&value=K.QALFDLAVFR.Q&mz_l=905.54&mz_h=1105.54) \| [**322.187**](https://tmsf.med.harvard.edu/core/www/modules/sdig/index.php?run_id=92259&search_id=93773&scans_id=92255&peptide_id=7420&scanf=16441&charge=2&value=K.QALFDLAVFR.Q&mz_l=222.187&mz_h=422.187) \| **0.130** \| **2** \| \| **R** \| **10** \| **---** \| --- \| [**175.119**](https://tmsf.med.harvard.edu/core/www/modules/sdig/index.php?run_id=92259&search_id=93773&scans_id=92255&peptide_id=7420&scanf=16441&charge=2&value=K.QALFDLAVFR.Q&mz_l=75.119&mz_h=275.119) \| **0.290** \| **1** \| |

**Sequence no 12**

| [>tr\|A0A1U8IIY2\|A0A1U8IIY2_GOSHI Amine oxidase OS=Gossypium hirsutum OX=3635 GN=LOC107895118 PE=3 SV=1](http://www.uniprot.org/uniprot/?query=A0A1U8IIY2_GOSHI) |
| --- |
| MESKPFFRFL FFFIAIAFLF LFAWTHLHYA PSPGVTTLLD CASNSPWCTS SKNRFQSKQP TLTKTPRAST SQRRHHESAV PRHPLDPLTI QEFNKVRKIL  SSHALFKSSN NYALHSVVLE EPNKELVLKW KKGQPLFPRI ASVIARANGV SHVLTVNLET DNVTVLNTAP PTGYPTMTIE DMTSATWAPL SNAKFNRTII  ERGVNLKDVA CLPISLGWFG KKEENRRLIK VQCYSMEDTA NFYMRPIEGL TVLLDMDTKE VVEVSDTGRS IPIPKATNTD YRFSEQKLQQ DLNSINPISI  EQPKGPSFVI EDEHLVKWAN WEFHLKPDPR AGVVVSRAKV RDLESGVLRD VMYKGFTSEL FVPYMDPTDA WYFKTYMDAG EYGFGLQAMP LDPLNDCPRN  AYYMDGVFAA GDGVPYVRSN MVCMFERYTG DIGWRHAESP ITGMEIKEVR PKVTLVVRMA ASVANYDYIV DWEFQTDGLI RIKVGLSGIL MVKGTAYENM  NQVEGQENLF GTLLSENVIG VIHDHYITFY LDMDIDGSDN SFVNVNIKRQ ETSPGESPRK SYLKAVRNVA KTEKDAQIQL KLYDPSEFHV INPTKKTRVG  NPVGYKVVPG GTAASLLDHD DPPQKRGAFT NNQIWVTPYN RSEQWAGGLF VYQSHGEDTL DVWSERDRPI ENKDIVVWYT LGFHHIPCQE DFPIMPTVSS  SFDLKPVNFF ESNPILRAPP YFEKDLPVCR PAASA |

| Legend | | |
| --- | --- | --- |
| THISREFERE NCE | UNMATCHED | OTHERREFER ENCES |

| \| 83237.01 Da \| Protein Coverage \| \| \| --- \| --- \| --- \| \| 735 AA \| 10 AA \| **1.36 %** \| |
| --- | --- | --- | --- | --- | --- | --- |

**Data table 17**

| \| **Sequence** \| \| --- \| \| VGLSGILM^*^VK \| |
| --- | --- | --- |
| **Predicted Fragmentation Pattern**   \| **Seq** \| **#** \| **b: Δ Error** \| **b** \| **y** \| **y: Δ Error** \| **+1** \| \| --- \| --- \| --- \| --- \| --- \| --- \| --- \| \| **V** \| **1** \| **---** \| [100.076](https://tmsf.med.harvard.edu/core/www/modules/sdig/index.php?value=11/18&pvkey=&run_id=92259&search_id=93773&scans_id=92255&peptide_id=4626&charge=2&scanf=13711&mz_l=0.075999999999993&mz_h=200.076) \| --- \| **---** \| **10** \| \| **G** \| **2** \| **-0.394** \| [**157.097**](https://tmsf.med.harvard.edu/core/www/modules/sdig/index.php?value=11/18&pvkey=&run_id=92259&search_id=93773&scans_id=92255&peptide_id=4626&charge=2&scanf=13711&mz_l=57.097&mz_h=257.097) \| [**933.544**](https://tmsf.med.harvard.edu/core/www/modules/sdig/index.php?value=11/18&pvkey=&run_id=92259&search_id=93773&scans_id=92255&peptide_id=4626&charge=2&scanf=13711&mz_l=833.544&mz_h=1033.544) \| **-1.311** \| **9** \| \| **L** \| **3** \| **-0.016** \| [**270.181**](https://tmsf.med.harvard.edu/core/www/modules/sdig/index.php?value=11/18&pvkey=&run_id=92259&search_id=93773&scans_id=92255&peptide_id=4626&charge=2&scanf=13711&mz_l=170.181&mz_h=370.181) \| [**876.522**](https://tmsf.med.harvard.edu/core/www/modules/sdig/index.php?value=11/18&pvkey=&run_id=92259&search_id=93773&scans_id=92255&peptide_id=4626&charge=2&scanf=13711&mz_l=776.522&mz_h=976.522) \| **0.739** \| **8** \| \| **S** \| **4** \| **-0.910** \| [**357.213**](https://tmsf.med.harvard.edu/core/www/modules/sdig/index.php?value=11/18&pvkey=&run_id=92259&search_id=93773&scans_id=92255&peptide_id=4626&charge=2&scanf=13711&mz_l=257.213&mz_h=457.213) \| [**763.438**](https://tmsf.med.harvard.edu/core/www/modules/sdig/index.php?value=11/18&pvkey=&run_id=92259&search_id=93773&scans_id=92255&peptide_id=4626&charge=2&scanf=13711&mz_l=663.438&mz_h=863.438) \| **-0.806** \| **7** \| \| **G** \| **5** \| **1.697** \| [**414.235**](https://tmsf.med.harvard.edu/core/www/modules/sdig/index.php?value=11/18&pvkey=&run_id=92259&search_id=93773&scans_id=92255&peptide_id=4626&charge=2&scanf=13711&mz_l=314.235&mz_h=514.235) \| [**676.406**](https://tmsf.med.harvard.edu/core/www/modules/sdig/index.php?value=11/18&pvkey=&run_id=92259&search_id=93773&scans_id=92255&peptide_id=4626&charge=2&scanf=13711&mz_l=576.406&mz_h=776.406) \| **-0.481** \| **6** \| \| **I** \| **6** \| **---** \| [527.319](https://tmsf.med.harvard.edu/core/www/modules/sdig/index.php?value=11/18&pvkey=&run_id=92259&search_id=93773&scans_id=92255&peptide_id=4626&charge=2&scanf=13711&mz_l=427.319&mz_h=627.319) \| [**619.385**](https://tmsf.med.harvard.edu/core/www/modules/sdig/index.php?value=11/18&pvkey=&run_id=92259&search_id=93773&scans_id=92255&peptide_id=4626&charge=2&scanf=13711&mz_l=519.385&mz_h=719.385) \| **0.624** \| **5** \| \| **L** \| **7** \| **---** \| [640.403](https://tmsf.med.harvard.edu/core/www/modules/sdig/index.php?value=11/18&pvkey=&run_id=92259&search_id=93773&scans_id=92255&peptide_id=4626&charge=2&scanf=13711&mz_l=540.403&mz_h=740.403) \| [**506.301**](https://tmsf.med.harvard.edu/core/www/modules/sdig/index.php?value=11/18&pvkey=&run_id=92259&search_id=93773&scans_id=92255&peptide_id=4626&charge=2&scanf=13711&mz_l=406.301&mz_h=606.301) \| **-0.225** \| **4** \| \| **M^*^** \| **8** \| **0.924** \| [**787.438**](https://tmsf.med.harvard.edu/core/www/modules/sdig/index.php?value=11/18&pvkey=&run_id=92259&search_id=93773&scans_id=92255&peptide_id=4626&charge=2&scanf=13711&mz_l=687.438&mz_h=887.438) \| [**393.217**](https://tmsf.med.harvard.edu/core/www/modules/sdig/index.php?value=11/18&pvkey=&run_id=92259&search_id=93773&scans_id=92255&peptide_id=4626&charge=2&scanf=13711&mz_l=293.217&mz_h=493.217) \| **-0.553** \| **3** \| \| **V** \| **9** \| **---** \| [886.507](https://tmsf.med.harvard.edu/core/www/modules/sdig/index.php?value=11/18&pvkey=&run_id=92259&search_id=93773&scans_id=92255&peptide_id=4626&charge=2&scanf=13711&mz_l=786.507&mz_h=986.507) \| [**246.181**](https://tmsf.med.harvard.edu/core/www/modules/sdig/index.php?value=11/18&pvkey=&run_id=92259&search_id=93773&scans_id=92255&peptide_id=4626&charge=2&scanf=13711&mz_l=146.181&mz_h=346.181) \| **-0.640** \| **2** \| \| **K** \| **10** \| **---** \| --- \| [**147.113**](https://tmsf.med.harvard.edu/core/www/modules/sdig/index.php?value=11/18&pvkey=&run_id=92259&search_id=93773&scans_id=92255&peptide_id=4626&charge=2&scanf=13711&mz_l=47.113&mz_h=247.113) \| **0.025** \| **1** \| |

**Sequence no 13**

| [>tr\|A0A5E4FFS0\|A0A5E4FFS0_PRUDU PREDICTED: legumin OS=Prunus dulcis OX=3755 GN=ALMOND_2B017935 PE=3 SV=1](http://www.uniprot.org/uniprot/?query=A0A5E4FFS0_PRUDU) |
| --- |
| MAKAFVFSLC LLLVFNGCLA ARQSQLSPQN QCQLNQLQAR EPDNRIQAEA GQIETWNFNQ EDFQCAGVAA SRITIQRNGL HLPSYSNAPQ LIYIVQGRGV  LGAVFSGCPE TFEESQQSSQ QGRQQEQEQE RQQQQQGEQG RQQGQQEQQQ ERQGRQQGRQ QQEEGRQQEQ QQGQQGRPQQ QQQFRQFDRH QKTRRIREGD  VVAIPAGVAY WSYNDGDQEL VAVNLFHVSS DHNQLDQNPR KFYLAGNPEN EFNQQGQSQP RQQGEQGRPG QHQQPFGRPR QQEQQGNGNN VFSGFNTQLL  AQALNVNEET ARNLQGQNDN RNQIIQVRGN LDFVQPPRGR QEREHEERQQ EQLQQERQQQ GGQLMANGLE ETFCSLRLKE NIGNPERADI FSPRAGRIST  LNSHNLPILR FLRLSAERGF FYRNGIYSPH WNVNAHSVVY VIRGNARVQV VNENGDAILD QEVQQGQLFI VPQNHGVIQQ AGNQGFEYFA FKTEENAFIN  TLAGRTSFLR ALPDEVLANA YQISREQARQ LKYNRQETIA LSSSQQRRAV V |

| Legend | | |
| --- | --- | --- |
| THISREFERE NCE | UNMATCHED | OTHERREFER ENCES |

| \| 63013.21 Da \| Protein Coverage \| \| \| --- \| --- \| --- \| \| 551 AA \| 291 AA \| **52.81 %** \| |
| --- | --- | --- | --- | --- | --- | --- |

**Data table 18**

| \| **Sequence** \| \| --- \| \| FYLAGNPENEFNQQGQSQPR \| |
| --- | --- | --- |
| **Predicted Fragmentation Pattern**  **+1**   \| **Seq** \| **#** \| **b: Δ Error** \| **b** \| **y** \| **y: Δ Error** \| **+1** \| \| --- \| --- \| --- \| --- \| --- \| --- \| --- \| \| **F** \| **1** \| **---** \| [148.076](https://tmsf.med.harvard.edu/core/www/modules/sdig/index.php?value=18/76&pvkey=&run_id=92258&search_id=93772&scans_id=92254&peptide_id=2317&charge=3&scanf=11451&mz_l=48.076&mz_h=248.076) \| --- \| **---** \| **20** \| \| **Y** \| **2** \| **-0.722** \| [**311.139**](https://tmsf.med.harvard.edu/core/www/modules/sdig/index.php?value=18/76&pvkey=&run_id=92258&search_id=93772&scans_id=92254&peptide_id=2317&charge=3&scanf=11451&mz_l=211.139&mz_h=411.139) \| [2177.001](https://tmsf.med.harvard.edu/core/www/modules/sdig/index.php?value=18/76&pvkey=&run_id=92258&search_id=93772&scans_id=92254&peptide_id=2317&charge=3&scanf=11451&mz_l=2077.001&mz_h=2277.001) \| **---** \| **19** \| \| **L** \| **3** \| **-0.069** \| [**424.223**](https://tmsf.med.harvard.edu/core/www/modules/sdig/index.php?value=18/76&pvkey=&run_id=92258&search_id=93772&scans_id=92254&peptide_id=2317&charge=3&scanf=11451&mz_l=324.223&mz_h=524.223) \| [2013.937](https://tmsf.med.harvard.edu/core/www/modules/sdig/index.php?value=18/76&pvkey=&run_id=92258&search_id=93772&scans_id=92254&peptide_id=2317&charge=3&scanf=11451&mz_l=1913.937&mz_h=2113.937) \| **---** \| **18** \| \| **A** \| **4** \| **-0.746** \| [**495.260**](https://tmsf.med.harvard.edu/core/www/modules/sdig/index.php?value=18/76&pvkey=&run_id=92258&search_id=93772&scans_id=92254&peptide_id=2317&charge=3&scanf=11451&mz_l=395.26&mz_h=595.26) \| [1900.853](https://tmsf.med.harvard.edu/core/www/modules/sdig/index.php?value=18/76&pvkey=&run_id=92258&search_id=93772&scans_id=92254&peptide_id=2317&charge=3&scanf=11451&mz_l=1800.853&mz_h=2000.853) \| **---** \| **17** \| \| **G** \| **5** \| **-1.295** \| [**552.282**](https://tmsf.med.harvard.edu/core/www/modules/sdig/index.php?value=18/76&pvkey=&run_id=92258&search_id=93772&scans_id=92254&peptide_id=2317&charge=3&scanf=11451&mz_l=452.282&mz_h=652.282) \| [1829.816](https://tmsf.med.harvard.edu/core/www/modules/sdig/index.php?value=18/76&pvkey=&run_id=92258&search_id=93772&scans_id=92254&peptide_id=2317&charge=3&scanf=11451&mz_l=1729.816&mz_h=1929.816) \| **---** \| **16** \| \| **N** \| **6** \| **0.454** \| [**666.325**](https://tmsf.med.harvard.edu/core/www/modules/sdig/index.php?value=18/76&pvkey=&run_id=92258&search_id=93772&scans_id=92254&peptide_id=2317&charge=3&scanf=11451&mz_l=566.325&mz_h=766.325) \| [1772.795](https://tmsf.med.harvard.edu/core/www/modules/sdig/index.php?value=18/76&pvkey=&run_id=92258&search_id=93772&scans_id=92254&peptide_id=2317&charge=3&scanf=11451&mz_l=1672.795&mz_h=1872.795) \| **---** \| **15** \| \| **P** \| **7** \| **1.957** \| [**763.377**](https://tmsf.med.harvard.edu/core/www/modules/sdig/index.php?value=18/76&pvkey=&run_id=92258&search_id=93772&scans_id=92254&peptide_id=2317&charge=3&scanf=11451&mz_l=663.377&mz_h=863.377) \| [**1658.752**](https://tmsf.med.harvard.edu/core/www/modules/sdig/index.php?value=18/76&pvkey=&run_id=92258&search_id=93772&scans_id=92254&peptide_id=2317&charge=3&scanf=11451&mz_l=1558.752&mz_h=1758.752) \| **3.736** \| **14** \| \| **E** \| **8** \| **1.411** \| [**892.420**](https://tmsf.med.harvard.edu/core/www/modules/sdig/index.php?value=18/76&pvkey=&run_id=92258&search_id=93772&scans_id=92254&peptide_id=2317&charge=3&scanf=11451&mz_l=792.42&mz_h=992.42) \| [**1561.699**](https://tmsf.med.harvard.edu/core/www/modules/sdig/index.php?value=18/76&pvkey=&run_id=92258&search_id=93772&scans_id=92254&peptide_id=2317&charge=3&scanf=11451&mz_l=1461.699&mz_h=1661.699) \| **-2.266** \| **13** \| \| **N** \| **9** \| **2.081** \| [**1006.463**](https://tmsf.med.harvard.edu/core/www/modules/sdig/index.php?value=18/76&pvkey=&run_id=92258&search_id=93772&scans_id=92254&peptide_id=2317&charge=3&scanf=11451&mz_l=906.463&mz_h=1106.463) \| [**1432.656**](https://tmsf.med.harvard.edu/core/www/modules/sdig/index.php?value=18/76&pvkey=&run_id=92258&search_id=93772&scans_id=92254&peptide_id=2317&charge=3&scanf=11451&mz_l=1332.656&mz_h=1532.656) \| **0.505** \| **12** \| \| **E** \| **10** \| **-1.588** \| [**1135.505**](https://tmsf.med.harvard.edu/core/www/modules/sdig/index.php?value=18/76&pvkey=&run_id=92258&search_id=93772&scans_id=92254&peptide_id=2317&charge=3&scanf=11451&mz_l=1035.505&mz_h=1235.505) \| [**1318.613**](https://tmsf.med.harvard.edu/core/www/modules/sdig/index.php?value=18/76&pvkey=&run_id=92258&search_id=93772&scans_id=92254&peptide_id=2317&charge=3&scanf=11451&mz_l=1218.613&mz_h=1418.613) \| **-0.593** \| **11** \| \| **F** \| **11** \| **1.122** \| [**1282.574**](https://tmsf.med.harvard.edu/core/www/modules/sdig/index.php?value=18/76&pvkey=&run_id=92258&search_id=93772&scans_id=92254&peptide_id=2317&charge=3&scanf=11451&mz_l=1182.574&mz_h=1382.574) \| [**1189.571**](https://tmsf.med.harvard.edu/core/www/modules/sdig/index.php?value=18/76&pvkey=&run_id=92258&search_id=93772&scans_id=92254&peptide_id=2317&charge=3&scanf=11451&mz_l=1089.571&mz_h=1289.571) \| **0.156** \| **10** \| \| **N** \| **12** \| **6.566** \| [**1396.617**](https://tmsf.med.harvard.edu/core/www/modules/sdig/index.php?value=18/76&pvkey=&run_id=92258&search_id=93772&scans_id=92254&peptide_id=2317&charge=3&scanf=11451&mz_l=1296.617&mz_h=1496.617) \| [**1042.502**](https://tmsf.med.harvard.edu/core/www/modules/sdig/index.php?value=18/76&pvkey=&run_id=92258&search_id=93772&scans_id=92254&peptide_id=2317&charge=3&scanf=11451&mz_l=942.502&mz_h=1142.502) \| **-0.473** \| **9** \| \| **Q** \| **13** \| **---** \| [1524.675](https://tmsf.med.harvard.edu/core/www/modules/sdig/index.php?value=18/76&pvkey=&run_id=92258&search_id=93772&scans_id=92254&peptide_id=2317&charge=3&scanf=11451&mz_l=1424.675&mz_h=1624.675) \| [**928.460**](https://tmsf.med.harvard.edu/core/www/modules/sdig/index.php?value=18/76&pvkey=&run_id=92258&search_id=93772&scans_id=92254&peptide_id=2317&charge=3&scanf=11451&mz_l=828.46&mz_h=1028.46) \| **-0.115** \| **8** \| \| **Q** \| **14** \| **---** \| [1652.734](https://tmsf.med.harvard.edu/core/www/modules/sdig/index.php?value=18/76&pvkey=&run_id=92258&search_id=93772&scans_id=92254&peptide_id=2317&charge=3&scanf=11451&mz_l=1552.734&mz_h=1752.734) \| [**800.401**](https://tmsf.med.harvard.edu/core/www/modules/sdig/index.php?value=18/76&pvkey=&run_id=92258&search_id=93772&scans_id=92254&peptide_id=2317&charge=3&scanf=11451&mz_l=700.401&mz_h=900.401) \| **-0.154** \| **7** \| \| **G** \| **15** \| **---** \| [1709.755](https://tmsf.med.harvard.edu/core/www/modules/sdig/index.php?value=18/76&pvkey=&run_id=92258&search_id=93772&scans_id=92254&peptide_id=2317&charge=3&scanf=11451&mz_l=1609.755&mz_h=1809.755) \| [**672.342**](https://tmsf.med.harvard.edu/core/www/modules/sdig/index.php?value=18/76&pvkey=&run_id=92258&search_id=93772&scans_id=92254&peptide_id=2317&charge=3&scanf=11451&mz_l=572.342&mz_h=772.342) \| **-0.117** \| **6** \| \| **Q** \| **16** \| **---** \| [1837.814](https://tmsf.med.harvard.edu/core/www/modules/sdig/index.php?value=18/76&pvkey=&run_id=92258&search_id=93772&scans_id=92254&peptide_id=2317&charge=3&scanf=11451&mz_l=1737.814&mz_h=1937.814) \| [**615.321**](https://tmsf.med.harvard.edu/core/www/modules/sdig/index.php?value=18/76&pvkey=&run_id=92258&search_id=93772&scans_id=92254&peptide_id=2317&charge=3&scanf=11451&mz_l=515.321&mz_h=715.321) \| **0.037** \| **5** \| \| **S** \| **17** \| **---** \| [1924.846](https://tmsf.med.harvard.edu/core/www/modules/sdig/index.php?value=18/76&pvkey=&run_id=92258&search_id=93772&scans_id=92254&peptide_id=2317&charge=3&scanf=11451&mz_l=1824.846&mz_h=2024.846) \| [**487.262**](https://tmsf.med.harvard.edu/core/www/modules/sdig/index.php?value=18/76&pvkey=&run_id=92258&search_id=93772&scans_id=92254&peptide_id=2317&charge=3&scanf=11451&mz_l=387.262&mz_h=587.262) \| **-0.863** \| **4** \| \| **Q** \| **18** \| **---** \| [2052.905](https://tmsf.med.harvard.edu/core/www/modules/sdig/index.php?value=18/76&pvkey=&run_id=92258&search_id=93772&scans_id=92254&peptide_id=2317&charge=3&scanf=11451&mz_l=1952.905&mz_h=2152.905) \| [**400.230**](https://tmsf.med.harvard.edu/core/www/modules/sdig/index.php?value=18/76&pvkey=&run_id=92258&search_id=93772&scans_id=92254&peptide_id=2317&charge=3&scanf=11451&mz_l=300.23&mz_h=500.23) \| **-0.554** \| **3** \| \| **P** \| **19** \| **---** \| [2149.957](https://tmsf.med.harvard.edu/core/www/modules/sdig/index.php?value=18/76&pvkey=&run_id=92258&search_id=93772&scans_id=92254&peptide_id=2317&charge=3&scanf=11451&mz_l=2049.957&mz_h=2249.957) \| [**272.172**](https://tmsf.med.harvard.edu/core/www/modules/sdig/index.php?value=18/76&pvkey=&run_id=92258&search_id=93772&scans_id=92254&peptide_id=2317&charge=3&scanf=11451&mz_l=172.172&mz_h=372.172) \| **0.022** \| **2** \| \| **R** \| **20** \| **---** \| --- \| [**175.119**](https://tmsf.med.harvard.edu/core/www/modules/sdig/index.php?value=18/76&pvkey=&run_id=92258&search_id=93772&scans_id=92254&peptide_id=2317&charge=3&scanf=11451&mz_l=75.119&mz_h=275.119) \| **-0.581** \| **1** \| \|  \|  \|  \|  \|  \|  \|  \| |

| \| **Sequence** \| \| --- \| \| TAVQLYSDYMK \| |
| --- | --- | --- |
| **Predicted Fragmentation Pattern**   \| **Seq** \| **#** \| **b: Δ Error** \| **b** \| **y** \| **y: Δ Error** \| **+1** \| \| --- \| --- \| --- \| --- \| --- \| --- \| --- \| \| **T** \| **1** \| **---** \| [102.055](https://tmsf.med.harvard.edu/core/www/modules/sdig/index.php?run_id=92259&search_id=93773&scans_id=92255&peptide_id=1513&scanf=10660&charge=2&value=R.TAVQLYSDYMK.S&mz_l=2.055&mz_h=202.055) \| --- \| **---** \| **11** \| \| **A** \| **2** \| **0.107** \| [**173.092**](https://tmsf.med.harvard.edu/core/www/modules/sdig/index.php?run_id=92259&search_id=93773&scans_id=92255&peptide_id=1513&scanf=10660&charge=2&value=R.TAVQLYSDYMK.S&mz_l=73.092&mz_h=273.092) \| [1217.587](https://tmsf.med.harvard.edu/core/www/modules/sdig/index.php?run_id=92259&search_id=93773&scans_id=92255&peptide_id=1513&scanf=10660&charge=2&value=R.TAVQLYSDYMK.S&mz_l=1117.587&mz_h=1317.587) \| **---** \| **10** \| \| **V** \| **3** \| **-0.188** \| [**272.160**](https://tmsf.med.harvard.edu/core/www/modules/sdig/index.php?run_id=92259&search_id=93773&scans_id=92255&peptide_id=1513&scanf=10660&charge=2&value=R.TAVQLYSDYMK.S&mz_l=172.16&mz_h=372.16) \| [**1146.550**](https://tmsf.med.harvard.edu/core/www/modules/sdig/index.php?run_id=92259&search_id=93773&scans_id=92255&peptide_id=1513&scanf=10660&charge=2&value=R.TAVQLYSDYMK.S&mz_l=1046.55&mz_h=1246.55) \| **-0.679** \| **9** \| \| **Q** \| **4** \| **-0.393** \| [**400.219**](https://tmsf.med.harvard.edu/core/www/modules/sdig/index.php?run_id=92259&search_id=93773&scans_id=92255&peptide_id=1513&scanf=10660&charge=2&value=R.TAVQLYSDYMK.S&mz_l=300.219&mz_h=500.219) \| [**1047.482**](https://tmsf.med.harvard.edu/core/www/modules/sdig/index.php?run_id=92259&search_id=93773&scans_id=92255&peptide_id=1513&scanf=10660&charge=2&value=R.TAVQLYSDYMK.S&mz_l=947.482&mz_h=1147.482) \| **0.474** \| **8** \| \| **L** \| **5** \| **0.193** \| [**513.303**](https://tmsf.med.harvard.edu/core/www/modules/sdig/index.php?run_id=92259&search_id=93773&scans_id=92255&peptide_id=1513&scanf=10660&charge=2&value=R.TAVQLYSDYMK.S&mz_l=413.303&mz_h=613.303) \| [**919.423**](https://tmsf.med.harvard.edu/core/www/modules/sdig/index.php?run_id=92259&search_id=93773&scans_id=92255&peptide_id=1513&scanf=10660&charge=2&value=R.TAVQLYSDYMK.S&mz_l=819.423&mz_h=1019.423) \| **-1.403** \| **7** \| \| **Y** \| **6** \| **---** \| [676.366](https://tmsf.med.harvard.edu/core/www/modules/sdig/index.php?run_id=92259&search_id=93773&scans_id=92255&peptide_id=1513&scanf=10660&charge=2&value=R.TAVQLYSDYMK.S&mz_l=576.366&mz_h=776.366) \| [**806.339**](https://tmsf.med.harvard.edu/core/www/modules/sdig/index.php?run_id=92259&search_id=93773&scans_id=92255&peptide_id=1513&scanf=10660&charge=2&value=R.TAVQLYSDYMK.S&mz_l=706.339&mz_h=906.339) \| **-0.668** \| **6** \| \| **S** \| **7** \| **---** \| [763.398](https://tmsf.med.harvard.edu/core/www/modules/sdig/index.php?run_id=92259&search_id=93773&scans_id=92255&peptide_id=1513&scanf=10660&charge=2&value=R.TAVQLYSDYMK.S&mz_l=663.398&mz_h=863.398) \| [**643.276**](https://tmsf.med.harvard.edu/core/www/modules/sdig/index.php?run_id=92259&search_id=93773&scans_id=92255&peptide_id=1513&scanf=10660&charge=2&value=R.TAVQLYSDYMK.S&mz_l=543.276&mz_h=743.276) \| **-1.922** \| **5** \| \| **D** \| **8** \| **---** \| [878.425](https://tmsf.med.harvard.edu/core/www/modules/sdig/index.php?run_id=92259&search_id=93773&scans_id=92255&peptide_id=1513&scanf=10660&charge=2&value=R.TAVQLYSDYMK.S&mz_l=778.425&mz_h=978.425) \| [**556.244**](https://tmsf.med.harvard.edu/core/www/modules/sdig/index.php?run_id=92259&search_id=93773&scans_id=92255&peptide_id=1513&scanf=10660&charge=2&value=R.TAVQLYSDYMK.S&mz_l=456.244&mz_h=656.244) \| **0.933** \| **4** \| \| **Y** \| **9** \| **---** \| [1041.489](https://tmsf.med.harvard.edu/core/www/modules/sdig/index.php?run_id=92259&search_id=93773&scans_id=92255&peptide_id=1513&scanf=10660&charge=2&value=R.TAVQLYSDYMK.S&mz_l=941.489&mz_h=1141.489) \| [**441.217**](https://tmsf.med.harvard.edu/core/www/modules/sdig/index.php?run_id=92259&search_id=93773&scans_id=92255&peptide_id=1513&scanf=10660&charge=2&value=R.TAVQLYSDYMK.S&mz_l=341.217&mz_h=541.217) \| **-0.147** \| **3** \| \| **M** \| **10** \| **---** \| [1172.529](https://tmsf.med.harvard.edu/core/www/modules/sdig/index.php?run_id=92259&search_id=93773&scans_id=92255&peptide_id=1513&scanf=10660&charge=2&value=R.TAVQLYSDYMK.S&mz_l=1072.529&mz_h=1272.529) \| [278.153](https://tmsf.med.harvard.edu/core/www/modules/sdig/index.php?run_id=92259&search_id=93773&scans_id=92255&peptide_id=1513&scanf=10660&charge=2&value=R.TAVQLYSDYMK.S&mz_l=178.153&mz_h=378.153) \| **---** \| **2** \| \| **K** \| **11** \| **---** \| --- \| [**147.113**](https://tmsf.med.harvard.edu/core/www/modules/sdig/index.php?run_id=92259&search_id=93773&scans_id=92255&peptide_id=1513&scanf=10660&charge=2&value=R.TAVQLYSDYMK.S&mz_l=47.113&mz_h=247.113) \| **0.544** \| **1** \| |

**Sequence no 14**

| [>tr\|A0A1U8HRB5\|A0A1U8HRB5_GOSHI Beta-amylase OS=Gossypium hirsutum OX=3635 GN=LOC107886910 PE=3 SV=1](http://www.uniprot.org/uniprot/?query=A0A1U8HRB5_GOSHI) |
| --- |
| MTSIIRSTQG GCWGSDRLRL RLYGSVFDLA VMPLIDRVES LQGVIRKVKR IEAMGQTGRS NYVDVTKFPK AGTSKLSTDE MPEAPTAPST YNEKILANYV  PVYVMLPLGV ISNDNVFEDQ PKMEKQLKEL RAAGVDGVMV DVWWGIVESK GPKQYDWSAY RSLFALVQEC GLKLQAIMSF HRCGGNVGDE VTIPLPQWVL  DIGETDPDIF YTNRKGNRNK EYLTIGVDNQ PLFDGRTAVQ LYSDYMKSFR ENMSDFIEAG LIIDIEVGLG PAGELRYPSY TGTQGWVFPG IGEFQCYDKY  LEAEFKEAAT GAGHPEWGLP DNAGEYNDTP GSTEFFGLNG TYMTEKGKFF LTWYSNKLIN HGDEILDEAN RAFLGCKVKL AAKVAGIHWW YKSPSHAAEL  TSGYYNLKDR DGYRPIARML SRHYAIFNFT CLEMRDSEQR ADAKCGPQEL VQQVLSGGWR EEVEVAGENA LSRYDSNGYN QMLLNARPSG ISREGLPKQI  MYGVTYLRLS DELLKDKNFK IFKTFVKKMH AHQDYCADLG MYNHQIGPLE RSKPKLVMED LLEATKPMEP FPWGEETDMK VDDFDGVLAN LIRKLFSLFK |

| Legend | | |
| --- | --- | --- |
| THISREFERE NCE | UNMATCHED | OTHERREFER ENCES |

| \| 67809.78 Da \| Protein Coverage \| \| \| --- \| --- \| --- \| \| 600 AA \| 11 AA \| **1.83 %** \| |
| --- | --- | --- | --- | --- | --- | --- |

**Data table 19**

| \| **Sequence** \| \| --- \| \| TAVQLYSDYMK \| |
| --- | --- | --- |
| **Predicted Fragmentation Pattern**   \| **Seq** \| **#** \| **b: Δ Error** \| **b** \| **y** \| **y: Δ Error** \| **+1** \| \| --- \| --- \| --- \| --- \| --- \| --- \| --- \| \| **T** \| **1** \| **---** \| [102.055](https://tmsf.med.harvard.edu/core/www/modules/sdig/index.php?run_id=92259&search_id=93773&scans_id=92255&peptide_id=1513&scanf=10660&charge=2&value=R.TAVQLYSDYMK.S&mz_l=2.055&mz_h=202.055) \| --- \| **---** \| **11** \| \| **A** \| **2** \| **0.107** \| [**173.092**](https://tmsf.med.harvard.edu/core/www/modules/sdig/index.php?run_id=92259&search_id=93773&scans_id=92255&peptide_id=1513&scanf=10660&charge=2&value=R.TAVQLYSDYMK.S&mz_l=73.092&mz_h=273.092) \| [1217.587](https://tmsf.med.harvard.edu/core/www/modules/sdig/index.php?run_id=92259&search_id=93773&scans_id=92255&peptide_id=1513&scanf=10660&charge=2&value=R.TAVQLYSDYMK.S&mz_l=1117.587&mz_h=1317.587) \| **---** \| **10** \| \| **V** \| **3** \| **-0.188** \| [**272.160**](https://tmsf.med.harvard.edu/core/www/modules/sdig/index.php?run_id=92259&search_id=93773&scans_id=92255&peptide_id=1513&scanf=10660&charge=2&value=R.TAVQLYSDYMK.S&mz_l=172.16&mz_h=372.16) \| [**1146.550**](https://tmsf.med.harvard.edu/core/www/modules/sdig/index.php?run_id=92259&search_id=93773&scans_id=92255&peptide_id=1513&scanf=10660&charge=2&value=R.TAVQLYSDYMK.S&mz_l=1046.55&mz_h=1246.55) \| **-0.679** \| **9** \| \| **Q** \| **4** \| **-0.393** \| [**400.219**](https://tmsf.med.harvard.edu/core/www/modules/sdig/index.php?run_id=92259&search_id=93773&scans_id=92255&peptide_id=1513&scanf=10660&charge=2&value=R.TAVQLYSDYMK.S&mz_l=300.219&mz_h=500.219) \| [**1047.482**](https://tmsf.med.harvard.edu/core/www/modules/sdig/index.php?run_id=92259&search_id=93773&scans_id=92255&peptide_id=1513&scanf=10660&charge=2&value=R.TAVQLYSDYMK.S&mz_l=947.482&mz_h=1147.482) \| **0.474** \| **8** \| \| **L** \| **5** \| **0.193** \| [**513.303**](https://tmsf.med.harvard.edu/core/www/modules/sdig/index.php?run_id=92259&search_id=93773&scans_id=92255&peptide_id=1513&scanf=10660&charge=2&value=R.TAVQLYSDYMK.S&mz_l=413.303&mz_h=613.303) \| [**919.423**](https://tmsf.med.harvard.edu/core/www/modules/sdig/index.php?run_id=92259&search_id=93773&scans_id=92255&peptide_id=1513&scanf=10660&charge=2&value=R.TAVQLYSDYMK.S&mz_l=819.423&mz_h=1019.423) \| **-1.403** \| **7** \| \| **Y** \| **6** \| **---** \| [676.366](https://tmsf.med.harvard.edu/core/www/modules/sdig/index.php?run_id=92259&search_id=93773&scans_id=92255&peptide_id=1513&scanf=10660&charge=2&value=R.TAVQLYSDYMK.S&mz_l=576.366&mz_h=776.366) \| [**806.339**](https://tmsf.med.harvard.edu/core/www/modules/sdig/index.php?run_id=92259&search_id=93773&scans_id=92255&peptide_id=1513&scanf=10660&charge=2&value=R.TAVQLYSDYMK.S&mz_l=706.339&mz_h=906.339) \| **-0.668** \| **6** \| \| **S** \| **7** \| **---** \| [763.398](https://tmsf.med.harvard.edu/core/www/modules/sdig/index.php?run_id=92259&search_id=93773&scans_id=92255&peptide_id=1513&scanf=10660&charge=2&value=R.TAVQLYSDYMK.S&mz_l=663.398&mz_h=863.398) \| [**643.276**](https://tmsf.med.harvard.edu/core/www/modules/sdig/index.php?run_id=92259&search_id=93773&scans_id=92255&peptide_id=1513&scanf=10660&charge=2&value=R.TAVQLYSDYMK.S&mz_l=543.276&mz_h=743.276) \| **-1.922** \| **5** \| \| **D** \| **8** \| **---** \| [878.425](https://tmsf.med.harvard.edu/core/www/modules/sdig/index.php?run_id=92259&search_id=93773&scans_id=92255&peptide_id=1513&scanf=10660&charge=2&value=R.TAVQLYSDYMK.S&mz_l=778.425&mz_h=978.425) \| [**556.244**](https://tmsf.med.harvard.edu/core/www/modules/sdig/index.php?run_id=92259&search_id=93773&scans_id=92255&peptide_id=1513&scanf=10660&charge=2&value=R.TAVQLYSDYMK.S&mz_l=456.244&mz_h=656.244) \| **0.933** \| **4** \| \| **Y** \| **9** \| **---** \| [1041.489](https://tmsf.med.harvard.edu/core/www/modules/sdig/index.php?run_id=92259&search_id=93773&scans_id=92255&peptide_id=1513&scanf=10660&charge=2&value=R.TAVQLYSDYMK.S&mz_l=941.489&mz_h=1141.489) \| [**441.217**](https://tmsf.med.harvard.edu/core/www/modules/sdig/index.php?run_id=92259&search_id=93773&scans_id=92255&peptide_id=1513&scanf=10660&charge=2&value=R.TAVQLYSDYMK.S&mz_l=341.217&mz_h=541.217) \| **-0.147** \| **3** \| \| **M** \| **10** \| **---** \| [1172.529](https://tmsf.med.harvard.edu/core/www/modules/sdig/index.php?run_id=92259&search_id=93773&scans_id=92255&peptide_id=1513&scanf=10660&charge=2&value=R.TAVQLYSDYMK.S&mz_l=1072.529&mz_h=1272.529) \| [278.153](https://tmsf.med.harvard.edu/core/www/modules/sdig/index.php?run_id=92259&search_id=93773&scans_id=92255&peptide_id=1513&scanf=10660&charge=2&value=R.TAVQLYSDYMK.S&mz_l=178.153&mz_h=378.153) \| **---** \| **2** \| \| **K** \| **11** \| **---** \| --- \| [**147.113**](https://tmsf.med.harvard.edu/core/www/modules/sdig/index.php?run_id=92259&search_id=93773&scans_id=92255&peptide_id=1513&scanf=10660&charge=2&value=R.TAVQLYSDYMK.S&mz_l=47.113&mz_h=247.113) \| **0.544** \| **1** \| |

**Sequence no 15**

| [>tr\|A0A5E4EDY0\|A0A5E4EDY0_PRUDU Beta-glucosidase OS=Prunus dulcis OX=3755 GN=ALMOND_2B002417 PE=3 SV=1](http://www.uniprot.org/uniprot/?query=A0A5E4EDY0_PRUDU) |
| --- |
| MAKKDCLKEY GEADEVSRSD FPPKFVFGVA TSAYQVEGAC NEGGRGPSIW DAFSHSKGTI IDGSNGDVAV DQYHRYKEDV ELIAKLGFDA YRFSISWSRI  FPDGLGTKVN EEGISYYNNV INNLLEKGIQ PYVTLYHWDL PLYLHENMGG WLNKKIVDYF SVYADTCFAS FGDRVKDWIT INEPLQTAIN GYGVGIFAPG  RHECSSTEPY LVAHHQLLAH AAAVSIYRSK YKDKQGGQVG LTVDCEWAEA NSDKIEDKIA AARRLDFQLG WFLDPIYYGE YPKAMRERLG DRLPIFSEED  KELLENSLDF VGLNHYTSRF IAHVRESPED GDFYKSQEME RIAEWEGGEA IGEKAASEWL YVVPWGIHKV LNYIAQRYNN PAIYVTENGM DDEDNDTSPL  HEMLDDNLRV SYFKRYLSAV ANAIRDGADV RGYFAWSLLD NFEWAQGYTK RFGLVYIDYK NELSRHPKSS AYWFLRFFKA GEGKHDKEQ |

| Legend | | |
| --- | --- | --- |
| THISREFERE NCE | UNMATCHED | OTHERREFER ENCES |

| \| 55925.38 Da \| Protein Coverage \| \| \| --- \| --- \| --- \| \| 489 AA \| 7 AA \| **1.43 %** \| |
| --- | --- | --- | --- | --- | --- | --- |

**Data no 20**

| \| **Sequence** \| \| --- \| \| FSISWSR \| |
| --- | --- | --- |
| **Predicted Fragmentation Pattern**   \| **Seq** \| **#** \| **b: Δ Error** \| **b** \| **y** \| **y: Δ Error** \| **+1** \| \| --- \| --- \| --- \| --- \| --- \| --- \| --- \| \| **F** \| **1** \| **---** \| [148.076](https://tmsf.med.harvard.edu/core/www/modules/sdig/index.php?run_id=92258&search_id=93772&scans_id=92254&peptide_id=2113&scanf=11252&charge=2&value=R.FSISWSR.I&mz_l=48.076&mz_h=248.076) \| --- \| **---** \| **7** \| \| **S** \| **2** \| **-0.287** \| [**235.108**](https://tmsf.med.harvard.edu/core/www/modules/sdig/index.php?run_id=92258&search_id=93772&scans_id=92254&peptide_id=2113&scanf=11252&charge=2&value=R.FSISWSR.I&mz_l=135.108&mz_h=335.108) \| [**735.378**](https://tmsf.med.harvard.edu/core/www/modules/sdig/index.php?run_id=92258&search_id=93772&scans_id=92254&peptide_id=2113&scanf=11252&charge=2&value=R.FSISWSR.I&mz_l=635.378&mz_h=835.378) \| **-0.743** \| **6** \| \| **I** \| **3** \| **---** \| [348.192](https://tmsf.med.harvard.edu/core/www/modules/sdig/index.php?run_id=92258&search_id=93772&scans_id=92254&peptide_id=2113&scanf=11252&charge=2&value=R.FSISWSR.I&mz_l=248.192&mz_h=448.192) \| [**648.346**](https://tmsf.med.harvard.edu/core/www/modules/sdig/index.php?run_id=92258&search_id=93772&scans_id=92254&peptide_id=2113&scanf=11252&charge=2&value=R.FSISWSR.I&mz_l=548.346&mz_h=748.346) \| **-0.301** \| **5** \| \| **S** \| **4** \| **---** \| [435.224](https://tmsf.med.harvard.edu/core/www/modules/sdig/index.php?run_id=92258&search_id=93772&scans_id=92254&peptide_id=2113&scanf=11252&charge=2&value=R.FSISWSR.I&mz_l=335.224&mz_h=535.224) \| [**535.262**](https://tmsf.med.harvard.edu/core/www/modules/sdig/index.php?run_id=92258&search_id=93772&scans_id=92254&peptide_id=2113&scanf=11252&charge=2&value=R.FSISWSR.I&mz_l=435.262&mz_h=635.262) \| **-0.443** \| **4** \| \| **W** \| **5** \| **---** \| [621.303](https://tmsf.med.harvard.edu/core/www/modules/sdig/index.php?run_id=92258&search_id=93772&scans_id=92254&peptide_id=2113&scanf=11252&charge=2&value=R.FSISWSR.I&mz_l=521.303&mz_h=721.303) \| [**448.230**](https://tmsf.med.harvard.edu/core/www/modules/sdig/index.php?run_id=92258&search_id=93772&scans_id=92254&peptide_id=2113&scanf=11252&charge=2&value=R.FSISWSR.I&mz_l=348.23&mz_h=548.23) \| **-0.563** \| **3** \| \| **S** \| **6** \| **---** \| [708.335](https://tmsf.med.harvard.edu/core/www/modules/sdig/index.php?run_id=92258&search_id=93772&scans_id=92254&peptide_id=2113&scanf=11252&charge=2&value=R.FSISWSR.I&mz_l=608.335&mz_h=808.335) \| [**262.151**](https://tmsf.med.harvard.edu/core/www/modules/sdig/index.php?run_id=92258&search_id=93772&scans_id=92254&peptide_id=2113&scanf=11252&charge=2&value=R.FSISWSR.I&mz_l=162.151&mz_h=362.151) \| **0.775** \| **2** \| \| **R** \| **7** \| **---** \| --- \| [**175.119**](https://tmsf.med.harvard.edu/core/www/modules/sdig/index.php?run_id=92258&search_id=93772&scans_id=92254&peptide_id=2113&scanf=11252&charge=2&value=R.FSISWSR.I&mz_l=75.119&mz_h=275.119) \| **0.029** \| **1** \| |

**Sequence no 16**

| [>tr\|A0A4Y1QQ02\|A0A4Y1QQ02_PRUDU Glyceraldehyde-3-phosphate dehydrogenase OS=Prunus dulcis OX=3755 GN=ALMOND_2B030279 PE=3 SV=1](http://www.uniprot.org/uniprot/?query=A0A4Y1QQ02_PRUDU) |
| --- |
| MGSDKKIKIG INGFGRIGRL VARVALQRDD VELVAVNDPF ITTDYMTYMF KYDTVHGPWK HHELKVKDSK TLLFGEKPVA VFGIRNPEEI PWGEAGADFI  VESTGVFTDK DKAAFHLKGG AKKVIISAPS KDAPMFVVGV NEKEYKPDID ILSNASCTTN CLAPLAKVIN DRFGIVEGLM TTVHSITATQ KTVDGPSSKD  WRGGRAASFN IIPSSTGAAK AVGKVLPQLN GKLTGMSFRV PTVDVSVVDL TVRIEKKATY EQIKAAIKEE SEGKLKGILG YTEDDVVSTD FIGDSRSSIF  DAKAGIALND NFVKLVSWYD NEWGYSSRVV DLIVHVASTL |

| Legend | | |
| --- | --- | --- |
| THISREFERE NCE | UNMATCHED | OTHERREFER ENCES |

| \| 36997.28 Da \| Protein Coverage \| \| \| --- \| --- \| --- \| \| 340 AA \| 55 AA \| **16.18 %** \| |
| --- | --- | --- | --- | --- | --- | --- |

**Data table 21**

| \| **Sequence** \| \| --- \| \| FGIVEGLMTTVHSITATQK \| |
| --- | --- | --- |
| **Predicted Fragmentation Pattern**  **+1**   \| **Seq** \| **#** \| **b: Δ Error** \| **b** \| **y** \| **y: Δ Error** \| **+1** \| \| --- \| --- \| --- \| --- \| --- \| --- \| --- \| \| **F** \| **1** \| **---** \| [148.076](https://tmsf.med.harvard.edu/core/www/modules/sdig/index.php?value=22/72&pvkey=&run_id=92258&search_id=93772&scans_id=92254&peptide_id=7640&charge=3&scanf=16656&mz_l=48.076&mz_h=248.076) \| --- \| **---** \| **19** \| \| **G** \| **2** \| **-0.079** \| [**205.097**](https://tmsf.med.harvard.edu/core/www/modules/sdig/index.php?value=22/72&pvkey=&run_id=92258&search_id=93772&scans_id=92254&peptide_id=7640&charge=3&scanf=16656&mz_l=105.097&mz_h=305.097) \| [1886.005](https://tmsf.med.harvard.edu/core/www/modules/sdig/index.php?value=22/72&pvkey=&run_id=92258&search_id=93772&scans_id=92254&peptide_id=7640&charge=3&scanf=16656&mz_l=1786.005&mz_h=1986.005) \| **---** \| **18** \| \| **I** \| **3** \| **-0.493** \| [**318.181**](https://tmsf.med.harvard.edu/core/www/modules/sdig/index.php?value=22/72&pvkey=&run_id=92258&search_id=93772&scans_id=92254&peptide_id=7640&charge=3&scanf=16656&mz_l=218.181&mz_h=418.181) \| [1828.984](https://tmsf.med.harvard.edu/core/www/modules/sdig/index.php?value=22/72&pvkey=&run_id=92258&search_id=93772&scans_id=92254&peptide_id=7640&charge=3&scanf=16656&mz_l=1728.984&mz_h=1928.984) \| **---** \| **17** \| \| **V** \| **4** \| **-0.580** \| [**417.250**](https://tmsf.med.harvard.edu/core/www/modules/sdig/index.php?value=22/72&pvkey=&run_id=92258&search_id=93772&scans_id=92254&peptide_id=7640&charge=3&scanf=16656&mz_l=317.25&mz_h=517.25) \| [1715.900](https://tmsf.med.harvard.edu/core/www/modules/sdig/index.php?value=22/72&pvkey=&run_id=92258&search_id=93772&scans_id=92254&peptide_id=7640&charge=3&scanf=16656&mz_l=1615.9&mz_h=1815.9) \| **---** \| **16** \| \| **E** \| **5** \| **-2.325** \| [**546.292**](https://tmsf.med.harvard.edu/core/www/modules/sdig/index.php?value=22/72&pvkey=&run_id=92258&search_id=93772&scans_id=92254&peptide_id=7640&charge=3&scanf=16656&mz_l=446.292&mz_h=646.292) \| [**1616.831**](https://tmsf.med.harvard.edu/core/www/modules/sdig/index.php?value=22/72&pvkey=&run_id=92258&search_id=93772&scans_id=92254&peptide_id=7640&charge=3&scanf=16656&mz_l=1516.831&mz_h=1716.831) \| **-0.035** \| **15** \| \| **G** \| **6** \| **-0.250** \| [**603.314**](https://tmsf.med.harvard.edu/core/www/modules/sdig/index.php?value=22/72&pvkey=&run_id=92258&search_id=93772&scans_id=92254&peptide_id=7640&charge=3&scanf=16656&mz_l=503.314&mz_h=703.314) \| [**1487.789**](https://tmsf.med.harvard.edu/core/www/modules/sdig/index.php?value=22/72&pvkey=&run_id=92258&search_id=93772&scans_id=92254&peptide_id=7640&charge=3&scanf=16656&mz_l=1387.789&mz_h=1587.789) \| **-1.767** \| **14** \| \| **L** \| **7** \| **-5.263** \| [**716.398**](https://tmsf.med.harvard.edu/core/www/modules/sdig/index.php?value=22/72&pvkey=&run_id=92258&search_id=93772&scans_id=92254&peptide_id=7640&charge=3&scanf=16656&mz_l=616.398&mz_h=816.398) \| [1430.767](https://tmsf.med.harvard.edu/core/www/modules/sdig/index.php?value=22/72&pvkey=&run_id=92258&search_id=93772&scans_id=92254&peptide_id=7640&charge=3&scanf=16656&mz_l=1330.767&mz_h=1530.767) \| **---** \| **13** \| \| **M** \| **8** \| **---** \| [847.438](https://tmsf.med.harvard.edu/core/www/modules/sdig/index.php?value=22/72&pvkey=&run_id=92258&search_id=93772&scans_id=92254&peptide_id=7640&charge=3&scanf=16656&mz_l=747.438&mz_h=947.438) \| [**1317.683**](https://tmsf.med.harvard.edu/core/www/modules/sdig/index.php?value=22/72&pvkey=&run_id=92258&search_id=93772&scans_id=92254&peptide_id=7640&charge=3&scanf=16656&mz_l=1217.683&mz_h=1417.683) \| **-0.561** \| **12** \| \| **T** \| **9** \| **---** \| [948.486](https://tmsf.med.harvard.edu/core/www/modules/sdig/index.php?value=22/72&pvkey=&run_id=92258&search_id=93772&scans_id=92254&peptide_id=7640&charge=3&scanf=16656&mz_l=848.486&mz_h=1048.486) \| [**1186.643**](https://tmsf.med.harvard.edu/core/www/modules/sdig/index.php?value=22/72&pvkey=&run_id=92258&search_id=93772&scans_id=92254&peptide_id=7640&charge=3&scanf=16656&mz_l=1086.643&mz_h=1286.643) \| **0.575** \| **11** \| \| **T** \| **10** \| **---** \| [1049.534](https://tmsf.med.harvard.edu/core/www/modules/sdig/index.php?value=22/72&pvkey=&run_id=92258&search_id=93772&scans_id=92254&peptide_id=7640&charge=3&scanf=16656&mz_l=949.534&mz_h=1149.534) \| [**1085.595**](https://tmsf.med.harvard.edu/core/www/modules/sdig/index.php?value=22/72&pvkey=&run_id=92258&search_id=93772&scans_id=92254&peptide_id=7640&charge=3&scanf=16656&mz_l=985.595&mz_h=1185.595) \| **-0.205** \| **10** \| \| **V** \| **11** \| **---** \| [1148.602](https://tmsf.med.harvard.edu/core/www/modules/sdig/index.php?value=22/72&pvkey=&run_id=92258&search_id=93772&scans_id=92254&peptide_id=7640&charge=3&scanf=16656&mz_l=1048.602&mz_h=1248.602) \| [**984.547**](https://tmsf.med.harvard.edu/core/www/modules/sdig/index.php?value=22/72&pvkey=&run_id=92258&search_id=93772&scans_id=92254&peptide_id=7640&charge=3&scanf=16656&mz_l=884.547&mz_h=1084.547) \| **2.078** \| **9** \| \| **H** \| **12** \| **---** \| [1285.661](https://tmsf.med.harvard.edu/core/www/modules/sdig/index.php?value=22/72&pvkey=&run_id=92258&search_id=93772&scans_id=92254&peptide_id=7640&charge=3&scanf=16656&mz_l=1185.661&mz_h=1385.661) \| [**885.479**](https://tmsf.med.harvard.edu/core/www/modules/sdig/index.php?value=22/72&pvkey=&run_id=92258&search_id=93772&scans_id=92254&peptide_id=7640&charge=3&scanf=16656&mz_l=785.479&mz_h=985.479) \| **0.235** \| **8** \| \| **S** \| **13** \| **---** \| [1372.693](https://tmsf.med.harvard.edu/core/www/modules/sdig/index.php?value=22/72&pvkey=&run_id=92258&search_id=93772&scans_id=92254&peptide_id=7640&charge=3&scanf=16656&mz_l=1272.693&mz_h=1472.693) \| [**748.420**](https://tmsf.med.harvard.edu/core/www/modules/sdig/index.php?value=22/72&pvkey=&run_id=92258&search_id=93772&scans_id=92254&peptide_id=7640&charge=3&scanf=16656&mz_l=648.42&mz_h=848.42) \| **0.458** \| **7** \| \| **I** \| **14** \| **---** \| [1485.777](https://tmsf.med.harvard.edu/core/www/modules/sdig/index.php?value=22/72&pvkey=&run_id=92258&search_id=93772&scans_id=92254&peptide_id=7640&charge=3&scanf=16656&mz_l=1385.777&mz_h=1585.777) \| [**661.388**](https://tmsf.med.harvard.edu/core/www/modules/sdig/index.php?value=22/72&pvkey=&run_id=92258&search_id=93772&scans_id=92254&peptide_id=7640&charge=3&scanf=16656&mz_l=561.388&mz_h=761.388) \| **2.618** \| **6** \| \| **T** \| **15** \| **---** \| [1586.825](https://tmsf.med.harvard.edu/core/www/modules/sdig/index.php?value=22/72&pvkey=&run_id=92258&search_id=93772&scans_id=92254&peptide_id=7640&charge=3&scanf=16656&mz_l=1486.825&mz_h=1686.825) \| [**548.304**](https://tmsf.med.harvard.edu/core/www/modules/sdig/index.php?value=22/72&pvkey=&run_id=92258&search_id=93772&scans_id=92254&peptide_id=7640&charge=3&scanf=16656&mz_l=448.304&mz_h=648.304) \| **-2.151** \| **5** \| \| **A** \| **16** \| **---** \| [1657.862](https://tmsf.med.harvard.edu/core/www/modules/sdig/index.php?value=22/72&pvkey=&run_id=92258&search_id=93772&scans_id=92254&peptide_id=7640&charge=3&scanf=16656&mz_l=1557.862&mz_h=1757.862) \| [**447.256**](https://tmsf.med.harvard.edu/core/www/modules/sdig/index.php?value=22/72&pvkey=&run_id=92258&search_id=93772&scans_id=92254&peptide_id=7640&charge=3&scanf=16656&mz_l=347.256&mz_h=547.256) \| **0.046** \| **4** \| \| **T** \| **17** \| **---** \| [1758.909](https://tmsf.med.harvard.edu/core/www/modules/sdig/index.php?value=22/72&pvkey=&run_id=92258&search_id=93772&scans_id=92254&peptide_id=7640&charge=3&scanf=16656&mz_l=1658.909&mz_h=1858.909) \| [**376.219**](https://tmsf.med.harvard.edu/core/www/modules/sdig/index.php?value=22/72&pvkey=&run_id=92258&search_id=93772&scans_id=92254&peptide_id=7640&charge=3&scanf=16656&mz_l=276.219&mz_h=476.219) \| **-0.988** \| **3** \| \| **Q** \| **18** \| **---** \| [1886.968](https://tmsf.med.harvard.edu/core/www/modules/sdig/index.php?value=22/72&pvkey=&run_id=92258&search_id=93772&scans_id=92254&peptide_id=7640&charge=3&scanf=16656&mz_l=1786.968&mz_h=1986.968) \| [**275.171**](https://tmsf.med.harvard.edu/core/www/modules/sdig/index.php?value=22/72&pvkey=&run_id=92258&search_id=93772&scans_id=92254&peptide_id=7640&charge=3&scanf=16656&mz_l=175.171&mz_h=375.171) \| **-0.870** \| **2** \| \| **K** \| **19** \| **---** \| --- \|  \|  \|  \| |

**Sequence no 17**

| [>tr\|A0A5E4F729\|A0A5E4F729_PRUDU Lactoylglutathione lyase OS=Prunus dulcis OX=3755 GN=ALMOND_2B021434 PE=3 SV=1](http://www.uniprot.org/uniprot/?query=A0A5E4F729_PRUDU) |
| --- |
| MSEAEAKSAA PSAELLEWPK KDNRRLLHAV YRVGDLDRTI KFYTEALGMK LLRKRDIPEE KYSNAFLGFG PEESHFVVEL TYNYGVSSYD IGTGFGHFAI  ATPDVKKLVE EVRAKGGNVT REPGPVKGGN SIIAFVKDPD GYTFEIIQRP STPEPLCQVM LRVGDLERSI KFYEKALGLK LLRTIERPEY KYNIAILGYA  EEDQTTILEL TYNYGVTEYT KGNAYAQIAI GTDDVYKSAE VVNLVTQELG GKITRQPGPI PGLNTKITSF LDPDGWKTVL VDNEDFLKEL Q |

| Legend | | |
| --- | --- | --- |
| THISREFERE NCE | UNMATCHED | OTHERREFER ENCES |

| \| 32579.87 Da \| Protein Coverage \| \| \| --- \| --- \| --- \| \| 291 AA \| 39 AA \| **13.40 %** \| |
| --- | --- | --- | --- | --- | --- | --- |

**Data table 22**

| \| **Sequence** \| \| --- \| \| SAEVVNLVTQELGGK \| |
| --- | --- | --- |
| **Predicted Fragmentation Pattern**   \| **Seq** \| **#** \| **b: Δ Error** \| **b** \| **y** \| **y: Δ Error** \| **+1** \| \| --- \| --- \| --- \| --- \| --- \| --- \| --- \| \| **S** \| **1** \| **---** \| [88.039](https://tmsf.med.harvard.edu/core/www/modules/sdig/index.php?value=17/28&pvkey=&run_id=92258&search_id=93772&scans_id=92254&peptide_id=5761&charge=2&scanf=14831&mz_l=-11.961&mz_h=188.039) \| --- \| **---** \| **15** \| \| **A** \| **2** \| **-0.110** \| [**159.076**](https://tmsf.med.harvard.edu/core/www/modules/sdig/index.php?value=17/28&pvkey=&run_id=92258&search_id=93772&scans_id=92254&peptide_id=5761&charge=2&scanf=14831&mz_l=59.076&mz_h=259.076) \| [1456.801](https://tmsf.med.harvard.edu/core/www/modules/sdig/index.php?value=17/28&pvkey=&run_id=92258&search_id=93772&scans_id=92254&peptide_id=5761&charge=2&scanf=14831&mz_l=1356.801&mz_h=1556.801) \| **---** \| **14** \| \| **E** \| **3** \| **-0.398** \| [**288.119**](https://tmsf.med.harvard.edu/core/www/modules/sdig/index.php?value=17/28&pvkey=&run_id=92258&search_id=93772&scans_id=92254&peptide_id=5761&charge=2&scanf=14831&mz_l=188.119&mz_h=388.119) \| [**1385.763**](https://tmsf.med.harvard.edu/core/www/modules/sdig/index.php?value=17/28&pvkey=&run_id=92258&search_id=93772&scans_id=92254&peptide_id=5761&charge=2&scanf=14831&mz_l=1285.763&mz_h=1485.763) \| **-5.493** \| **13** \| \| **V** \| **4** \| **-0.280** \| [**387.187**](https://tmsf.med.harvard.edu/core/www/modules/sdig/index.php?value=17/28&pvkey=&run_id=92258&search_id=93772&scans_id=92254&peptide_id=5761&charge=2&scanf=14831&mz_l=287.187&mz_h=487.187) \| [**1256.721**](https://tmsf.med.harvard.edu/core/www/modules/sdig/index.php?value=17/28&pvkey=&run_id=92258&search_id=93772&scans_id=92254&peptide_id=5761&charge=2&scanf=14831&mz_l=1156.721&mz_h=1356.721) \| **0.929** \| **12** \| \| **V** \| **5** \| **-2.469** \| [**486.256**](https://tmsf.med.harvard.edu/core/www/modules/sdig/index.php?value=17/28&pvkey=&run_id=92258&search_id=93772&scans_id=92254&peptide_id=5761&charge=2&scanf=14831&mz_l=386.256&mz_h=586.256) \| [**1157.652**](https://tmsf.med.harvard.edu/core/www/modules/sdig/index.php?value=17/28&pvkey=&run_id=92258&search_id=93772&scans_id=92254&peptide_id=5761&charge=2&scanf=14831&mz_l=1057.652&mz_h=1257.652) \| **-0.737** \| **11** \| \| **N** \| **6** \| **-0.508** \| [**600.299**](https://tmsf.med.harvard.edu/core/www/modules/sdig/index.php?value=17/28&pvkey=&run_id=92258&search_id=93772&scans_id=92254&peptide_id=5761&charge=2&scanf=14831&mz_l=500.299&mz_h=700.299) \| [**1058.584**](https://tmsf.med.harvard.edu/core/www/modules/sdig/index.php?value=17/28&pvkey=&run_id=92258&search_id=93772&scans_id=92254&peptide_id=5761&charge=2&scanf=14831&mz_l=958.584&mz_h=1158.584) \| **-0.293** \| **10** \| \| **L** \| **7** \| **0.574** \| [**713.383**](https://tmsf.med.harvard.edu/core/www/modules/sdig/index.php?value=17/28&pvkey=&run_id=92258&search_id=93772&scans_id=92254&peptide_id=5761&charge=2&scanf=14831&mz_l=613.383&mz_h=813.383) \| [**944.541**](https://tmsf.med.harvard.edu/core/www/modules/sdig/index.php?value=17/28&pvkey=&run_id=92258&search_id=93772&scans_id=92254&peptide_id=5761&charge=2&scanf=14831&mz_l=844.541&mz_h=1044.541) \| **0.274** \| **9** \| \| **V** \| **8** \| **1.338** \| [**812.451**](https://tmsf.med.harvard.edu/core/www/modules/sdig/index.php?value=17/28&pvkey=&run_id=92258&search_id=93772&scans_id=92254&peptide_id=5761&charge=2&scanf=14831&mz_l=712.451&mz_h=912.451) \| [**831.457**](https://tmsf.med.harvard.edu/core/www/modules/sdig/index.php?value=17/28&pvkey=&run_id=92258&search_id=93772&scans_id=92254&peptide_id=5761&charge=2&scanf=14831&mz_l=731.457&mz_h=931.457) \| **-0.254** \| **8** \| \| **T** \| **9** \| **---** \| [913.499](https://tmsf.med.harvard.edu/core/www/modules/sdig/index.php?value=17/28&pvkey=&run_id=92258&search_id=93772&scans_id=92254&peptide_id=5761&charge=2&scanf=14831&mz_l=813.499&mz_h=1013.499) \| [**732.389**](https://tmsf.med.harvard.edu/core/www/modules/sdig/index.php?value=17/28&pvkey=&run_id=92258&search_id=93772&scans_id=92254&peptide_id=5761&charge=2&scanf=14831&mz_l=632.389&mz_h=832.389) \| **-0.130** \| **7** \| \| **Q** \| **10** \| **2.573** \| [**1041.558**](https://tmsf.med.harvard.edu/core/www/modules/sdig/index.php?value=17/28&pvkey=&run_id=92258&search_id=93772&scans_id=92254&peptide_id=5761&charge=2&scanf=14831&mz_l=941.558&mz_h=1141.558) \| [**631.341**](https://tmsf.med.harvard.edu/core/www/modules/sdig/index.php?value=17/28&pvkey=&run_id=92258&search_id=93772&scans_id=92254&peptide_id=5761&charge=2&scanf=14831&mz_l=531.341&mz_h=731.341) \| **0.735** \| **6** \| \| **E** \| **11** \| **-2.916** \| [**1170.600**](https://tmsf.med.harvard.edu/core/www/modules/sdig/index.php?value=17/28&pvkey=&run_id=92258&search_id=93772&scans_id=92254&peptide_id=5761&charge=2&scanf=14831&mz_l=1070.6&mz_h=1270.6) \| [**503.282**](https://tmsf.med.harvard.edu/core/www/modules/sdig/index.php?value=17/28&pvkey=&run_id=92258&search_id=93772&scans_id=92254&peptide_id=5761&charge=2&scanf=14831&mz_l=403.282&mz_h=603.282) \| **0.223** \| **5** \| \| **L** \| **12** \| **---** \| [1283.684](https://tmsf.med.harvard.edu/core/www/modules/sdig/index.php?value=17/28&pvkey=&run_id=92258&search_id=93772&scans_id=92254&peptide_id=5761&charge=2&scanf=14831&mz_l=1183.684&mz_h=1383.684) \| [**374.240**](https://tmsf.med.harvard.edu/core/www/modules/sdig/index.php?value=17/28&pvkey=&run_id=92258&search_id=93772&scans_id=92254&peptide_id=5761&charge=2&scanf=14831&mz_l=274.24&mz_h=474.24) \| **-0.541** \| **4** \| \| **G** \| **13** \| **---** \| [1340.706](https://tmsf.med.harvard.edu/core/www/modules/sdig/index.php?value=17/28&pvkey=&run_id=92258&search_id=93772&scans_id=92254&peptide_id=5761&charge=2&scanf=14831&mz_l=1240.706&mz_h=1440.706) \| [**261.156**](https://tmsf.med.harvard.edu/core/www/modules/sdig/index.php?value=17/28&pvkey=&run_id=92258&search_id=93772&scans_id=92254&peptide_id=5761&charge=2&scanf=14831&mz_l=161.156&mz_h=361.156) \| **-0.237** \| **3** \| \| **G** \| **14** \| **---** \| [1397.727](https://tmsf.med.harvard.edu/core/www/modules/sdig/index.php?value=17/28&pvkey=&run_id=92258&search_id=93772&scans_id=92254&peptide_id=5761&charge=2&scanf=14831&mz_l=1297.727&mz_h=1497.727) \| [**204.134**](https://tmsf.med.harvard.edu/core/www/modules/sdig/index.php?value=17/28&pvkey=&run_id=92258&search_id=93772&scans_id=92254&peptide_id=5761&charge=2&scanf=14831&mz_l=104.134&mz_h=304.134) \| **-0.628** \| **2** \| \| **K** \| **15** \| **---** \| --- \| [**147.113**](https://tmsf.med.harvard.edu/core/www/modules/sdig/index.php?value=17/28&pvkey=&run_id=92258&search_id=93772&scans_id=92254&peptide_id=5761&charge=2&scanf=14831&mz_l=47.113&mz_h=247.113) \| **-0.182** \| **1** \| \|  \|  \|  \|  \|  \|  \|  \| |

**Sequence no 18**

| [>tr\|A0A5E4FRA1\|A0A5E4FRA1_PRUDU PREDICTED: L-ascorbate oxidase OS=Prunus dulcis OX=3755 GN=ALMOND_2B032635 PE=3 SV=1](http://www.uniprot.org/uniprot/?query=A0A5E4FRA1_PRUDU) |
| --- |
| MLLKFLAAFL LVLGFATAED PYRFFDWNIT YGDIYPLGVR QQGILINGQF PGPEIYSVTN DNLIINVHNS LPEPFLISWN GVQHRRNSYQ DGVYGTTCPI  PPGKNFTYTL QVKDQIGSFY YFPSLAFHKA AGGFGAIKIL SRPRIPVPFP DPVGDYSILI GDWHKTDHKI LKGILDRGHR LPFPDGIIIN GRGPNGTYFT  FDQGKTYRLR ISNVGLQNSL NFRIQGHKLK LVEVEGTHTI QTTYDSLDIH VGQSYSVLVT ADQAPQDYYI AVSTRFTSQV LTSTAVFHYS NSGRQVSGPI  PAGPTTQTGW SLSQALSIRT NLTASGPRPN PQGSYHYGLV NVSRTIKLES SAAQVSGKQR YAVNSVSFIP ADTPLKLADY FKIGGVFKVG SISDNPTGQK  MYLDTSVMGA DFRAFVEIVF QNHENIVQSW HLDGHSFWVV GMDGGKWTPA SRNEYNLRDA VSRSTTQVYP KSWTAIYIAL DNVGMWNLRT EFWARQYLGQ  QFYLRVYSPV ESARDEYPIP RNALLCGRAA GRSTRP |

| Legend | | |
| --- | --- | --- |
| THISREFERE NCE | UNMATCHED | OTHERREFER ENCES |

| \| 59858.55 Da \| Protein Coverage \| \| \| --- \| --- \| --- \| \| 536 AA \| 39 AA \| **7.28 %** \| |
| --- | --- | --- | --- | --- | --- | --- |

**Data table 23**

| \| **Sequence** \| \| --- \| \| ISNVGLQNSLNFR \| |
| --- | --- | --- |
| **Predicted Fragmentation Pattern**   \| **Seq** \| **#** \| **b: Δ Error** \| **b** \| **y** \| **y: Δ Error** \| **+1** \| \| --- \| --- \| --- \| --- \| --- \| --- \| --- \| \| **I** \| **1** \| **---** \| [114.091](https://tmsf.med.harvard.edu/core/www/modules/sdig/index.php?value=16/24&pvkey=&run_id=92258&search_id=93772&scans_id=92254&peptide_id=2809&charge=2&scanf=11934&mz_l=14.091&mz_h=214.091) \| --- \| **---** \| **13** \| \| **S** \| **2** \| **0.450** \| [**201.123**](https://tmsf.med.harvard.edu/core/www/modules/sdig/index.php?value=16/24&pvkey=&run_id=92258&search_id=93772&scans_id=92254&peptide_id=2809&charge=2&scanf=11934&mz_l=101.123&mz_h=301.123) \| [**1348.697**](https://tmsf.med.harvard.edu/core/www/modules/sdig/index.php?value=16/24&pvkey=&run_id=92258&search_id=93772&scans_id=92254&peptide_id=2809&charge=2&scanf=11934&mz_l=1248.697&mz_h=1448.697) \| **1.349** \| **12** \| \| **N** \| **3** \| **-0.405** \| [**315.166**](https://tmsf.med.harvard.edu/core/www/modules/sdig/index.php?value=16/24&pvkey=&run_id=92258&search_id=93772&scans_id=92254&peptide_id=2809&charge=2&scanf=11934&mz_l=215.166&mz_h=415.166) \| [**1261.665**](https://tmsf.med.harvard.edu/core/www/modules/sdig/index.php?value=16/24&pvkey=&run_id=92258&search_id=93772&scans_id=92254&peptide_id=2809&charge=2&scanf=11934&mz_l=1161.665&mz_h=1361.665) \| **-0.650** \| **11** \| \| **V** \| **4** \| **1.328** \| [**414.235**](https://tmsf.med.harvard.edu/core/www/modules/sdig/index.php?value=16/24&pvkey=&run_id=92258&search_id=93772&scans_id=92254&peptide_id=2809&charge=2&scanf=11934&mz_l=314.235&mz_h=514.235) \| [**1147.622**](https://tmsf.med.harvard.edu/core/www/modules/sdig/index.php?value=16/24&pvkey=&run_id=92258&search_id=93772&scans_id=92254&peptide_id=2809&charge=2&scanf=11934&mz_l=1047.622&mz_h=1247.622) \| **-0.006** \| **10** \| \| **G** \| **5** \| **-1.638** \| [**471.256**](https://tmsf.med.harvard.edu/core/www/modules/sdig/index.php?value=16/24&pvkey=&run_id=92258&search_id=93772&scans_id=92254&peptide_id=2809&charge=2&scanf=11934&mz_l=371.256&mz_h=571.256) \| [**1048.553**](https://tmsf.med.harvard.edu/core/www/modules/sdig/index.php?value=16/24&pvkey=&run_id=92258&search_id=93772&scans_id=92254&peptide_id=2809&charge=2&scanf=11934&mz_l=948.553&mz_h=1148.553) \| **-0.536** \| **9** \| \| **L** \| **6** \| **8.936** \| [**584.340**](https://tmsf.med.harvard.edu/core/www/modules/sdig/index.php?value=16/24&pvkey=&run_id=92258&search_id=93772&scans_id=92254&peptide_id=2809&charge=2&scanf=11934&mz_l=484.34&mz_h=684.34) \| [**991.532**](https://tmsf.med.harvard.edu/core/www/modules/sdig/index.php?value=16/24&pvkey=&run_id=92258&search_id=93772&scans_id=92254&peptide_id=2809&charge=2&scanf=11934&mz_l=891.532&mz_h=1091.532) \| **0.151** \| **8** \| \| **Q** \| **7** \| **---** \| [712.399](https://tmsf.med.harvard.edu/core/www/modules/sdig/index.php?value=16/24&pvkey=&run_id=92258&search_id=93772&scans_id=92254&peptide_id=2809&charge=2&scanf=11934&mz_l=612.399&mz_h=812.399) \| [**878.448**](https://tmsf.med.harvard.edu/core/www/modules/sdig/index.php?value=16/24&pvkey=&run_id=92258&search_id=93772&scans_id=92254&peptide_id=2809&charge=2&scanf=11934&mz_l=778.448&mz_h=978.448) \| **-0.226** \| **7** \| \| **N** \| **8** \| **---** \| [826.442](https://tmsf.med.harvard.edu/core/www/modules/sdig/index.php?value=16/24&pvkey=&run_id=92258&search_id=93772&scans_id=92254&peptide_id=2809&charge=2&scanf=11934&mz_l=726.442&mz_h=926.442) \| [**750.389**](https://tmsf.med.harvard.edu/core/www/modules/sdig/index.php?value=16/24&pvkey=&run_id=92258&search_id=93772&scans_id=92254&peptide_id=2809&charge=2&scanf=11934&mz_l=650.389&mz_h=850.389) \| **-0.123** \| **6** \| \| **S** \| **9** \| **-9.706** \| [**913.474**](https://tmsf.med.harvard.edu/core/www/modules/sdig/index.php?value=16/24&pvkey=&run_id=92258&search_id=93772&scans_id=92254&peptide_id=2809&charge=2&scanf=11934&mz_l=813.474&mz_h=1013.474) \| [**636.346**](https://tmsf.med.harvard.edu/core/www/modules/sdig/index.php?value=16/24&pvkey=&run_id=92258&search_id=93772&scans_id=92254&peptide_id=2809&charge=2&scanf=11934&mz_l=536.346&mz_h=736.346) \| **0.461** \| **5** \| \| **L** \| **10** \| **---** \| [1026.558](https://tmsf.med.harvard.edu/core/www/modules/sdig/index.php?value=16/24&pvkey=&run_id=92258&search_id=93772&scans_id=92254&peptide_id=2809&charge=2&scanf=11934&mz_l=926.558&mz_h=1126.558) \| [**549.314**](https://tmsf.med.harvard.edu/core/www/modules/sdig/index.php?value=16/24&pvkey=&run_id=92258&search_id=93772&scans_id=92254&peptide_id=2809&charge=2&scanf=11934&mz_l=449.314&mz_h=649.314) \| **-2.493** \| **4** \| \| **N** \| **11** \| **---** \| [1140.601](https://tmsf.med.harvard.edu/core/www/modules/sdig/index.php?value=16/24&pvkey=&run_id=92258&search_id=93772&scans_id=92254&peptide_id=2809&charge=2&scanf=11934&mz_l=1040.601&mz_h=1240.601) \| [**436.230**](https://tmsf.med.harvard.edu/core/www/modules/sdig/index.php?value=16/24&pvkey=&run_id=92258&search_id=93772&scans_id=92254&peptide_id=2809&charge=2&scanf=11934&mz_l=336.23&mz_h=536.23) \| **-2.607** \| **3** \| \| **F** \| **12** \| **---** \| [1287.669](https://tmsf.med.harvard.edu/core/www/modules/sdig/index.php?value=16/24&pvkey=&run_id=92258&search_id=93772&scans_id=92254&peptide_id=2809&charge=2&scanf=11934&mz_l=1187.669&mz_h=1387.669) \| [**322.187**](https://tmsf.med.harvard.edu/core/www/modules/sdig/index.php?value=16/24&pvkey=&run_id=92258&search_id=93772&scans_id=92254&peptide_id=2809&charge=2&scanf=11934&mz_l=222.187&mz_h=422.187) \| **0.793** \| **2** \| \| **R** \| **13** \| **---** \| --- \| [**175.119**](https://tmsf.med.harvard.edu/core/www/modules/sdig/index.php?value=16/24&pvkey=&run_id=92258&search_id=93772&scans_id=92254&peptide_id=2809&charge=2&scanf=11934&mz_l=75.119&mz_h=275.119) \| **-0.233** \| **1** \| |

**Sequence no 19**

| [>tr\|A0A1U8J2X7\|A0A1U8J2X7_GOSHI Senescence-specific cysteine protease SAG39-like OS=Gossypium hirsutum OX=3635 GN=LOC107903163 PE=3 SV=1](http://www.uniprot.org/uniprot/?query=A0A1U8J2X7_GOSHI) |
| --- |
| MASISQCLFL IFVLGIWASQ ASSRSVPEVS MSDRFEQWVS SYSRLYQDAA EKEKRFQIFK ENMEYIEYHN ADANKKYKLG VNEFTDLTNE EFKAMRNGYK  MRSSNTVAAS KTTSFRYENV SAVPSSMDWR KKGAVTGIKD QGQCGCCWAF SAVAAMEGIS KLKTGTLISL SEQELVDCDV NGEDQGCNGG LMDDAFEFII  SNKGLTTETN YPYEGVDGSC NKKKSANHAA KITGFEDVPS NSESALLKAV ANQPVSVAID AGGSDFQHYK SGVFTGECTT FLDHGVTTVG YGEAEDGTKY  WLVKNSWGTS WGEDGYIRMQ RDIDAAEGLC GIAMEASYPT A |

| Legend | | |
| --- | --- | --- |
| THISREFERE NCE | UNMATCHED | OTHERREFER ENCES |

| \| 37407.76 Da \| Protein Coverage \| \| \| --- \| --- \| --- \| \| 341 AA \| 14 AA \| **4.11 %** \| |
| --- | --- | --- | --- | --- | --- | --- |

**Data table 24**

| \| **Sequence** \| \| --- \| \| NSWGTSWGEDGYIR \| |
| --- | --- | --- |
| **Predicted Fragmentation Pattern**   \| **Seq** \| **#** \| **b: Δ Error** \| **b** \| **y** \| **y: Δ Error** \| **+1** \| \| --- \| --- \| --- \| --- \| --- \| --- \| --- \| \| **N** \| **1** \| **-0.349** \| [**115.050**](https://tmsf.med.harvard.edu/core/www/modules/sdig/index.php?value=15/26&pvkey=&run_id=92259&search_id=93773&scans_id=92255&peptide_id=2827&charge=2&scanf=11951&mz_l=15.05&mz_h=215.05) \| --- \| **---** \| **14** \| \| **S** \| **2** \| **-0.540** \| [**202.082**](https://tmsf.med.harvard.edu/core/www/modules/sdig/index.php?value=15/26&pvkey=&run_id=92259&search_id=93773&scans_id=92255&peptide_id=2827&charge=2&scanf=11951&mz_l=102.082&mz_h=302.082) \| [**1513.671**](https://tmsf.med.harvard.edu/core/www/modules/sdig/index.php?value=15/26&pvkey=&run_id=92259&search_id=93773&scans_id=92255&peptide_id=2827&charge=2&scanf=11951&mz_l=1413.671&mz_h=1613.671) \| **-3.454** \| **13** \| \| **W** \| **3** \| **-0.747** \| [**388.162**](https://tmsf.med.harvard.edu/core/www/modules/sdig/index.php?value=15/26&pvkey=&run_id=92259&search_id=93773&scans_id=92255&peptide_id=2827&charge=2&scanf=11951&mz_l=288.162&mz_h=488.162) \| [**1426.639**](https://tmsf.med.harvard.edu/core/www/modules/sdig/index.php?value=15/26&pvkey=&run_id=92259&search_id=93773&scans_id=92255&peptide_id=2827&charge=2&scanf=11951&mz_l=1326.639&mz_h=1526.639) \| **-0.809** \| **12** \| \| **G** \| **4** \| **0.149** \| [**445.183**](https://tmsf.med.harvard.edu/core/www/modules/sdig/index.php?value=15/26&pvkey=&run_id=92259&search_id=93773&scans_id=92255&peptide_id=2827&charge=2&scanf=11951&mz_l=345.183&mz_h=545.183) \| [**1240.559**](https://tmsf.med.harvard.edu/core/www/modules/sdig/index.php?value=15/26&pvkey=&run_id=92259&search_id=93773&scans_id=92255&peptide_id=2827&charge=2&scanf=11951&mz_l=1140.559&mz_h=1340.559) \| **-1.153** \| **11** \| \| **T** \| **5** \| **-0.623** \| [**546.231**](https://tmsf.med.harvard.edu/core/www/modules/sdig/index.php?value=15/26&pvkey=&run_id=92259&search_id=93773&scans_id=92255&peptide_id=2827&charge=2&scanf=11951&mz_l=446.231&mz_h=646.231) \| [**1183.538**](https://tmsf.med.harvard.edu/core/www/modules/sdig/index.php?value=15/26&pvkey=&run_id=92259&search_id=93773&scans_id=92255&peptide_id=2827&charge=2&scanf=11951&mz_l=1083.538&mz_h=1283.538) \| **-1.123** \| **10** \| \| **S** \| **6** \| **-9.477** \| [**633.263**](https://tmsf.med.harvard.edu/core/www/modules/sdig/index.php?value=15/26&pvkey=&run_id=92259&search_id=93773&scans_id=92255&peptide_id=2827&charge=2&scanf=11951&mz_l=533.263&mz_h=733.263) \| [**1082.490**](https://tmsf.med.harvard.edu/core/www/modules/sdig/index.php?value=15/26&pvkey=&run_id=92259&search_id=93773&scans_id=92255&peptide_id=2827&charge=2&scanf=11951&mz_l=982.49&mz_h=1182.49) \| **-0.937** \| **9** \| \| **W** \| **7** \| **1.803** \| [**819.342**](https://tmsf.med.harvard.edu/core/www/modules/sdig/index.php?value=15/26&pvkey=&run_id=92259&search_id=93773&scans_id=92255&peptide_id=2827&charge=2&scanf=11951&mz_l=719.342&mz_h=919.342) \| [**995.458**](https://tmsf.med.harvard.edu/core/www/modules/sdig/index.php?value=15/26&pvkey=&run_id=92259&search_id=93773&scans_id=92255&peptide_id=2827&charge=2&scanf=11951&mz_l=895.458&mz_h=1095.458) \| **-0.850** \| **8** \| \| **G** \| **8** \| **---** \| [876.363](https://tmsf.med.harvard.edu/core/www/modules/sdig/index.php?value=15/26&pvkey=&run_id=92259&search_id=93773&scans_id=92255&peptide_id=2827&charge=2&scanf=11951&mz_l=776.363&mz_h=976.363) \| [**809.379**](https://tmsf.med.harvard.edu/core/www/modules/sdig/index.php?value=15/26&pvkey=&run_id=92259&search_id=93773&scans_id=92255&peptide_id=2827&charge=2&scanf=11951&mz_l=709.379&mz_h=909.379) \| **-1.011** \| **7** \| \| **E** \| **9** \| **---** \| [1005.406](https://tmsf.med.harvard.edu/core/www/modules/sdig/index.php?value=15/26&pvkey=&run_id=92259&search_id=93773&scans_id=92255&peptide_id=2827&charge=2&scanf=11951&mz_l=905.406&mz_h=1105.406) \| [**752.357**](https://tmsf.med.harvard.edu/core/www/modules/sdig/index.php?value=15/26&pvkey=&run_id=92259&search_id=93773&scans_id=92255&peptide_id=2827&charge=2&scanf=11951&mz_l=652.357&mz_h=852.357) \| **1.806** \| **6** \| \| **D** \| **10** \| **---** \| [1120.433](https://tmsf.med.harvard.edu/core/www/modules/sdig/index.php?value=15/26&pvkey=&run_id=92259&search_id=93773&scans_id=92255&peptide_id=2827&charge=2&scanf=11951&mz_l=1020.433&mz_h=1220.433) \| [**623.315**](https://tmsf.med.harvard.edu/core/www/modules/sdig/index.php?value=15/26&pvkey=&run_id=92259&search_id=93773&scans_id=92255&peptide_id=2827&charge=2&scanf=11951&mz_l=523.315&mz_h=723.315) \| **-1.067** \| **5** \| \| **G** \| **11** \| **---** \| [1177.454](https://tmsf.med.harvard.edu/core/www/modules/sdig/index.php?value=15/26&pvkey=&run_id=92259&search_id=93773&scans_id=92255&peptide_id=2827&charge=2&scanf=11951&mz_l=1077.454&mz_h=1277.454) \| [**508.288**](https://tmsf.med.harvard.edu/core/www/modules/sdig/index.php?value=15/26&pvkey=&run_id=92259&search_id=93773&scans_id=92255&peptide_id=2827&charge=2&scanf=11951&mz_l=408.288&mz_h=608.288) \| **-0.896** \| **4** \| \| **Y** \| **12** \| **---** \| [1340.518](https://tmsf.med.harvard.edu/core/www/modules/sdig/index.php?value=15/26&pvkey=&run_id=92259&search_id=93773&scans_id=92255&peptide_id=2827&charge=2&scanf=11951&mz_l=1240.518&mz_h=1440.518) \| [**451.266**](https://tmsf.med.harvard.edu/core/www/modules/sdig/index.php?value=15/26&pvkey=&run_id=92259&search_id=93773&scans_id=92255&peptide_id=2827&charge=2&scanf=11951&mz_l=351.266&mz_h=551.266) \| **-2.948** \| **3** \| \| **I** \| **13** \| **---** \| [1453.602](https://tmsf.med.harvard.edu/core/www/modules/sdig/index.php?value=15/26&pvkey=&run_id=92259&search_id=93773&scans_id=92255&peptide_id=2827&charge=2&scanf=11951&mz_l=1353.602&mz_h=1553.602) \| [**288.203**](https://tmsf.med.harvard.edu/core/www/modules/sdig/index.php?value=15/26&pvkey=&run_id=92259&search_id=93773&scans_id=92255&peptide_id=2827&charge=2&scanf=11951&mz_l=188.203&mz_h=388.203) \| **-0.682** \| **2** \| \| **R** \| **14** \| **---** \| --- \| [**175.119**](https://tmsf.med.harvard.edu/core/www/modules/sdig/index.php?value=15/26&pvkey=&run_id=92259&search_id=93773&scans_id=92255&peptide_id=2827&charge=2&scanf=11951&mz_l=75.119&mz_h=275.119) \| **-0.843** \| **1** \| |
